# Changes in incarceration and tuberculosis notifications from prisons during the COVID-19 pandemic in Europe and the Americas: a time-series analysis of national surveillance data

**DOI:** 10.1016/S2468-2667(24)00325-6

**Published:** 2025-03-31

**Authors:** Amy Zheng, Lena Faust, Anthony D Harries, Pedro Avedillo, Michael Akodu, Miranda Galvan, Beatriz Barreto-Duarte, Bruno B Andrade, César Ugarte-Gil, Alberto L Garcia-Basteiro, Marcos Espinal, Joshua L Warren, Leonardo Martinez

**Affiliations:** a Department of Epidemiology, Boston University School of Public Health, Boston, MA, USA; b Department of Global Health, Boston University School of Public Health, Boston, MA, USA; c Faculty of Infectious and Tropical Diseases, London School of Hygiene and Tropical Medicine, London, UK; d International Union Against Tuberculosis and Lung Disease, Paris, France; e Pan American Health Organization, Communicable Diseases and Environmental Determinants of Health, Washington, DC, USA; f Laboratório de Pesquisa Clínica e Translacional, Instituto Gonçalo Moniz, Fundação Oswaldo Cruz, Salvador, Brazil; g Institute for Research in Priority Populations, Multinational Organization Network Sponsoring Translational and Epidemiological Research Initiative, Salvador, Brazil; h Instituto de Pesquisa Clínica e Translacional, Faculdade Zarns, Clariens Educação, Salvador, Brazil; i Department of Epidemiology, School of Public and Population Health, University of Texas Medical Branch, Galveston, TX, USA; j Centro de Investigação em Saúde de Manhiça, Maputo, Mozambique; k Centro de Investigación Biomédica en Red de Enfermedades Infecciosas, Barcelona, Spain; l ISGlobal, Hospital Clínic, Universitat de Barcelona, Barcelona, Spain; m Department of Biostatistics, Yale School of Public Health, New Haven, CT, USA

## Abstract

**Background:**

The COVID-19 pandemic disrupted tuberculosis control programmes globally; whether or not this disproportionately affected people who were incarcerated is unknown. We aimed to evaluate changes in incarceration and tuberculosis notifications in prisons in Europe and the Americas during the COVID-19 pandemic.

**Methods:**

Data from WHO Pan American Health Organization (PAHO) and WHO Europe were used to conduct a joint hierarchical Bayesian negative binomial time-series. This approach accounted for world region, country-specific temporal trends, and country-specific autocorrelated random effects to simultaneously model and predict both annual prison population (ie, the offset) and prison tuberculosis cases (ie, the primary outcome). Results were used to calculate percentage differences between predicted and observed annual tuberculosis notifications and prison populations during the COVID-19 pandemic years (2020–22).

**Findings:**

In total, 22 of 39 countries from PAHO and 25 of 53 countries from WHO Europe were included (representing 4·9 million people incarcerated annually), contributing 520 country-years of follow-up. Observed tuberculosis notifications in prisons were lower than predicted in 2020 (–26·2% [95% credible interval –66·3 to 7·8), 2021 (–46·4% [–108·8 to 3·9]), and 2022 (–48·9 [–124·4 to 10·3]). These decreasing trends were consistent across Europe and the Americas, but larger decreases were seen in low-burden settings in 2020 (–54·8% [–112·4 to –4·8]) and 2021 (–68·4% [–156·6 to –2·9]), high-burden settings in 2021 (–89·4% [–190·3 to –10·4]), and Central and North America in 2021 (–100·3% [–239·0 to –6·3]). Observed incarceration levels were similar to predicted levels (<10% difference overall) during all COVID-19 pandemic years.

**Interpretation:**

Tuberculosis notifications in prisons from 47 countries in Europe and the Americas were lower than expected (at times >50% lower) during COVID-19 pandemic years, despite consistent incarceration levels. Reasons for this change in tuberculosis notifications might be multifactorial and include missed diagnoses and implementation of COVID-19 pandemic measures, reducing transmission. Greater prioritisation of people who are incarcerated is needed to ensure appropriate access to care in the face of future pandemics.

**Funding:**

Canadian Institutes of Health Research, National Institutes of Health, and Oswaldo Cruz Foundation, Brazil.

## Introduction

An estimated 10·6 million people developed tuberculosis globally in 2022.^1^ People who are incarcerated have a high burden of tuberculosis^2^, ^3^, ^4^, ^5^, ^6^ and have been historically neglected,^7^, ^8^, ^9^ despite their importance as a priority population in helping countries achieve tuberculosis elimination targets.^6^ In many countries, prisons are under-resourced and do not have adequate access to quality medical services, in part due to the deprioritisation and fragmentation of health service delivery in prisons.^5^, ^7^, ^10^, ^11^, ^12^ Moreover, factors prevalent in the carceral environment (eg, overcrowding^11^, ^13^ and poor ventilation^10^) facilitate the transmission of respiratory diseases, such as tuberculosis. These environmental conditions, in combination with individual risk factors (eg, HIV, smoking, drug use, and living in a high-burden community^14^, ^15^), put incarcerated populations at higher risk of *Mycobacterium tuberculosis* infection and disease than the general population.

Globally, national tuberculosis programmes were disrupted by the COVID-19 pandemic.^16^, ^17^, ^18^ Major interruptions in tuberculosis care among the general population were seen, leading to sharp decreases in case detection in the first year of the pandemic.^16^ Although many countries have shown recovery in tuberculosis notifications and rates among the general population,^1^, ^19^, ^20^ the effect of the pandemic on tuberculosis programmes in prisons is unknown. The COVID-19 pandemic might have affected tuberculosis control and management in the carceral setting via several mechanisms. First, governments shifting their priorities to COVID-19-related issues might have resulted in a diversion of resources from prisons,^21^, ^22^ exacerbating staffing shortages within prisons and leading to disruptions in prison tuberculosis programmes.^13^ Second, decarceration and physical distancing might have led to decreased tuberculosis exposure and transmission (decarceration might have also led to reductions in the incarcerated population).^13^ However, not all countries implemented decarceration policies, and the degree of decarceration within and across countries and time is not well understood.^19^, ^20^, ^21^, ^22^, ^23^, ^24^, ^25^ Furthermore, reductions of medical services or prison staff within prisons could have led to poorer tuberculosis detection in some countries, leading to increased *M tuberculosis* transmission.^13^

**Research in context**

**Evidence before this study**

Previous studies evaluating changes in incarceration and tuberculosis notifications during the COVID-19 pandemic were identified by searching PubMed using the following search terms: (incarceration OR prison) AND (tuberculosis OR TB) AND (COVID-19 OR pandemic) with no restrictions on language from database inception to Sept 1, 2024. This search yielded 29 results. Many of the studies were not specific to the carceral setting but emphasised the need to prioritise screening, diagnosis, and treatment of tuberculosis for all groups as well as the importance of collaboration across agencies and governmental organisations. The three studies (USA, Brazil, and Peru) that evaluated the effect of the COVID-19 pandemic on tuberculosis identified decreases in tuberculosis diagnoses in the first year of the pandemic.

**Added value of this study**

To our knowledge, this is the first multi-country study estimating changes in incarceration and tuberculosis notifications during the COVID-19 pandemic in the Americas and Europe across the first three years (2020–22) of the pandemic. Our study includes 47 countries across these two regions, representing approximately 4·9 million people incarcerated annually. Our results suggest that there were notable decreases (compared with counterfactual estimates) in tuberculosis notifications in both regions, whereas incarceration levels were not appreciably affected during pandemic years. Tuberculosis notifications were lower than counterfactual estimates throughout all 3 years of the study period. This finding suggests that other factors—such as delays in diagnoses, missed diagnoses, or successful implementation of physical distancing measures to reduce COVID-19 transmission—might have contributed to the observed decrease in tuberculosis notifications.

**Implications of all the available evidence**

Current evidence suggests that many countries have had decreases in tuberculosis notifications in prisons during the COVID-19 pandemic. Further empirical evidence is needed regarding the mechanism for these observed effects on tuberculosis notification. Regardless of the mechanisms, prioritising screening and diagnosis of tuberculosis among this highly vulnerable and historically neglected population is urgently needed.

In the context of these highlighted challenges for tuberculosis diagnosis and care in this key population, we aimed to investigate annual trends in incarceration levels and tuberculosis notifications among people who were incarcerated in Europe and the Americas before the COVID-19 pandemic (pre-2020) and during the COVID-19 pandemic (2020–22). We investigated whether incarceration levels and prison tuberculosis notifications were distinct from expected levels during the COVID-19 pandemic years under a counterfactual scenario.

## Methods

### Data sources

We collected national tuberculosis notification data in prisons from distinct sources. Since 2008, the WHO European office, in collaboration with the European Centers for Disease Control and Prevention (ECDC), has routinely collected and published tuberculosis surveillance data in 53 countries from Europe. These data are reported to ECDC–WHO Europe via a common portal by designated experts such as programme officials from National Tuberculosis Programs within each country. Completeness of data is variable and dependent on each country. Starting in 2012, data on the number of people who were incarcerated and tuberculosis notification rates were published as part of ECDC tuberculosis surveillance reports.^26^ The first data from these initial reports (and within our analysis) were dated back to 2010.^26^

Since 2018, Pan American Health Organization (PAHO) has routinely collected data on tuberculosis notifications in prisons in the Americas through the WHO Global Tuberculosis Data Collection System. These data are reported by each country's National Tuberculosis Program. Although PAHO started collecting these data later than WHO Europe–ECDC, data on the annual number of cases among this population are available dating back to 2000 for PAHO. Completeness and quality of data were variable between countries.

Given that PAHO does not collect data on the size of the incarcerated population and that reported incarceration data from Europe are dependent on each country, missing incarceration level data were supplemented from the Institute for Crime and Justice Policy Research through the World Prison Brief. The World Prison Brief publishes data on incarceration levels, prison capacity (eg, crowding), and number of female prisoners for all countries. These data are collected from various sources—eg, each country's ministry responsible for the prison system, policy makers, researchers, published reports, and civil society organisations. We compiled annual data on the total number of people who are incarcerated, prison capacity, and pre-trial detainees from the World Prison Brief. Crowding levels were calculated by dividing each country's most recently reported number of people who were incarcerated pre-COVID-19 (ie, pre-2020) by prison capacity; with overcrowding defined as crowding levels exceeding 100% of official capacity. Completeness of data is heterogenous and dependent on each country's ministry. Tuberculosis notification cases are determined by the number of notified tuberculosis cases per year in the prison setting for each country as reported by PAHO and ECDC. Tuberculosis notification rates in prisons were calculated by dividing each country's number of tuberculosis cases as reported by PAHO or ECDC by the number of people who were incarcerated as reported by the World Prison Brief. These data have been described previously.^6^

### Statistical analysis

A multivariate hierarchical Bayesian time-series model was developed to predict, for 2020–22, annual prison population totals and prison tuberculosis cases (new and relapsed) simultaneously using data from 2010 to 2019 in countries in the PAHO and WHO European region. Countries in the PAHO and WHO European region were eligible for model inclusion if 4 or more years of pre-COVID-19 pandemic data were reported. Countries with less than 4 years of historical data were excluded due to instability of model estimates. Additionally, countries that had 4 or more years of pre-COVID-19 data but did not report any data during the COVID-19 pandemic were included in the model to improve robustness of the model fit to the pre-COVID-19 period. Both outcomes (prison population and tuberculosis case counts) across all years and countries were modelled simultaneously to leverage shared trends and correlation in the data, and to correctly characterise uncertainty in the final set of predictions, both of which might result in improved predictive performance in COVID-19 years. The model was applied to data from 2010 to 2019 and then used to predict outcomes in 2020–22. These counterfactual predictions were then compared with the observed totals.

Our analysis included one multifaceted hierarchical model. This model included two components that were fitted simultaneously. In the first component of the joint model, a negative binomial regression framework was introduced for the prisoner population totals (ie, first dependent variable) across years for a specific country. The model included an intercept parameter, a country-level random effect to account for repeated measures within a country, and a set of autoregressive random effects to account for autocorrelation in totals across time. The autoregressive random effects are country-specific and independent across countries. Parameters that control the variability and correlation between these effects are shared across countries to improve their estimation, particularly for countries with more missing data.

In the second component of the joint model, a second negative binomial regression model was used for the tuberculosis case counts (ie, second dependent variable) from a given country across the years. Importantly, the offset term (ie, denominator) for this model was the prison population totals and connects both stages of the joint modelling framework. Therefore, when a country had non-missing prison population totals, they were used directly as the offset. However, when these data were missing, the joint model imputed these values and correctly accounted for the increased uncertainty. In addition to the offset term, this model included a country-specific intercept and country-specific linear time-trend slope, a similar country-specific autoregressive random effect as previously described, and a regional indicator variable (Europe–Americas; ie, independent variable). The country-specific intercepts and slopes were jointly modelled to account for correlation between the parameters and to improve robustness of the estimation and inference for countries with sparse data.

The model was fitted using a Markov chain Monte Carlo posterior sampling algorithm via the jags package in R (v4.1.2). Weakly informative previous distributions were selected to allow the data to primarily drive the inference and predictions. After removal of 10 000 iterations in the burn-in period, the model was run for an additional 100 000 iterations, which were thinned by a factor of 10 to reduce posterior autocorrelation, resulting in 10 000 samples for making posterior inference. Convergence was assessed using Geweke's diagnostic^27^ along with visual inspection of individual parameter trace plots, with neither tool indicating any convergence issues. Details regarding model structure are provided in the appendix (pp 3–4).

The posterior distribution (ie, posterior medians and 95% highest posterior density credible intervals [CrIs]) of percentage differences between observed and predicted (2020–22) tuberculosis case notifications, notification rates per 100 000 person-years who are incarcerated, and prison population size during COVID-19 pandemic years are presented by region, subregion, national tuberculosis burden category, prison tuberculosis notification burden (low: 0 to <100 cases per 100 000 person-years, medium: ≥100 to ≤500 cases per 100 000 person-years, and high: >500 cases per 100 000 person-years), and prison overcrowding levels. The cutoffs for prison tuberculosis notification burden were chosen to create an even distribution across groups to maximise the precision of our estimates when stratifying. The primary analysis includes countries that reported data in any of the COVID-19 years (2020, 2021, or 2022). The secondary analysis describes percentage differences between observed and predicted prison tuberculosis case notifications, prison tuberculosis notification rates, and prison population in countries with available data in all COVID-19 years (2020, 2021, and 2022), by subgroup (region, subregion, national tuberculosis burden category, prison tuberculosis notification burden, and prison overcrowding levels). A secondary analysis was done to allow for comparison of the same subset of countries across years.

The STROBE checklist can be found in the appendix (pp 59–61).^28^ Ethical approval for this study was unnecessary as the data are at the aggregate level.

### Role of the funding source

The funders of the study had no role in study design, data collection, data analysis, data interpretation, or writing of the report.

## Results

**Figure 1:**
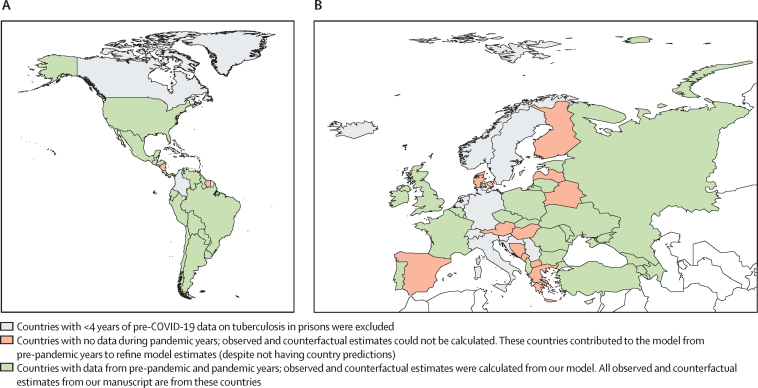
Map of countries included in our analysis from the Pan American Health Organization Region (A) and WHO European region (B) Shapefiles correspond to geographical demarcations of the Americas and Europe, rather than WHO region definitions. Israel is not included in the map but is included in the model. Kazakhstan, Kyrgyzstan, and Tajikistan are not included in the map but are included in the model and analysis.

Of the 98 countries in the WHO European and PAHO regions, 63 (64·3%) had sufficient pre-COVID-19 pandemic data and were used to fit the proposed joint model; 35 countries were excluded due to insufficient historical data (figure 1). Overall, the 35 countries that were excluded were largely similar to the included countries in terms of incarceration level, percentage of incarcerated population that were female, and prison capacity. Excluded countries had lower case notifications and incidence rates than included countries. Characteristics comparing the 63 included countries with the 35 excluded countries are provided in the appendix (p 41). The fitted model was then used to predict outcomes for the 47 countries that reported data in the post-COVID-19 years (2020–22; appendix p 4). Of these 47 countries, 25 were from the WHO European region and 22 were from the PAHO region. The 25 countries from WHO Europe represent 47% of all 53 countries in WHO Europe. The 22 countries from PAHO represent 56% of all 39 countries in PAHO. These 47 countries also contributed pre-COVID-19 pandemic data for the model (figure 1).

Overall, more than a third (18 [38·3%] of 47) of these countries had low (ie, 0–20 cases per 100 000 person-years) tuberculosis burden among the general population; a mean of 4·4% (SD 2·8%) of people incarcerated were female. In terms of crowding in prisons, 38·3% (18 of 47) reported no overcrowding (ie, operated at <100% capacity), 19·1% (9 of 47) had prisons operating at 100–120% capacity, 25·5% (12 of 47) at >120–200% capacity, and 12·8% (6 of 47) at over 200% capacity (table). Country-level pre-COVID-19 trends for tuberculosis notifications, notification rates, and prison population for all countries are available in the appendix (pp 5–40). The 47 countries we present results for represent 42% (4 883 156 of 11 626 562) of the global total number of people who are incarcerated. The distribution of the total number of people who are incarcerated globally by region and a list of all countries and the years of tuberculosis notification data they contributed to the model are provided in the appendix (pp 42–44).

**Table:** Characteristics of the 47 countries in which we estimated the effect of the COVID-19 pandemic

		**Countries (n=47)**
WHO region		
	Americas	22 (46·8%)
	Europe	25 (53·2%)
Subregion		
	Central and North America	12 (25·5%)
	South America	10 (21·3%)
	Eastern Europe	18 (38·3%)
	Western Europe	7 (14·9%)
Sex		
	Female, mean (SD)	4·4 (2·8)
	Male, mean (SD)	95·6 (2·8)
	Missing*	28 (59·6%)
Tuberculosis burden in the general population		
	Low (0–20 cases per 100 000 person-years)	18 (38·3%)
	Medium (>20 to ≤50 cases per 100 000 person-years)	16 (34·0%)
	High (>50 cases per 100 000 person-years)	13 (27·7%)
Tuberculosis burden in prisons		
	Low (0 to <100 cases per 100 000 person-years)	11 (23·4%)
	Medium (≥100 to ≤500 cases per 100 000 person-years)	16 (31·9%)
	High (>500 cases per 100 000 person-years)	21 (44·7%)
Prison overcrowding (% of official capacity), mean (SD)		135·8 (85·1)
	Missing†	2 (4·3%)
Prison overcrowding category (% of official capacity occupied)		
	<100%	18 (38·3%)
	100–120%	9 (19·1%)
	>120–200%	12 (25·5%)
	>200%	6 (12·8%)
	Missing†	2 (4·3%)
Country-years of follow-up pre-COVID-19		388
	2010–14	164
	2015–19	224
	Country-years of follow-up during COVID-19 (2020–22)	132

Data shown are n, n (%), or mean (SD).

* Shows n (%) of countries with missing data on sex. Characteristics based on 2018 data.

† Tajikstan and Ireland do not report any prison capacity data.

**Figure 2:**
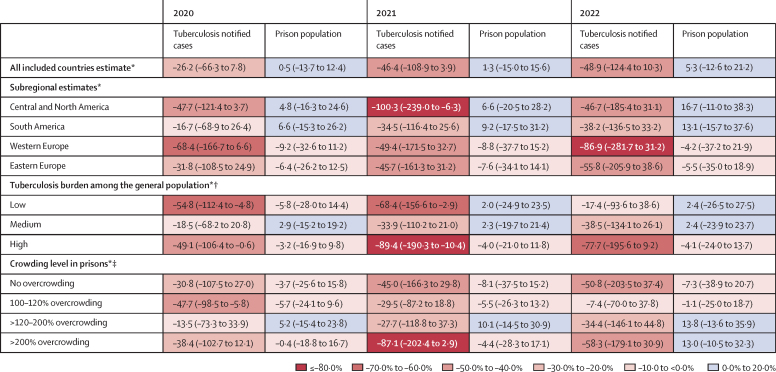
Percentage difference and 95% credible intervals between observed and predicted tuberculosis notified cases in prisons and prison populations, stratified by key subgroups, 2020–22 Percentage differences are calculated by taking the difference between the predicted and observed. The predicted estimate is determined by the joint model estimating tuberculosis notified cases and prison population under a counterfactual scenario in which the COVID-19 pandemic did not occur for 47 countries that reported observed data for these outcomes. This model is based on data from 63 countries (including these 47 countries). More details can be found in the appendix (pp 3–4). Observed values and predicted estimates and 95% credible intervals are reported in the appendix (p 45). Subgroup estimates will not sum up to the global estimate as these are percentage differences between the observed and the predicted. *All 47 countries are included. †Low burden: 0 to 20 cases per 100 000 person-years; medium burden: >20 to ≤50 cases per 100 000 person-years; high burden: >50 cases per 100 000 person-years. ‡45 countries are included, two countries (Tajikistan and Ireland) are excluded as they do not report any prison capacity data.

We calculated percentage differences between observed and predicted tuberculosis case notifications in prisons, notification rates in prisons, and prison populations during the COVID-19 pandemic years. 40 countries reported data in 2020, 42 reported data in 2021, and 43 reported data in 2022. In total, among these countries, there was a decrease of 26·2% (95% CrI –66·3 to 7·8) in the number of tuberculosis cases reported compared with the predicted number of cases in 2020, a 46·4% (–108·9 to 3·9) decrease in 2021, and a 48·9% (–124·4 to 10·3) decrease in 2022 (figure 2). When stratifying by subregion, Central and North America reported a 47·7% (–121·4 to 3·7) decrease in the number of tuberculosis cases reported compared with the predicted number in 2020 and a 100·3% (–239·0 to –6·3) decrease in 2021. Similarly, in countries with a low tuberculosis burden among the general population, there was a decrease in the number of reported cases in prisons compared with the predicted cases of 54·8% (–112·4 to –4·8) in 2020 and 68·4% (–156·6 to –2·9) in 2021. Among countries with a high tuberculosis burden among the general population, there was a 49·1% (–106·4 to –0·6) decrease in the number of reported tuberculosis cases compared with the predicted number of cases in prisons in 2020 and a 89·4% (–190·3 to –10·4) decrease in 2021. There was little recovery noted in our estimates for 2022 when stratifying by key subgroups (figure 2). Observed and predicted estimates are reported in the appendix (p 45).

Both the American and European regions had observed notification rates during the COVID-19 pandemic that were lower than their predicted values. In the Americas, the percentage differences between the observed and predicted notification rates were –8·5% (95% CrI –86·0 to 37·9) in 2020, –33·5% (–165·0 to 35·1) in 2021, and –57·7% (–226·9 to 30·9) in 2022. In Europe, the percentage difference between the observed and predicted notifications rate was –19·4% (–128·8 to 40·6) in 2020, –28·6% (–180·7 to 46·9) in 2021, and –38·6% (–237·9 to 52·1) in 2022 (appendix pp 46–47).

Globally, there were small non-significant increases in the observed prison population compared with the predicted prison population across all 3 years (2020: 0·5% [95% CrI –13·7 to 12·4]; 2021: 1·3% [–15·0 to 15·6]; 2022: 5·3% [–12·6 to 21·2]; figure 2). When stratifying by subregion, Central and North America and South America reported similar small increases across all 3 years. However, western and eastern Europe reported small decreases in the observed prison population compared with the predicted population across all 3 years. When stratifying by burden and crowding, most estimates indicated decreases of 5–10% in 2020 and 2021. However, by 2022, the reported prison population was higher than the predicted population for most subgroups.

**Figure 3:**
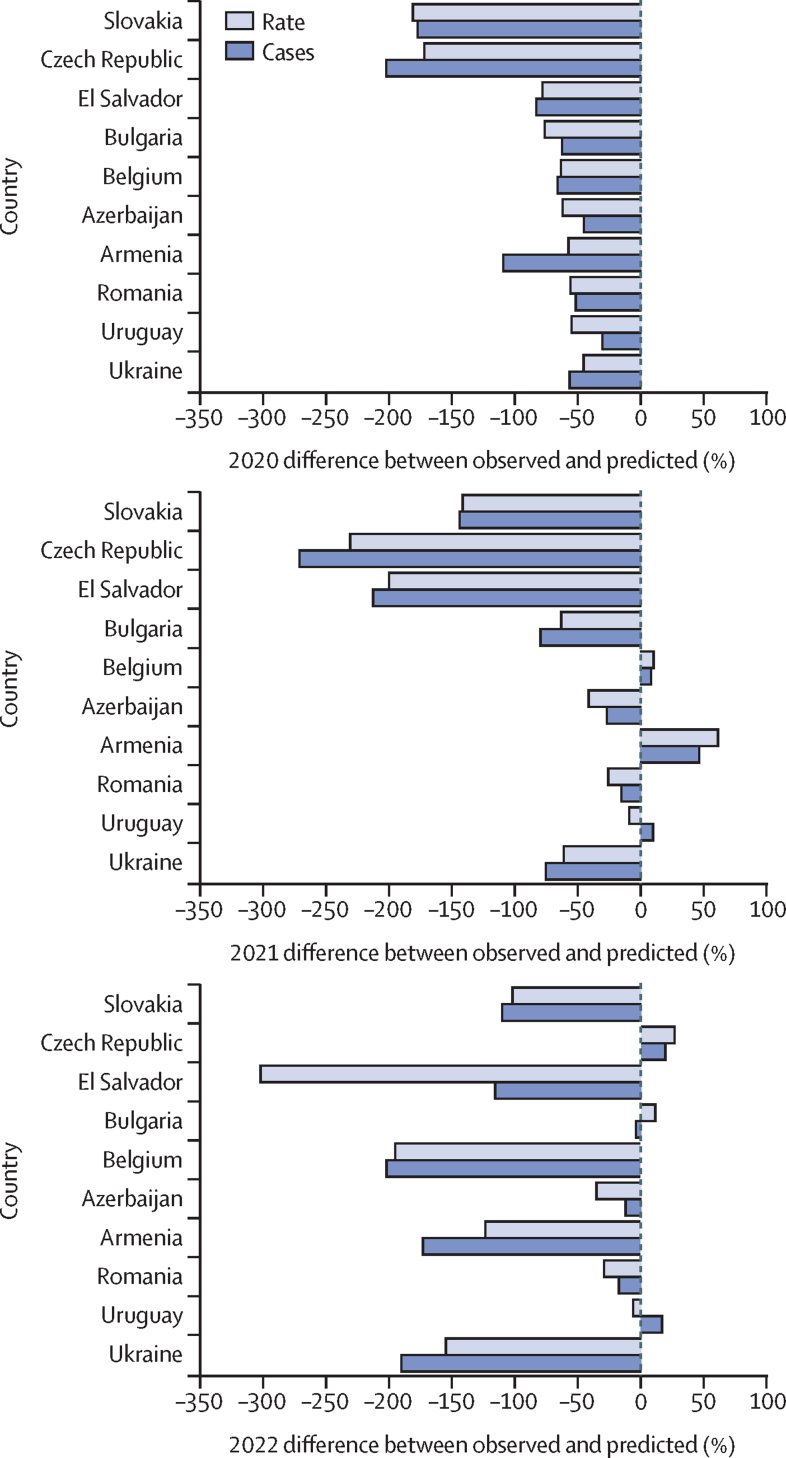
Country-level percentage difference in predicted *vs* observed prison tuberculosis notifications and prison notification rates during COVID-19 pandemic years among the ten countries with the largest decreases

Slovakia, Czech Republic, El Salvador, Bulgaria, Belgium, Azerbaijan, Armenia, Romania, Uruguay, and Ukraine were the ten countries that reported the largest percentage decreases between predicted and observed notification rates in 2020 (figure 3). Trajectories of notifications in 2021 and 2022 in these countries were heterogeneous, with some countries (Czech Republic, El Salvador, Bulgaria, and Ukraine) reporting continuously lower observed values than predicted values, while other countries reported smaller decreases compared with 2020 (Slovakia, Azerbaijan, and Romania). Three of these countries (Armenia, Belgium, and Uruguay) reported higher observed rates than predicted rates in 2021. Heterogeneity of tuberculosis notifications and notification rates by individual countries is further noted when evaluating 2022 trends—countries such as the Czech Republic and Bulgaria reported continual decreases in 2020 and 2021, followed by an increase in the observed versus predicted rate in 2022. In Armenia, the percentage differences between predicted and observed notification rates decreased in 2020, increased in 2021, and then decreased again in 2022. Not all countries that had large decreases in the first year of the pandemic continued to report decreases in 2021 and 2022.

**Figure 4:**
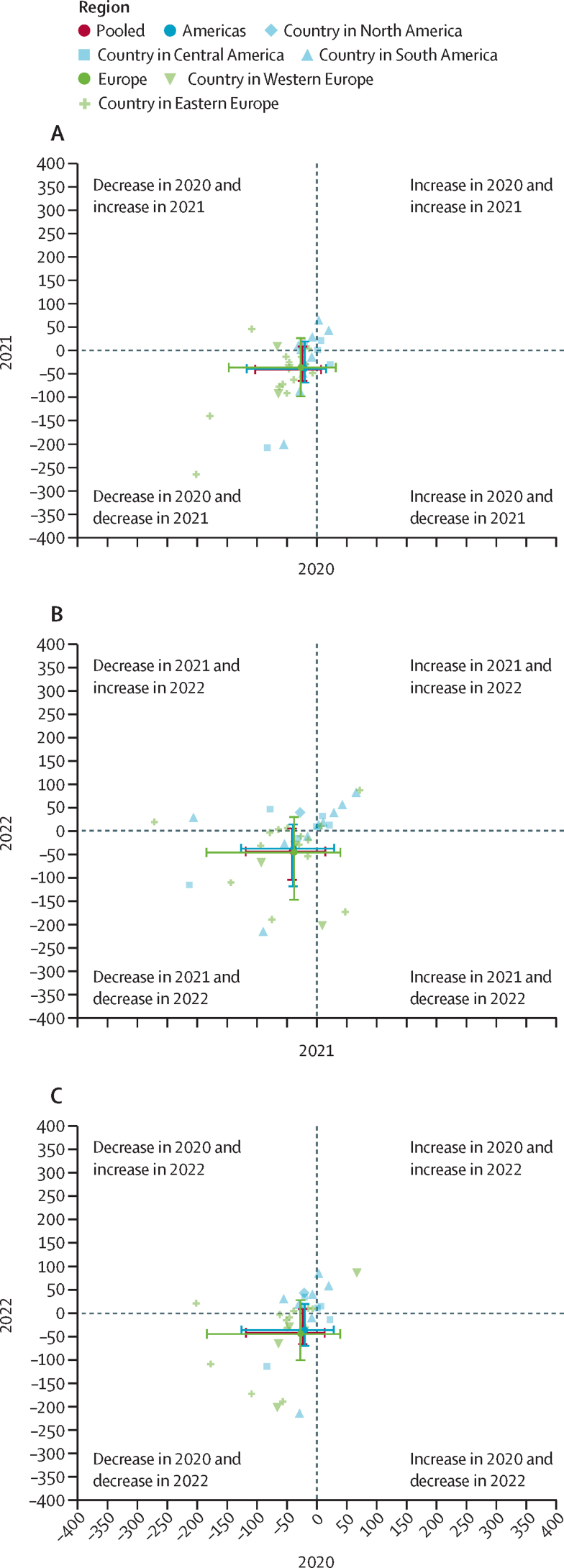
Scatterplot comparing the percentage difference in observed *vs* predicted tuberculosis case notifications among people who are incarcerated stratified by region in 2020 *vs* 2021 (A), 2021 *vs* 2022 (B), and 2020 *vs* 2022 (C) To be included, countries had to have data from 2020 to 2022 (ie, all 3 years the pandemic).

We calculated percentage differences between observed and predicted tuberculosis case notifications in prisons, notification rates in prisons, and prison populations during the COVID-19 pandemic years among countries with available data during all 3 years (2020–22). Findings from this analysis were similar to results from our primary analysis. Most countries reported lower observed versus predicted tuberculosis case notifications and rates over the 3 years, suggesting a consistent reduction in tuberculosis notifications over the COVID-19 pandemic years across all stratification groups (figure 4; appendix pp 49–58). Most countries that reported decreases in the observed versus the predicted tuberculosis notifications in 2020 continued to report decreases in 2021 and 2022 (figure 4). We note that there are some countries that do not follow this trend and are outliers. For example, Czech Republic, Armenia, and Slovakia had percentage differences above 100% in 2020–22. In the Americas, the prison population steadily increased for all 3 years (South: 6·4% [2020], 9·3% [2021], and 13·1% [2022]; Central and North: 5·1% [2020], 6·6% [2021], and 16·9% [2022]). In Europe, there were small decreases in 2020 (eastern: –6·3% and western: –9·4%), 2021 (eastern: –8·2% and western: –9·7%), and 2022 (eastern: –5·6% and western: –4·2%; appendix p 48).

## Discussion

Although there is widespread acknowledgment that the COVID-19 pandemic had substantial detrimental effects on tuberculosis control globally,^16^, ^17^, ^18^, ^19^, ^20^, ^21^, ^22^, ^23^, ^24^, ^25^ there is a concern that these effects might be concentrated among vulnerable individuals at high risk. Using a hierarchical Bayesian time-series model, we examined tuberculosis notification trends among people who were incarcerated from 2010 to 2022 in 47 countries (representing almost 5 million people in prisons annually) in Europe and the Americas. During the COVID-19 pandemic years, we observed considerably lower tuberculosis notifications (compared with expected trends) from prisons. These results were generally consistent across all years of the COVID-19 pandemic, as the reduction in tuberculosis notification cases was still evident in 2021 and 2022. The observed data over the years show a continual decline in tuberculosis notifications among people who are incarcerated; this is distinct from tuberculosis notification trends in the general population where there was a substantial decline in 2020 (and in 2021 in many countries) followed by a general increase in subsequent years.^1^, ^17^ These trends were also mostly consistent across both Europe and the Americas, but larger decreases were seen in Central and North America and in low-burden and high-burden settings. Our findings of lower observed notifications versus expected notifications were consistent with another study that evaluated the changes in childhood tuberculosis notifications across the globe in 2020.^29^ Incarceration levels were largely similar to predicted levels from 2020 to 2022. The small increases in incarceration that were observed probably do not explain the decrease in tuberculosis notification cases observed during this same time period. If changes in tuberculosis notifications were due to decarceration during the pandemic, we would expect to see much greater reductions in incarceration levels than seen in our study.

Our results across 47 countries in Europe and the Americas show substantial reductions (around 20–70%) in tuberculosis notifications from prisons in 2020–22 compared with pre-pandemic years. In our analysis, the ratio of observed versus expected tuberculosis notifications was consistently high throughout the study time period, which suggests that the effect of the COVID-19 pandemic on prisons might not be improving (although alternative explanations of under-notification, such as true decreases in incidence, are discussed further), and urgent attention and further resources are needed to aid tuberculosis control in prisons more broadly and in preparation for future pandemics. These results are especially concerning as prisons have been shown to act as amplifiers of *M tuberculosis* transmission outside of prisons due to substantial inflow and outflow between prisons and the broader community.^30^ Further data are needed to understand reasons for the persistence of these trends. A recent study using Peruvian National Tuberculosis Program data found that tuberculosis notifications dropped among incarcerated populations by 17·7% (95% CI –17·5 to –17·9) in the first week of the COVID-19 pandemic (compared with a counterfactual scenario).^31^ However, to date, little research has been done on this specific vulnerable population, which is a barrier to prioritising incarcerated populations in the overall tuberculosis response. Furthermore, although we report that notifications have broadly decreased across countries for all 3 years, results at the country level might be highly heterogeneous. Future work should be done at the country level to better understand the differential effect of the COVID-19 pandemic within countries.

The decreased number of tuberculosis notifications in prisons compared with expected trends suggests lower tuberculosis case detection in prisons in these countries. Before the COVID-19 pandemic, the estimated global tuberculosis case detection ratio for prisons was 53% (95 CrI 42–64) in 2019,^6^ a low number compared with other groups at high risk. Therefore, further decreases in tuberculosis case detection in prisons is concerning as it could suggest missed diagnoses, resulting in individuals not being treated during the pandemic. Untreated tuberculosis places the individual at greater risk of developing drug-resistant tuberculosis and mortality and placing others (such as health staff, community members upon the individual's release, and other people who are incarcerated) at increased risk of infection. Understanding tuberculosis notifications in the context of these data is complicated given that notifications are an under-representation of true incidence. Although our results suggest decarceration probably does not explain the lower-than-expected observed notifications in our results, there might have been reductions in *M tuberculosis* transmission from COVID-19 pandemic-related interventions (eg, masking, quarantine, and physical distancing). These interventions have been variably implemented in prisons globally and are probably not present in later years of our study time period.^20^, ^21^, ^22^, ^23^, ^24^, ^25^ When used, these interventions were often implemented poorly or inconsistently; previous reports show the lack of prioritisation of people who are incarcerated in national COVID-19 pandemic responses.^13^, ^21^, ^22^, ^23^, ^24^, ^25^ Mechanisms explaining these results should be further explored within each country, given the observed heterogeneity. Furthermore, people who are incarcerated typically come from communities with a high background tuberculosis burden, and these communities were disproportionately affected by the COVID-19 pandemic.^6^, ^7^, ^8^, ^9^ Implementing these interventions is further complicated by the fact that in many countries, prison health is under the jurisdiction of the Ministry of Justice or Defence, rather than the Ministry of Health.^32^ Therefore, National Tuberculosis Programs and Ministries of Health are often limited in their ability to implement and enforce tuberculosis screening and treatment guidelines within the carceral setting. Greater prioritisation needs to be given to incarcerated populations, as they are often at higher risk of infection and adverse health outcomes than the general population, which also places other individuals (eg, prison staff and post-community release network) at risk.^33^

These results should be interpreted within the context of several limitations. First, tuberculosis-related data among incarcerated populations are generally sparse and might lead to reduced precision of our predictions. Completeness and quality of notification and incarceration data are variable across country and study years. Notification data are reported by experts in each country (eg, National Tuberculosis Program Officials) and incarceration data are collected from various sources (eg, each country's Ministry of Justice and researchers). Second, the data used in this analysis are annual data reported at a country level. Annual data might bias our results if the duration of incarceration is highly variable. Data collected at the monthly or weekly level for each country would lead to increased precision, minimise bias due to duration of incarceration, and improve understanding of underlying trends. Furthermore, our results are presented at the aggregate level, and we do not report individual country-level estimates due to limited power. Third, we were unable to provide estimates stratified by or adjusted for prison settings (eg, open prisons *vs* semi-open prisons), demographics (eg, sex, age, groups at high risk, and race and ethnicity), or incarceration status (eg, remand, detainee, or convicted) due to the limited detail provided within the available data. Accounting for demographic factors such as race and ethnicity and sex would be important to analyse in future studies. Fourth, we were unable to understand mechanisms behind changes in tuberculosis notifications. For example, if diagnosis happened at later stages of disease, this would suggest that missed or delayed diagnosis would be the primary reason for the observed decreases in our results. Future work should aim to elucidate these mechanisms to better inform guidelines for screening and treatment. Fifth, we were only able to include region as a predictor in our model because other predictors were affected by the pandemic. Including predictors in our regression model that were influenced by the pandemic would lead to biased counterfactual estimates. Finally, our results might not be generalisable to countries outside these two regions or to countries within the regions but not included in this analysis. The countries included in our analysis tended to have a higher tuberculosis burden and larger prison populations than countries in the PAHO and WHO European region that were excluded.

Identifying vulnerable populations at high risk who were disproportionately affected by the pandemic is a major tuberculosis control priority. Our analysis suggests that the effects of the COVID-19 pandemic on tuberculosis control in prisons were substantial, with a reduction of 20–70% in tuberculosis notifications, compared with counterfactual estimates. Our results remained lower in 2021 and 2022, suggesting that recovery in tuberculosis care in prisons was slow and compensatory resources were insufficient. Time periods where there is substantial health-care hardship (such as pandemics) are important to prepare for, including within incarcerated settings. Preventing further health inequities in the carceral setting and ensuring necessary resources (eg, funding, staffing, testing, and treatment) are provided and sufficient is crucial before and during these periods. Increased collaboration between Ministries of Health and Ministries of Justice will be needed, as highlighted in the WHO European region report on prisons and COVID-19, to address these issues and improve tuberculosis control within the carceral setting—especially as this analysis could not evaluate the effect of prison-specific factors (eg, open air *vs* not).^34^

### Contributors

### Data sharing

Data collected from the World Prison Brief are publicly available. Data collected from the European Centers for Disease Control and Prevention and Pan American Health Organization can be shared upon reasonable request from the corresponding author.

## Declaration of interests

We declare no competing interests. PA is a staff member of the Pan American Health Organization. The author alone is responsible for the views expressed in this publication, and they do not necessarily represent the decisions or policies of the Pan American Health Organization.
