# Supplementary material for: Changes in incarceration and tuberculosis notifications from prisons during the COVID-19 pandemic in Europe and the Americas: a time-series analysis of national surveillance data
Source: Lancet Public Health. 2025 Mar 31;10(4):e285–94. doi: 10.1016/S2468-2667(24)00325-6 (PMC11962358; doi:10.1016/S2468-2667(24)00325-6)
Supplement: Supplementary appendix [file mmc1.pdf]

THE LANCET
Public Health

Supplementary appendix

This appendix formed part of the original submission and has been peer reviewed. We post it as supplied by the authors.

Supplement to: Zheng A, Faust L, Harries AD, et al. Changes in incarceration and tuberculosis notifications from prisons during the COVID-19 pandemic in Europe and the Americas: a time-series analysis of national surveillance data. *Lancet Public Health* 2025; **10:** e285–94.

Contents

Supplemental Section 1: Model Details 3

Supplemental Section 1: Figure 1. Flow diagram of countries included in the manuscript and analysis.......... 4

Supplement Section 2: Country-Level Trends in Observed and Predicted Prison Tuberculosis Cases, Prison Tuberculosis Rates, and Prison populations 5

TB case notification rates: American Region 5

TB case notification rates: European Region 10

TB case notifications: American Region 17

TB case notifications: European Region 22

Prison population: American Region 29

Note: observed values for prison population are used in the model, unless missing (in which case a value is imputed) 29

Prison population: European Region 34

Note: observed values for prison population are used in the model, unless missing (in which case a value is imputed) 34

Supplement Section 3: Outcomes 41

Supplemental Table S1. Characteristics of countries included vs. not included to fit the proposed joint model 41

Supplemental Table S2. Total number of people who are incarcerated by region in 2018 42

Supplemental Table S3. Reporting of national tuberculosis notification data among people who are incarcerated by country and years of data. 43

Supplemental Table 4. Observed, Predicted and 95% credible intervals for tuberculosis notified case in prisons, and prison population, stratified by key subgroups, from 2020 - 2022 45

Supplemental Table S5. Percent Difference between the observed and predicted tuberculosis case rate per 100, 000 stratified by key subgroups 46

Supplemental Table S6. Percent Difference between the observed and predicted tuberculosis notifications, notification rate, and prison population stratified by WHO Region and tuberculosis burden in prisons 47

Supplemental Figure S1. Scatterplot comparing the percent difference in observed vs. predicted prison population stratified by region in A) 2020 vs. 2021, B) 2021 vs. 2022, and C) 2020 vs. 2022. To be included countries had to have data from 2020-2022, i.e., all three years the pandemic. 48

Supplemental Figure S2. Scatterplot comparing the percent difference in observed vs. predicted tuberculosis case notifications stratified by tuberculosis burden among the general population in A) 2020 vs. 2021, B) 2021 vs. 2022, and C) 2020 vs. 2022. To be included countries had to have data from 2020-2022, i.e., all three years of the pandemic. 49

Supplemental Figure S3. Scatterplot comparing the percent difference in observed vs. predicted tuberculosis case notifications stratified by prison crowding in A) 2020 vs. 2021, B) 2021 vs. 2022, and

C) 2020 vs. 2022. To be included countries had to have data from 2020-2022, i.e., all three years of the pandemic. ... 50

Supplemental Figure S4. Scatterplot comparing the percent difference in observed vs. predicted tuberculosis case notifications stratified by tuberculosis notification rate among the prison population in A) 2020 vs. 2021, B) 2021 vs. 2022, and C) 2020 vs. 2022. To be included countries had to have data from 2020-2022, i.e., all three years of the pandemic. ... 51

Supplemental Figure S5. Scatterplot comparing the percent difference in observed vs. predicted tuberculosis case notification rate stratified by tuberculosis burden among the general population in A) 2020 vs. 2021, B) 2021 vs. 2022, and C) 2020 vs. 2022. To be included countries had to have data from 2020-2022, i.e., all three years of the pandemic. ... 52

Supplemental Figure S6. Scatterplot comparing the percent difference in observed vs. predicted tuberculosis case notification rate stratified by prison crowding in A) 2020 vs. 2021, B) 2021 vs. 2022, and C) 2020 vs. 2022. To be included countries had to have data from 2020-2022, i.e., all three years of the pandemic. ... 53

Supplemental Figure S7. Scatterplot comparing the percent difference in observed vs. predicted tuberculosis case notification rate stratified by tuberculosis notification rate among the prison population in A) 2020 vs. 2021, B) 2021 vs. 2022, and C) 2020 vs. 2022. To be included countries had to have data from 2020-2022, i.e., all three years of the pandemic. ... 54

Supplemental Figure S8. Scatterplot comparing the percent difference in observed vs. predicted prison population stratified by tuberculosis burden among the general population in A) 2020 vs. 2021, B) 2021 vs. 2022, and C) 2020 vs. 2022. To be included countries had to have data from 2020-2022, i.e., all three years of the pandemic. ... 55

Supplemental Figure S9. Scatterplot comparing the percent difference in observed vs. predicted prison population stratified by prison crowding in A) 2020 vs. 2021, B) 2021 vs. 2022, and C) 2020 vs. 2022. To be included countries had to have data from 2020-2022, i.e., all three years of the pandemic. ... 56

Supplemental Figure S10. Scatterplot comparing the percent difference in observed vs. predicted tuberculosis notification per 100,000 people who are incarcerated stratified by region in A) 2020 vs. 2021, B) 2021 vs. 2022, and C) 2020 vs. 2022. To be included countries had to have data from 2020-2022, i.e., all three years the pandemic. ... 57

Supplemental Figure S11. Scatterplot comparing the percent difference in observed vs. predicted prison population stratified by tuberculosis notification rate among the prison population in A) 2020 vs. 2021, B) 2021 vs. 2022, and C) 2020 vs. 2022. To be included countries had to have data from 2020-2022, i.e., all three years of the pandemic. ... 58

Supplemental Section 4: STROBE Statement Checklist of items that should be included in reports of observational studies ... 59

Supplemental Section 1: Model Details

Statistical Model for Case Counts:

$$Y_{it}|r,p_{it} \sim \text{Negative Binomial}(r,p_{it}), p_{it} = \frac{r}{r+\lambda_{it}};\ i=1,\dots,n; t=1,\dots,m;$$

$$\ln(\lambda_{it}) = \ln(O_{it}) + \beta_{0i} + \beta_{1i}t + \gamma \mathrm{x}_i + \alpha_{it}$$

Statistical Model for Prison Population Counts:

$$O_{it}|s,\pi_{it} \sim \text{ Negative Binomial}(s,\pi_{it}), \pi_{it} = \frac{s}{s+\delta_{it}};\ i=1,\dots,n; t=1,\dots,m;$$

$$\ln(\delta_{it}) = \mu_i + \eta_{it}$$

Random Effect Distributions:

- $\begin{pmatrix}\beta_{0i}\\ \beta_{1i}\end{pmatrix} \sim \text{MVN}\left(\begin{pmatrix}\beta_0\\ \beta_1\end{pmatrix}, \Omega\right);\ i=1,\dots,n$: Country-specific intercepts and slopes
- $\mu_i \sim \text{N}(\mu,\tau^2);\ i=1,\dots,n$: Country-specific intercept
- $\boldsymbol{\alpha}_i|\rho_\alpha,\sigma_\alpha^2 \sim \text{MVN}\left(0,\frac{\sigma_\alpha^2}{1-\rho_\alpha^2}\Sigma(\rho_\alpha)\right), \Sigma(\rho_\alpha)_{ij} = \rho_\alpha^{|i-j|};\ i=1,\dots,n$
  - $\boldsymbol{\alpha}_i^{\mathrm{T}} = (\alpha_{i1},\dots,\alpha_{im})$
  - Country-specific autocorrelated random effects
- $\boldsymbol{\eta}_i|\rho_\eta,\sigma_\eta^2 \sim \text{MVN}\left(0,\frac{\sigma_\eta^2}{1-\rho_\eta^2}\Sigma(\rho_\eta)\right), \Sigma(\rho_\eta)_{ij} = \rho_\eta^{|i-j|};\ i=1,\dots,n$
  - $\boldsymbol{\eta}_i^{\mathrm{T}} = (\eta_{i1},\dots,\eta_{im})$
  - Country-specific autocorrelated random effects

Prior Distributions:

- $r,s \sim \text{Gamma}(0.10, 0.10)$: Overdisperson parameters
- $\beta_0,\beta_1,\mu,\gamma \sim \text{N}(0,100^2)$: Regression parameters
- $\rho_\alpha,\rho_\eta \sim \text{Uniform}(0,1)$: Autocorrelation parameters
- $\sigma_\alpha^2,\sigma_\eta^2 \sim \text{Inverse Gamma}(0.01, 0.01)$: Variance parameters for random effects
- $\Omega^{-1} \sim \text{Wishart}(I_2, 3)$: Cross-covariance matrix for country-specific intercepts and slopes
- $\tau^2 \sim \text{Inverse Gamma}(0.01, 0.01)$: Variance parameter for country specific intercept

Additional Information:

- Y_{it}: Tuberculosis case counts from country i in year t (i.e., a dependent variable)
  - n total countries; m pre-COVID-19 years of data
  - λ_{it}: Expected case counts
- x_i: Region indicator variable (Europe/Americas; i.e., an independent variable)

- O_{it}: Prisoner population from country i in year t (i.e., a dependent variable)
    - δ_{it}: Expected prisoner population total

Supplemental Section 1: Figure 1. Flow diagram of countries included in the manuscript and analysis

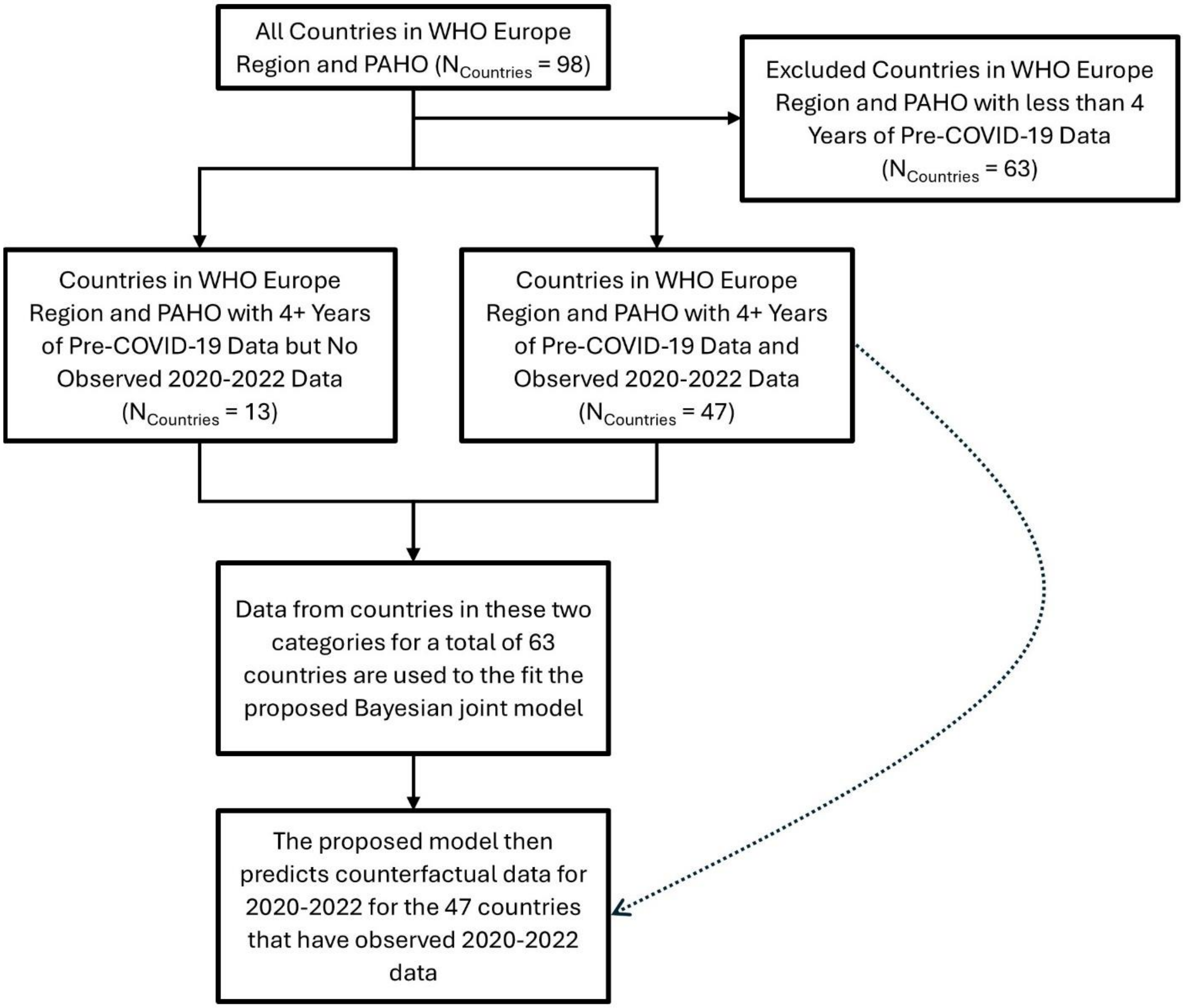

Supplement Section 2: Country-Level Trends in Observed and Predicted Prison Tuberculosis Cases, Prison Tuberculosis Rates, and Prison populations

The figures below show pre-COVID-19 trends in observed and prison TB notifications, notification rates (notified cases per 100,000 people incarcerated) and prison population, for the 63 countries included in the model.

TB case notification rates: American Region

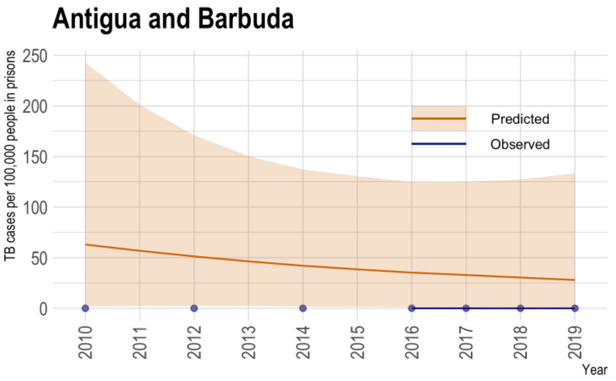

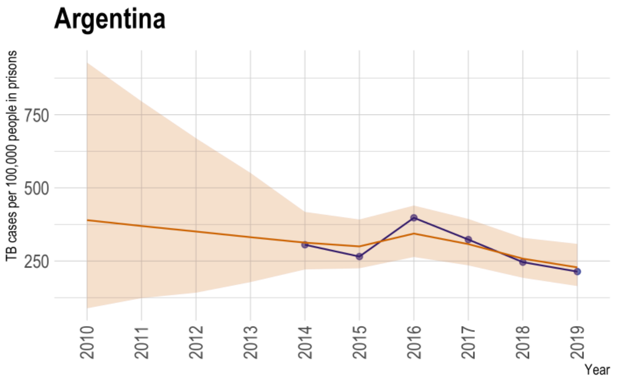

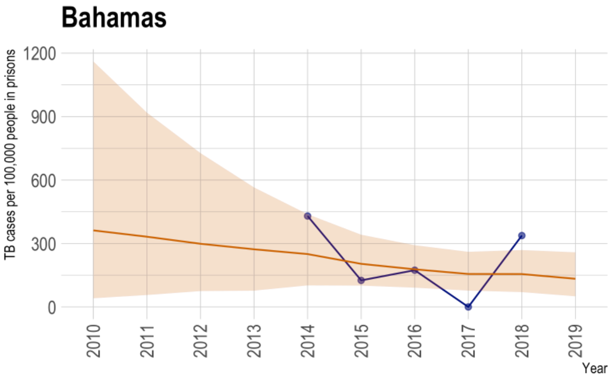

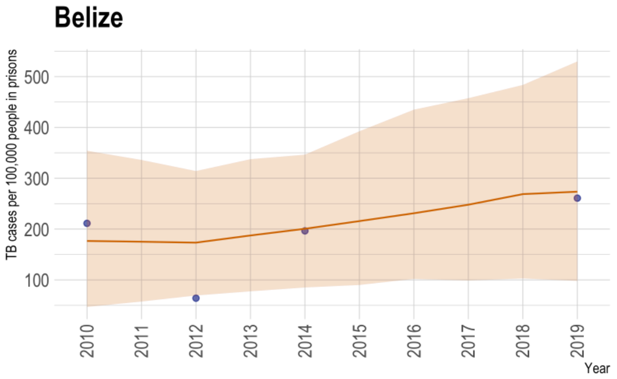

Bolivia

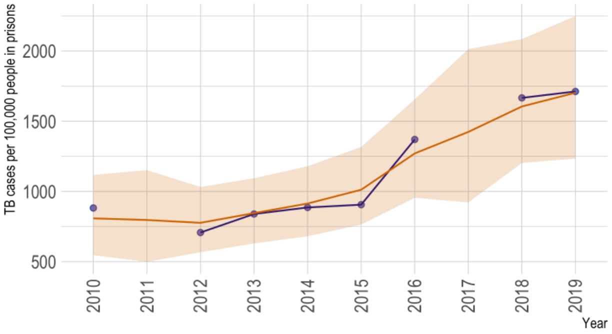

Brazil

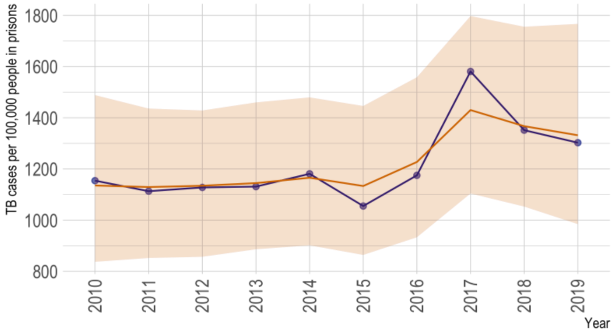

Chile

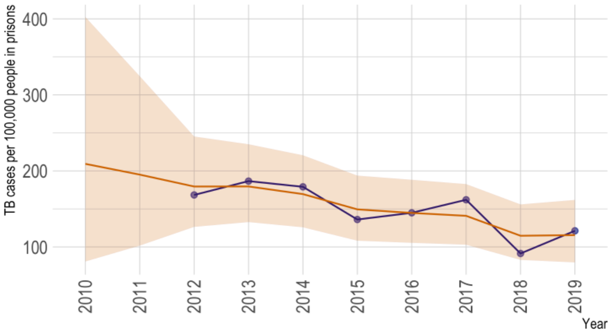

Costa Rica

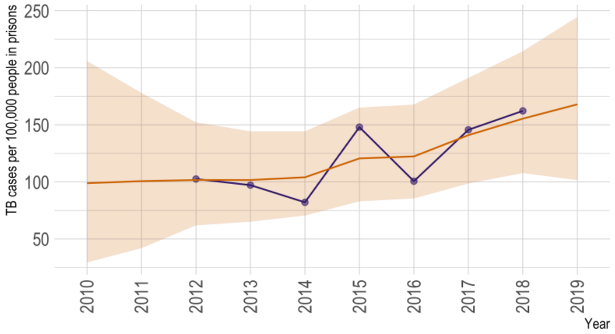

Dominican Republic

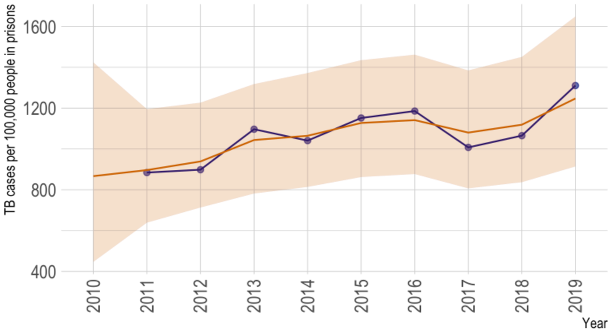

Ecuador

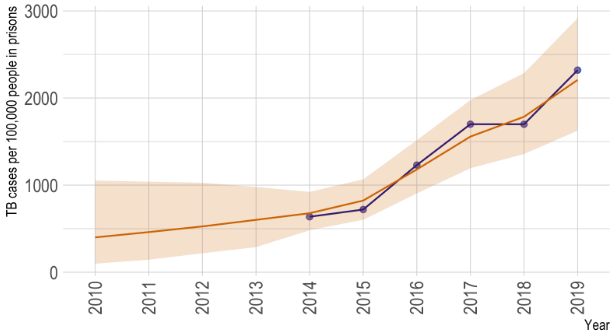

El Salvador

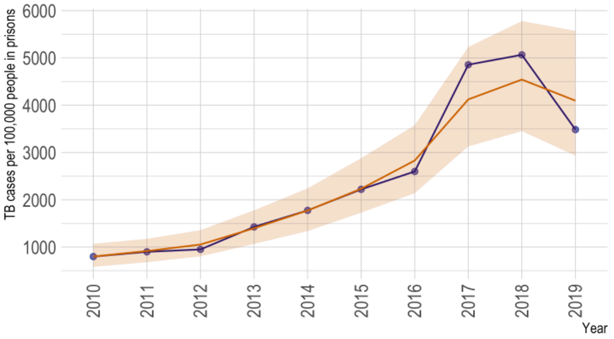

Guatemala

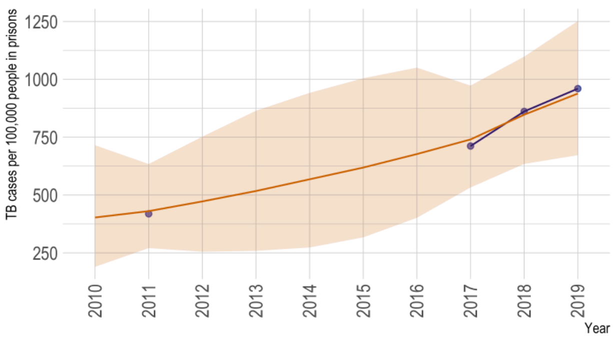

Guyana

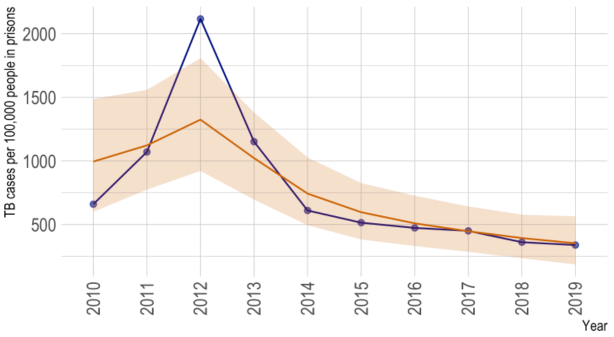

Haiti

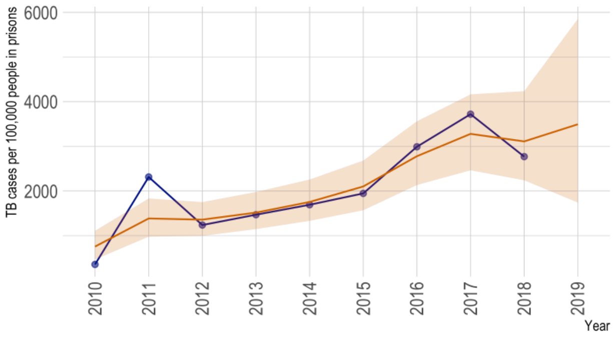

Honduras

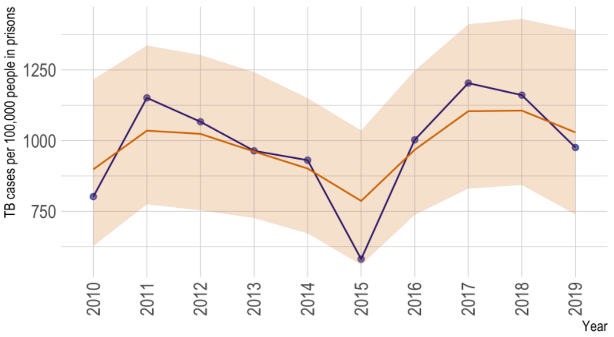

Jamaica

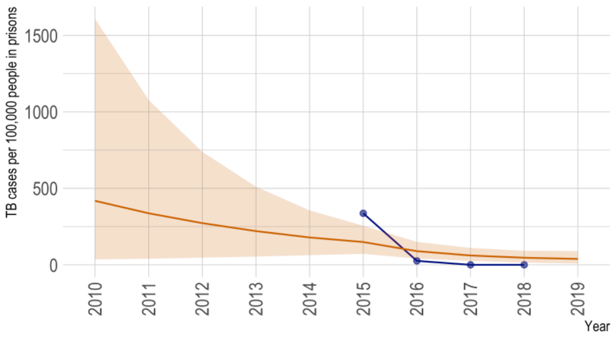

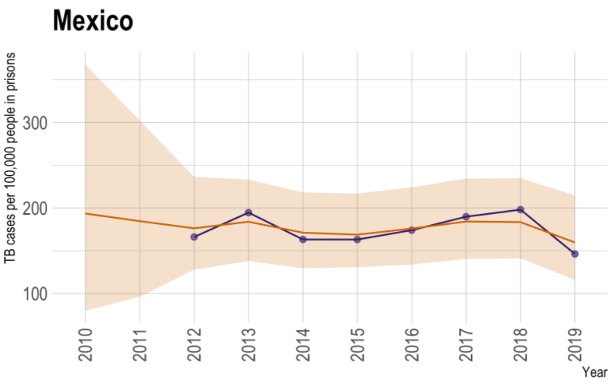
Mexico
TB cases per 100,000 people in prisons
100
200
300
2010
2011
2012
2013
2014
2015
2016
2017
2018
2019
Year

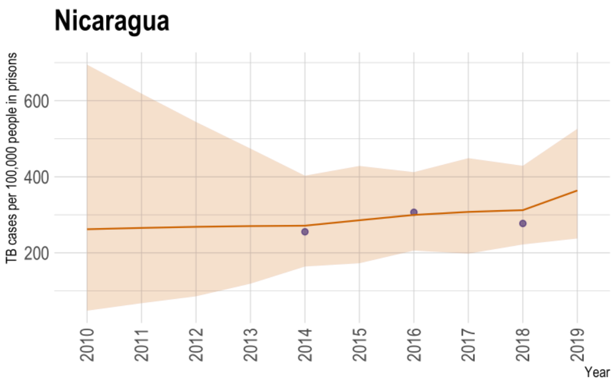
Nicaragua
TB cases per 100,000 people in prisons
200
400
600
2010
2011
2012
2013
2014
2015
2016
2017
2018
2019
Year

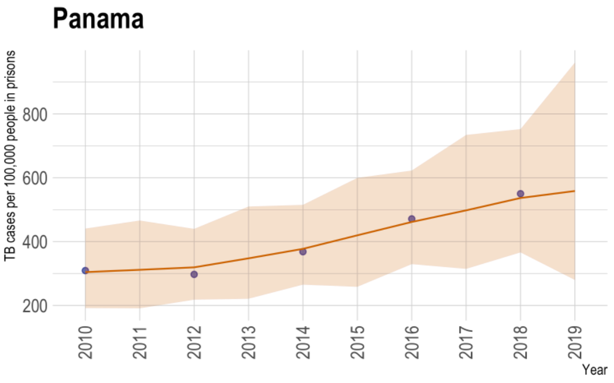
Panama
TB cases per 100,000 people in prisons
200
400
600
800
2010
2011
2012
2013
2014
2015
2016
2017
2018
2019
Year

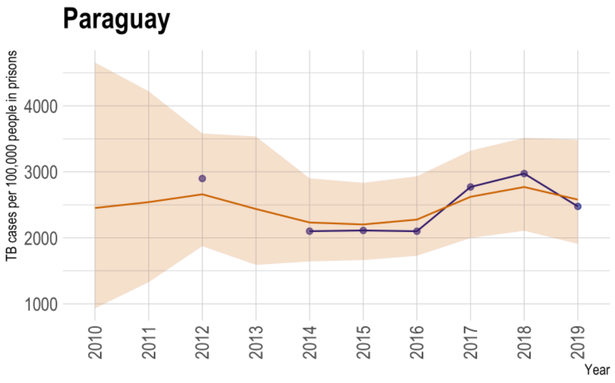
Paraguay
TB cases per 100,000 people in prisons
1000
2000
3000
4000
2010
2011
2012
2013
2014
2015
2016
2017
2018
2019
Year

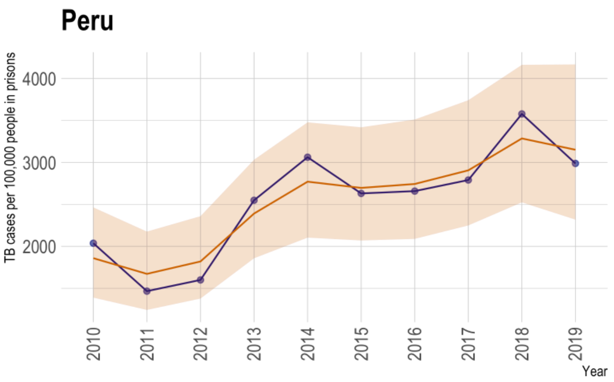
Peru
TB cases per 100,000 people in prisons
2000
3000
4000
2010
2011
2012
2013
2014
2015
2016
2017
2018
2019
Year

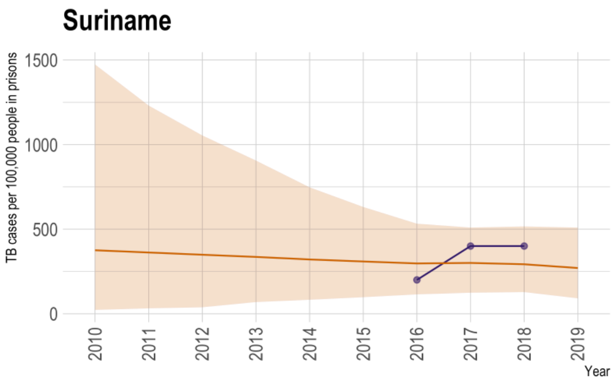
Suriname
TB cases per 100,000 people in prisons
0
500
1000
1500
2010
2011
2012
2013
2014
2015
2016
2017
2018
2019
Year

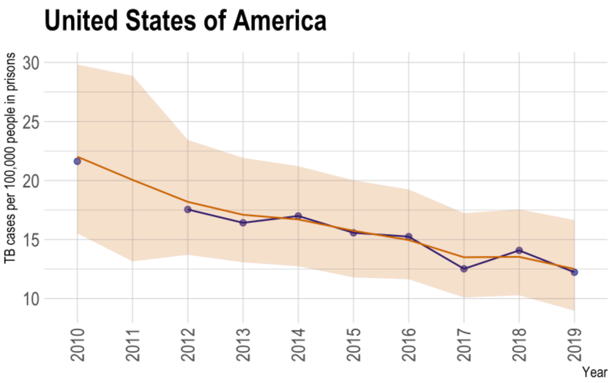
United States of America
TB cases per 100,000 people in prisons
30
25
20
15
10
2010
2011
2012
2013
2014
2015
2016
2017
2018
2019
Year

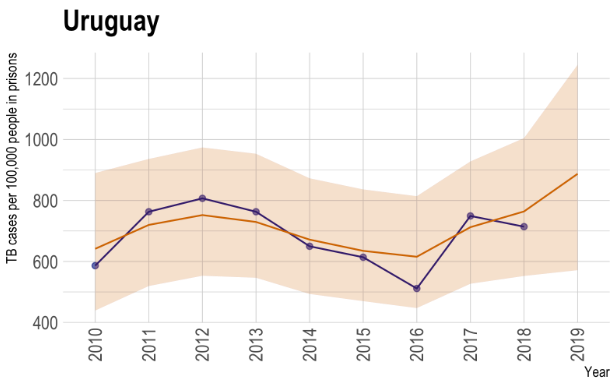
Uruguay
TB cases per 100,000 people in prisons
1200
1000
800
600
400
2010
2011
2012
2013
2014
2015
2016
2017
2018
2019
Year

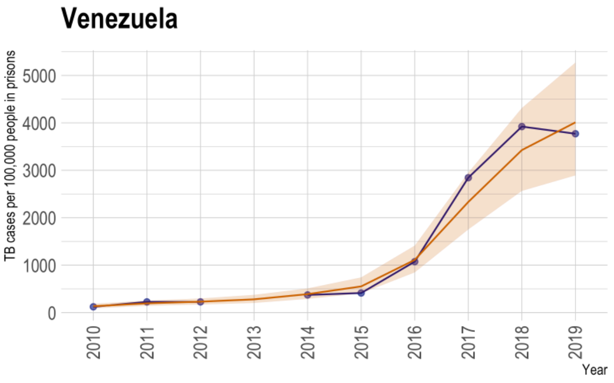
Venezuela
TB cases per 100,000 people in prisons
5000
4000
3000
2000
1000
0
2010
2011
2012
2013
2014
2015
2016
2017
2018
2019
Year

TB case notification rates: European Region

Albania

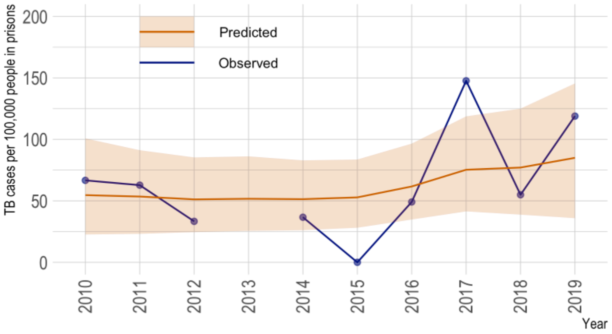

Andorra

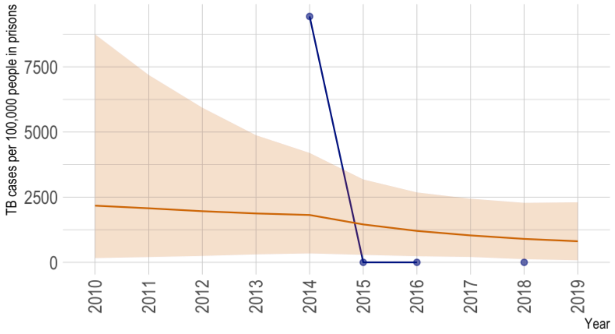

Armenia

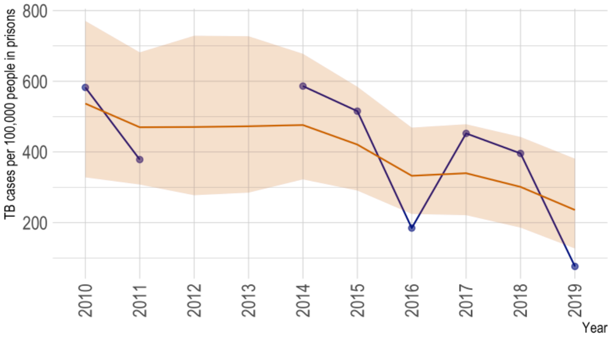

Austria

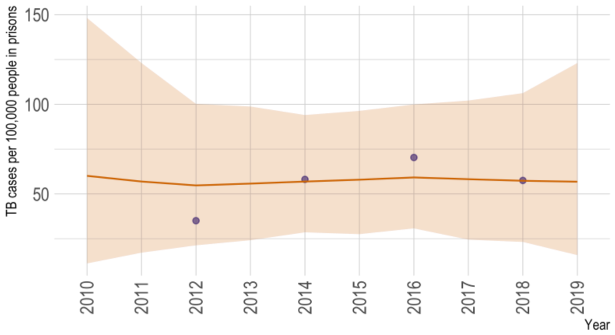

Azerbaijan

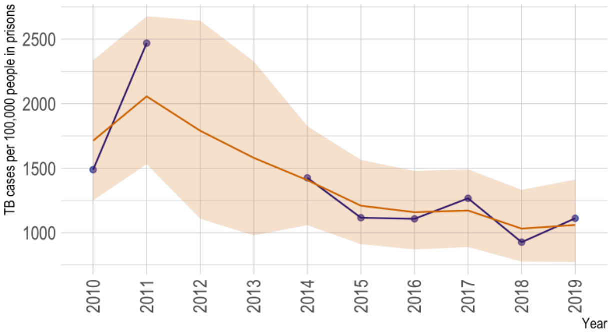

Belarus

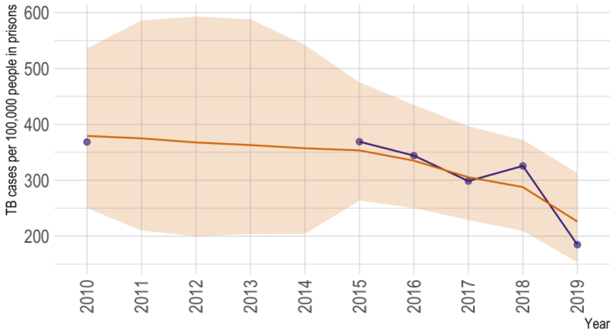

Belgium

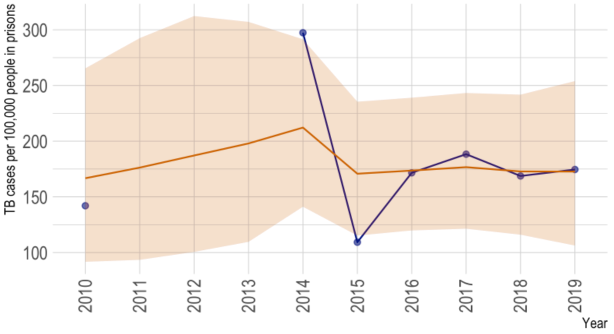

Bosnia and Herzegovina

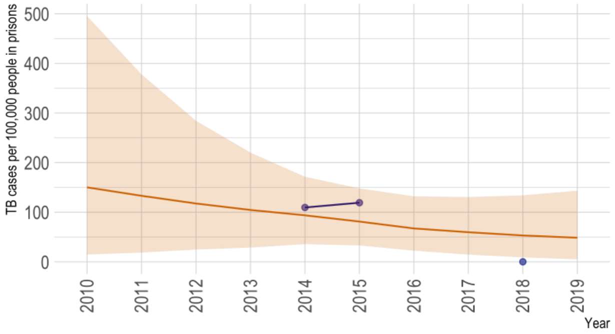

Bulgaria

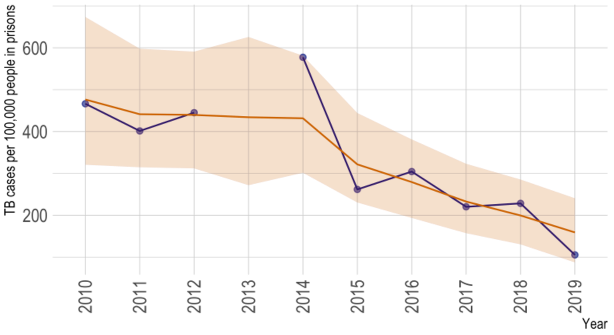

Czech Republic

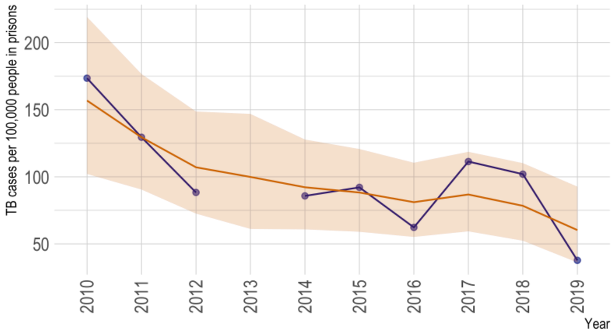

Denmark

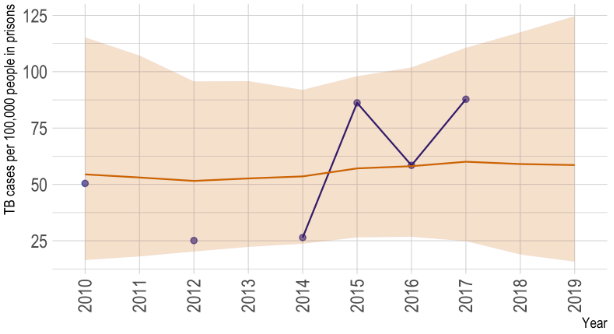

Estonia

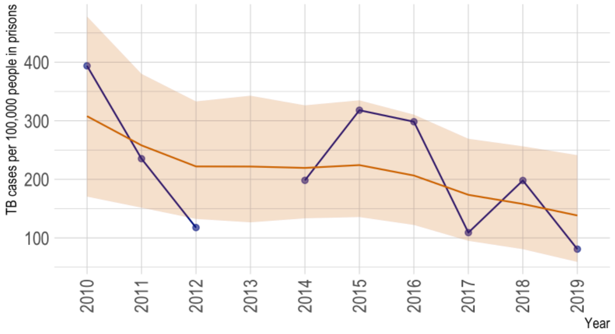

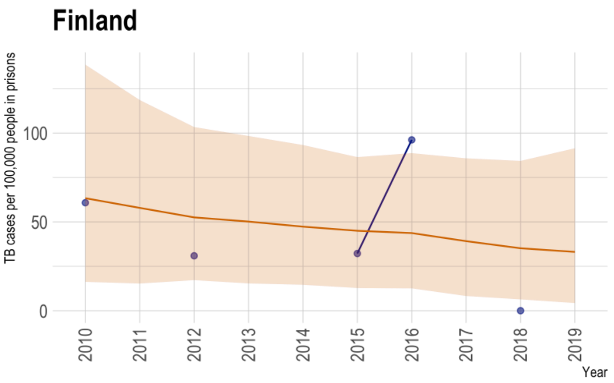
Finland
TB cases per 100,000 people in prisons
0
50
100
2010
2011
2012
2013
2014
2015
2016
2017
2018
2019
Year

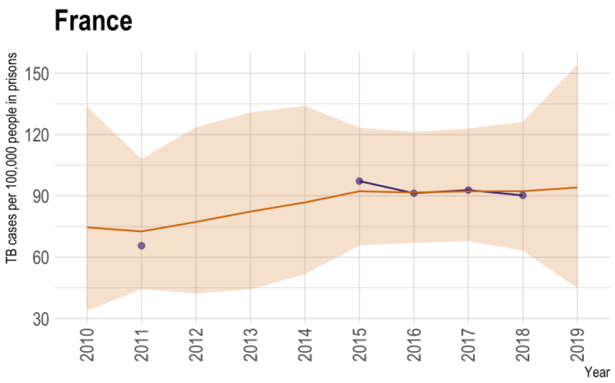
France
TB cases per 100,000 people in prisons
30
60
90
120
150
2010
2011
2012
2013
2014
2015
2016
2017
2018
2019
Year

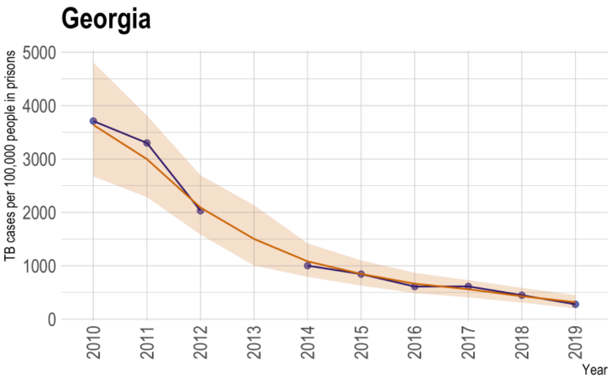
Georgia
TB cases per 100,000 people in prisons
0
1000
2000
3000
4000
5000
2010
2011
2012
2013
2014
2015
2016
2017
2018
2019
Year

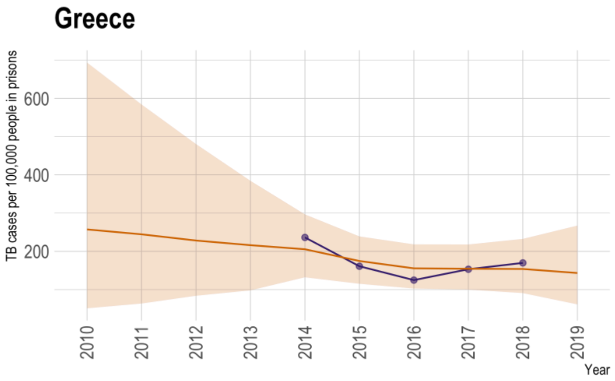
Greece
TB cases per 100,000 people in prisons
200
400
600
2010
2011
2012
2013
2014
2015
2016
2017
2018
2019
Year

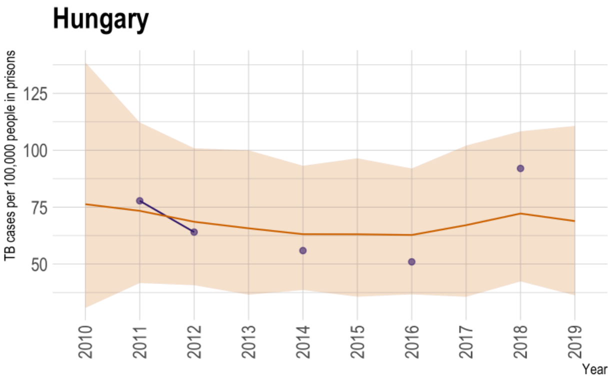
Hungary
TB cases per 100,000 people in prisons
50
75
100
125
2010
2011
2012
2013
2014
2015
2016
2017
2018
2019
Year

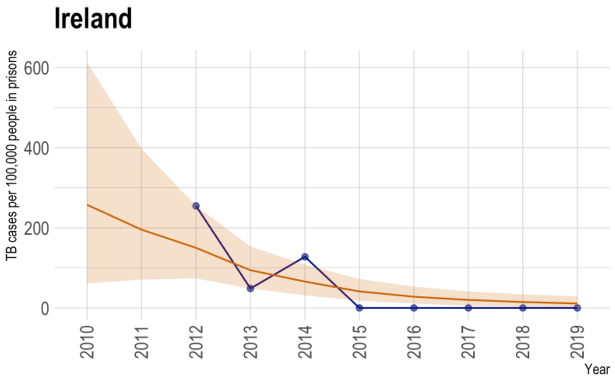
Ireland
TB cases per 100,000 people in prisons
0
200
400
600
2010
2011
2012
2013
2014
2015
2016
2017
2018
2019
Year

Israel

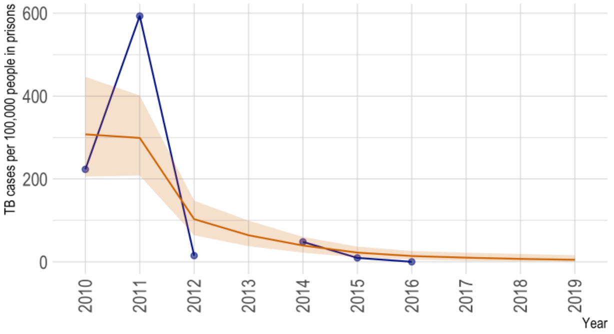

Kazakhstan

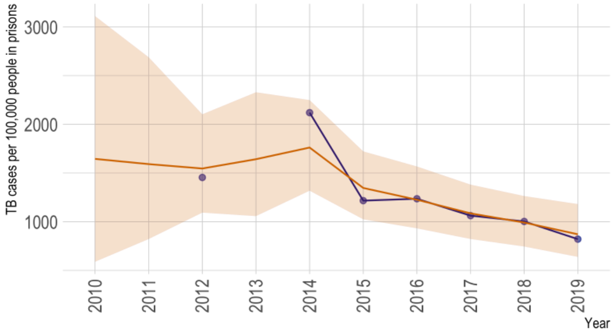

Kyrgyzstan

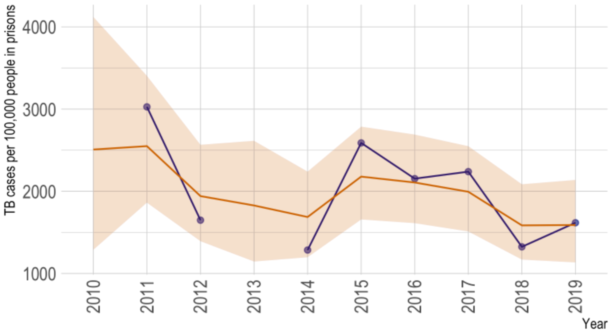

Latvia

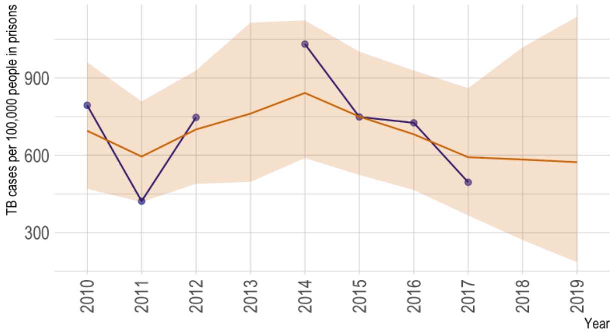

Lithuania

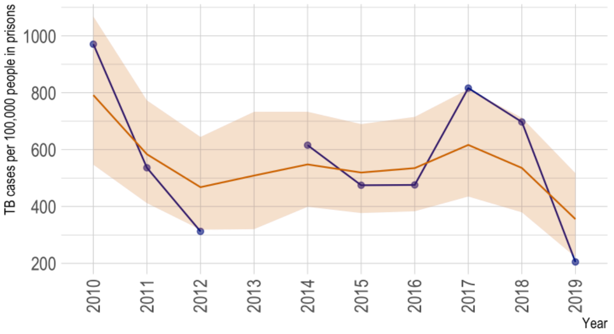

Luxembourg

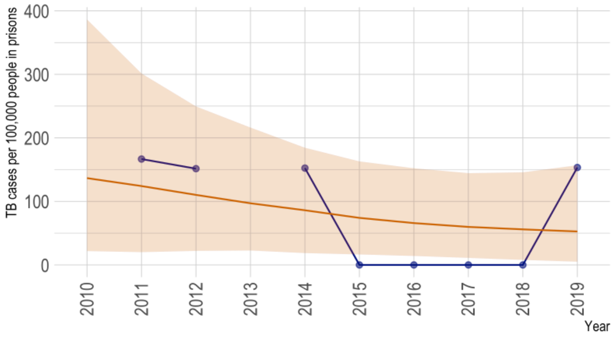

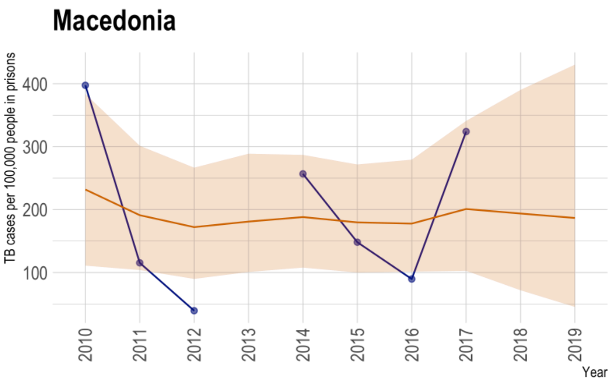
Macedonia
TB cases per 100,000 people in prisons
100
200
300
400
2010 2011 2012 2013 2014 2015 2016 2017 2018 2019
Year

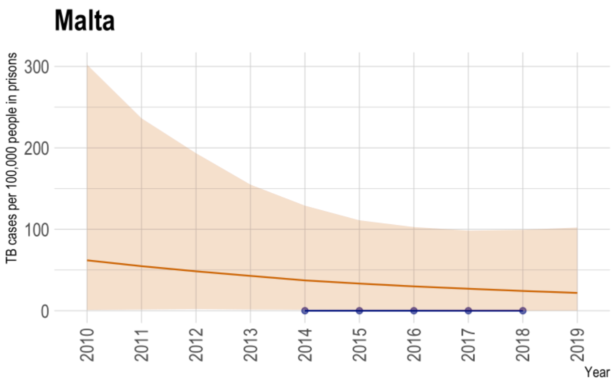
Malta
TB cases per 100,000 people in prisons
0
100
200
300
2010 2011 2012 2013 2014 2015 2016 2017 2018 2019
Year

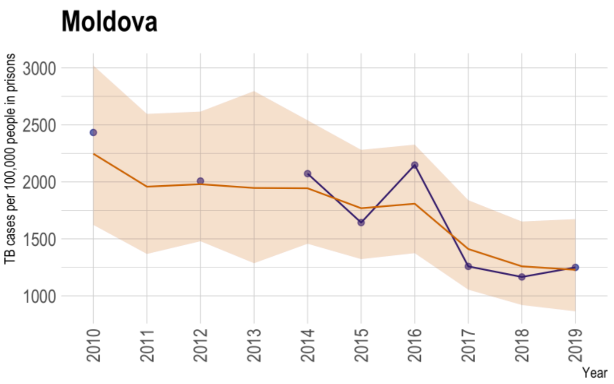
Moldova
TB cases per 100,000 people in prisons
1000
1500
2000
2500
3000
2010 2011 2012 2013 2014 2015 2016 2017 2018 2019
Year

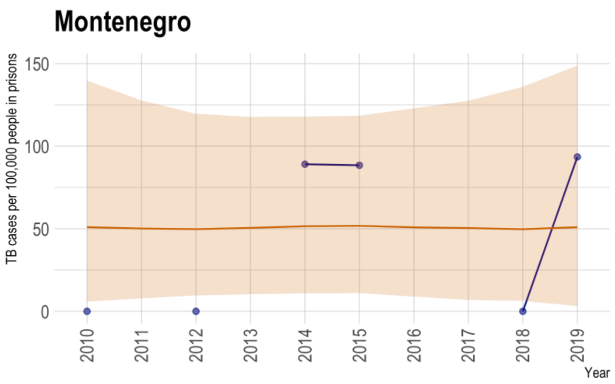
Montenegro
TB cases per 100,000 people in prisons
0
50
100
150
2010 2011 2012 2013 2014 2015 2016 2017 2018 2019
Year

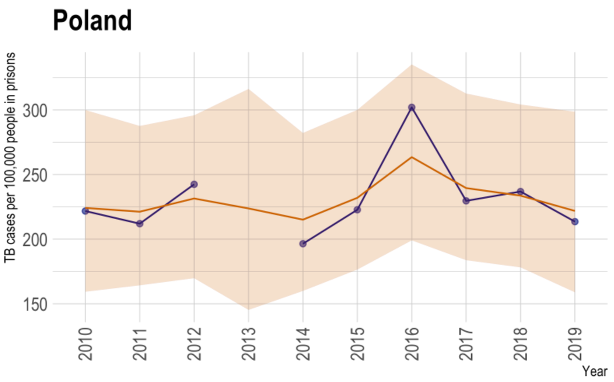
Poland
TB cases per 100,000 people in prisons
150
200
250
300
2010 2011 2012 2013 2014 2015 2016 2017 2018 2019
Year

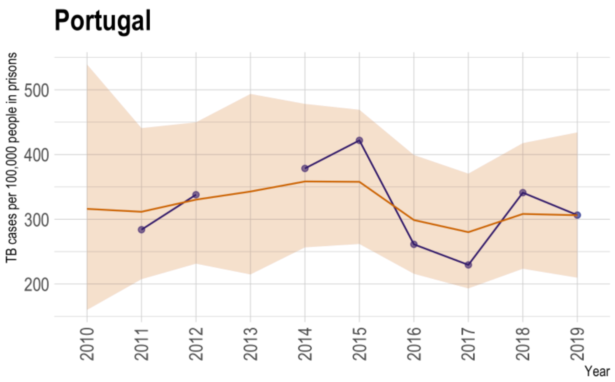
Portugal
TB cases per 100,000 people in prisons
200
300
400
500
2010 2011 2012 2013 2014 2015 2016 2017 2018 2019
Year

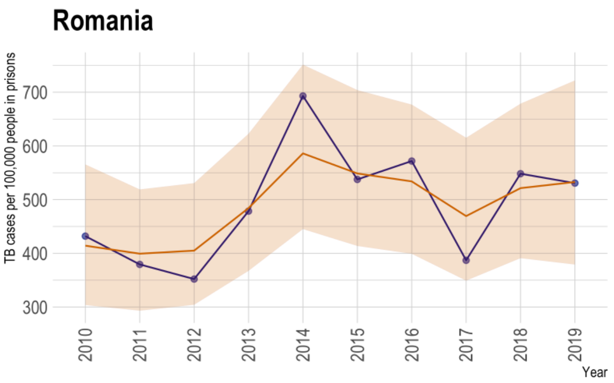
Romania
TB cases per 100,000 people in prisons
700
600
500
400
300
2010
2011
2012
2013
2014
2015
2016
2017
2018
2019
Year

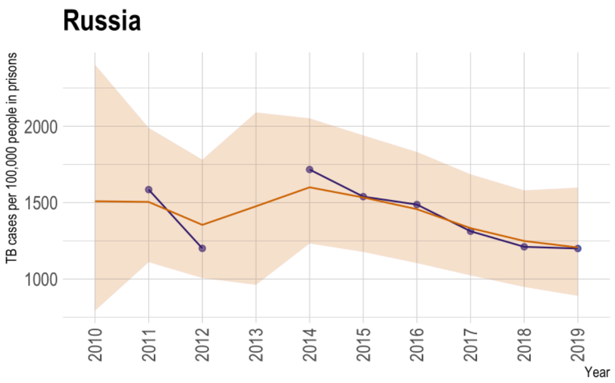
Russia
TB cases per 100,000 people in prisons
2000
1500
1000
2010
2011
2012
2013
2014
2015
2016
2017
2018
2019
Year

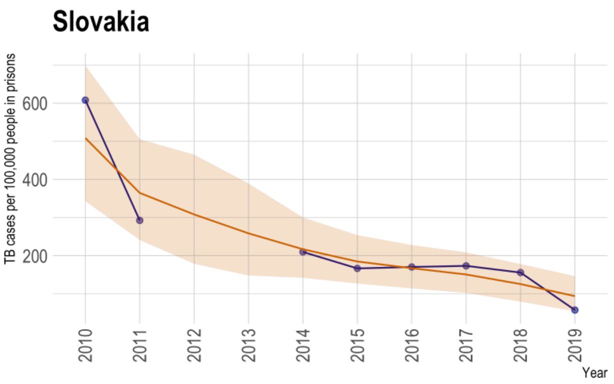
Slovakia
TB cases per 100,000 people in prisons
600
400
200
2010
2011
2012
2013
2014
2015
2016
2017
2018
2019
Year

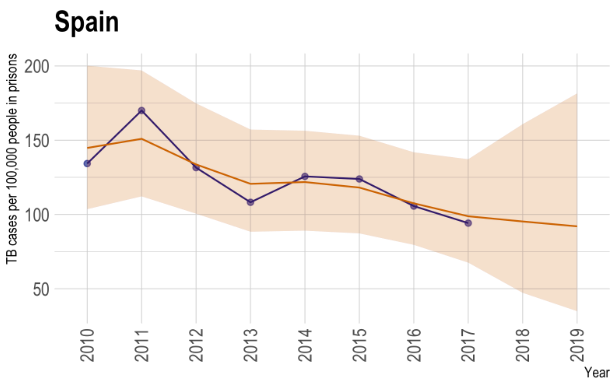
Spain
TB cases per 100,000 people in prisons
200
150
100
50
2010
2011
2012
2013
2014
2015
2016
2017
2018
2019
Year

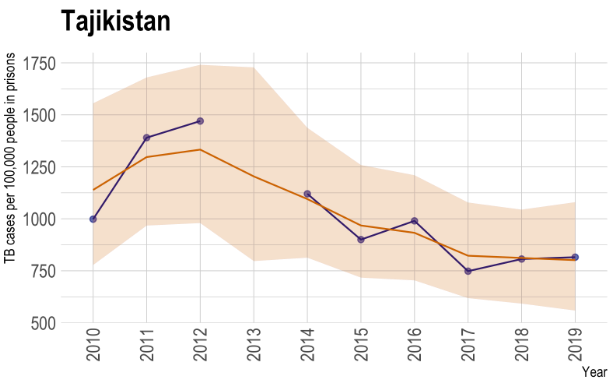
Tajikistan
TB cases per 100,000 people in prisons
1750
1500
1250
1000
750
500
2010
2011
2012
2013
2014
2015
2016
2017
2018
2019
Year

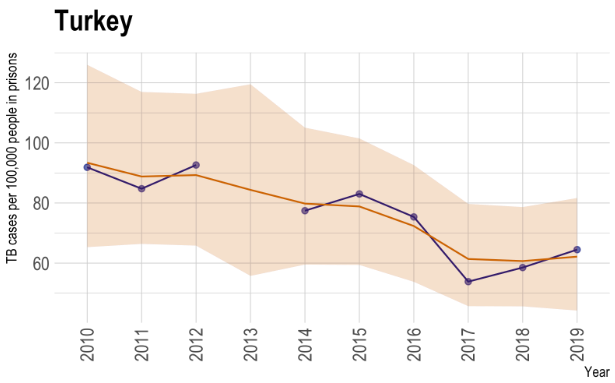
Turkey
TB cases per 100,000 people in prisons
120
100
80
60
2010
2011
2012
2013
2014
2015
2016
2017
2018
2019
Year

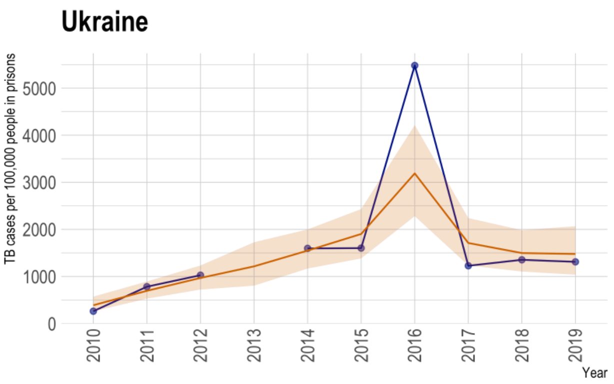
Ukraine
TB cases per 100,000 people in prisons
5000
4000
3000
2000
1000
0
2010
2011
2012
2013
2014
2015
2016
2017
2018
2019
Year

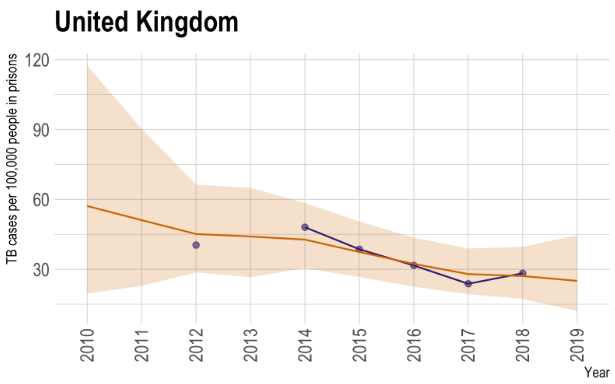
United Kingdom
TB cases per 100,000 people in prisons
120
90
60
30
2010
2011
2012
2013
2014
2015
2016
2017
2018
2019
Year

TB case notifications: American Region

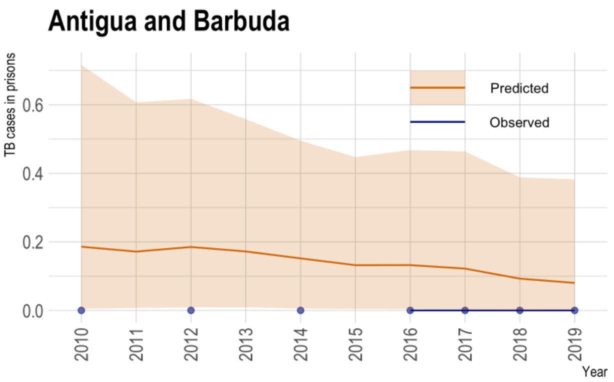

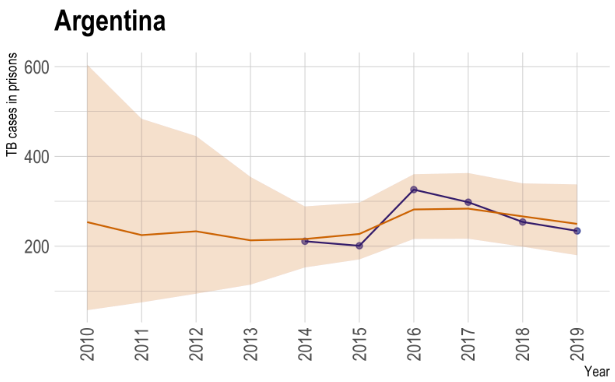

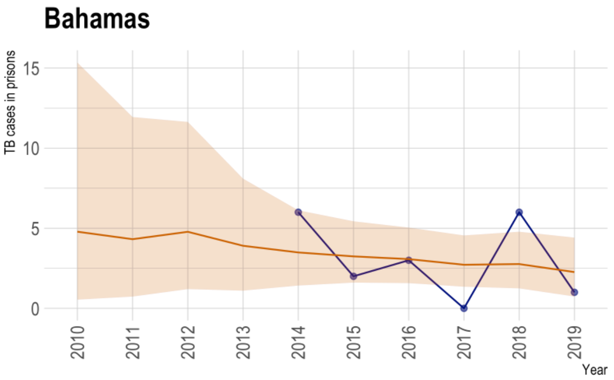

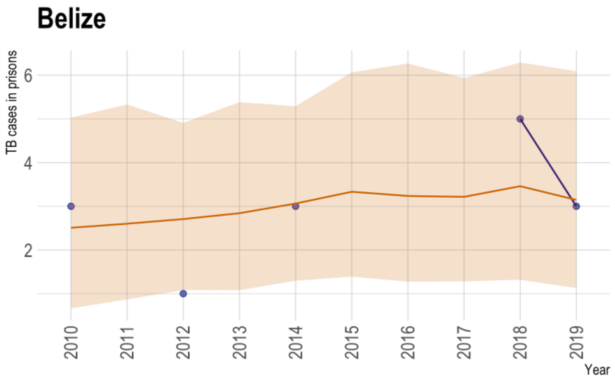

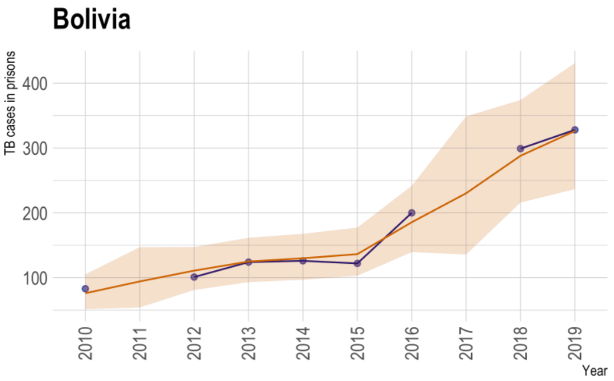

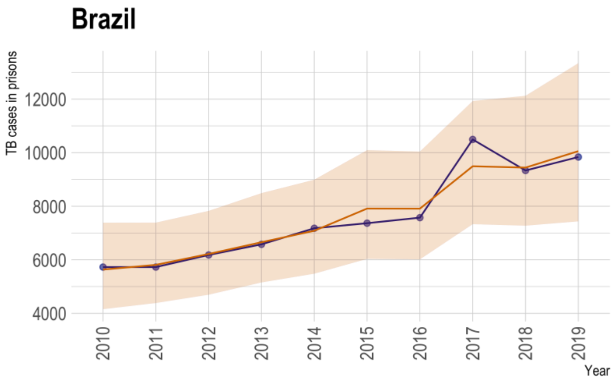

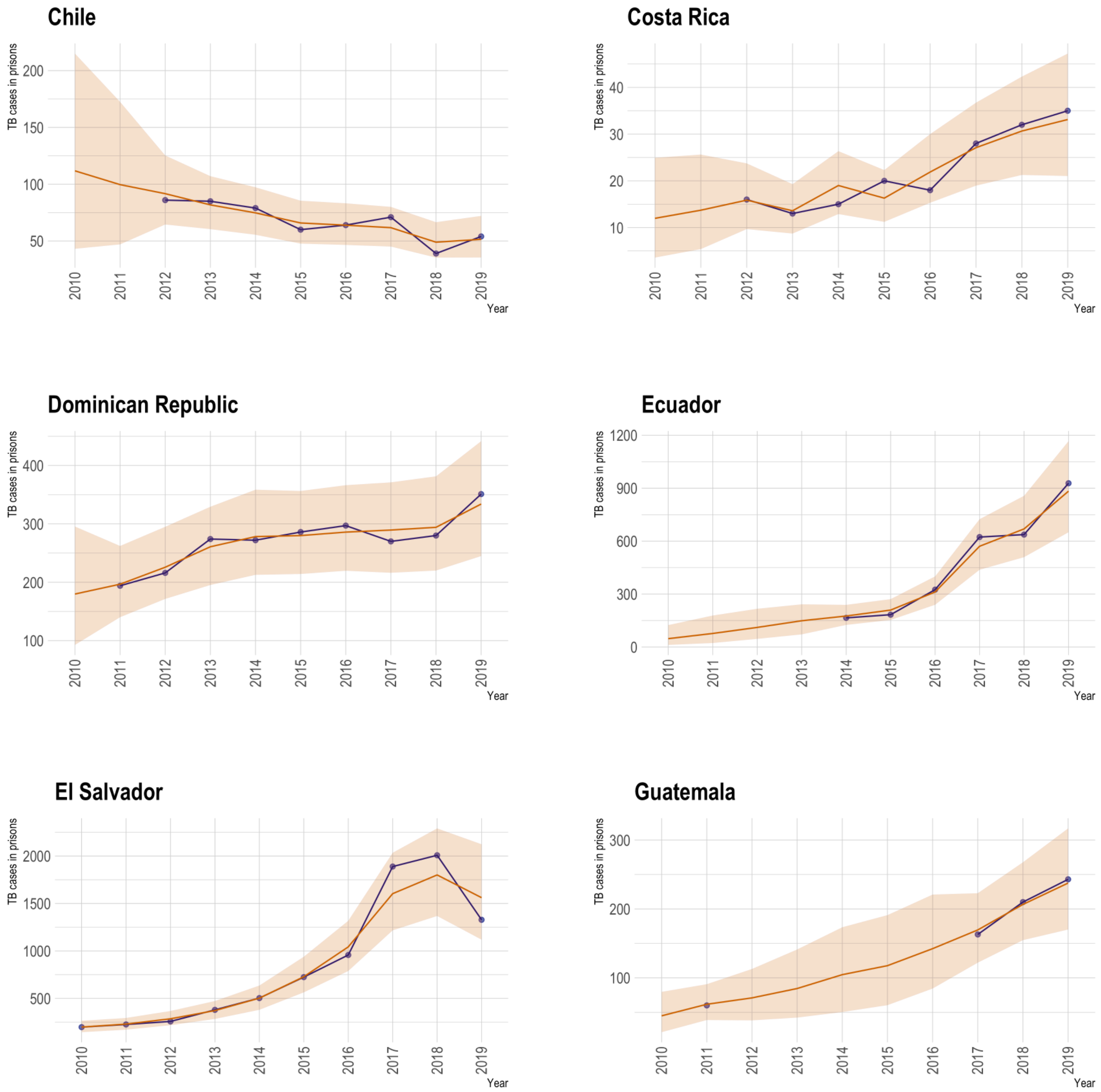
Chile
Costa Rica
Dominican Republic
Ecuador
El Salvador
Guatemala
TB cases in prisons
Year
2010
2011
2012
2013
2014
2015
2016
2017
2018
2019

Guyana

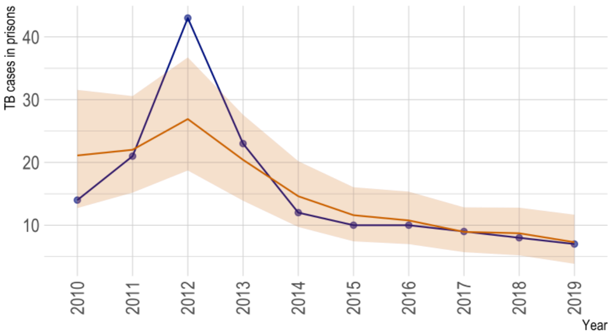

Haiti

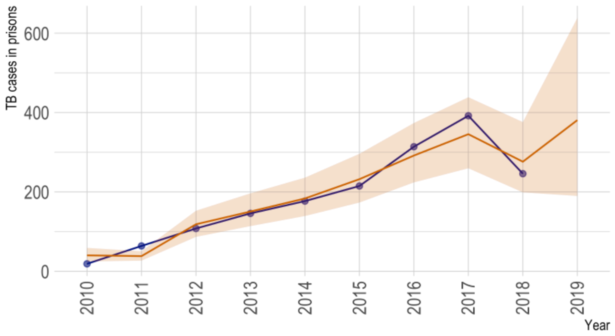

Honduras

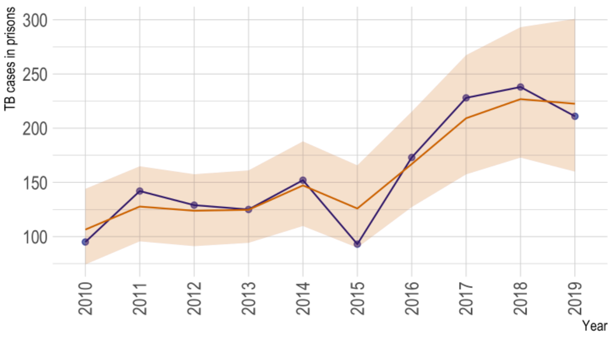

Jamaica

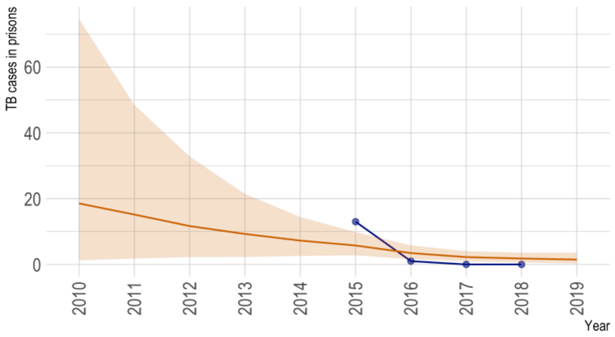

Mexico

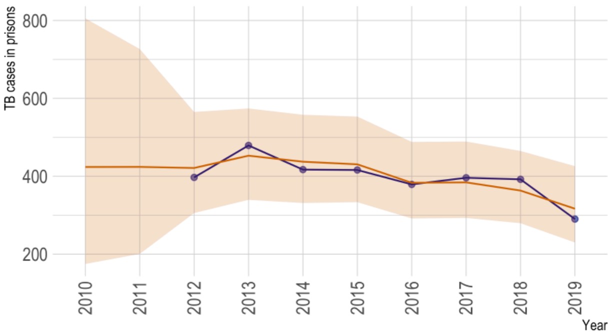

Nicaragua

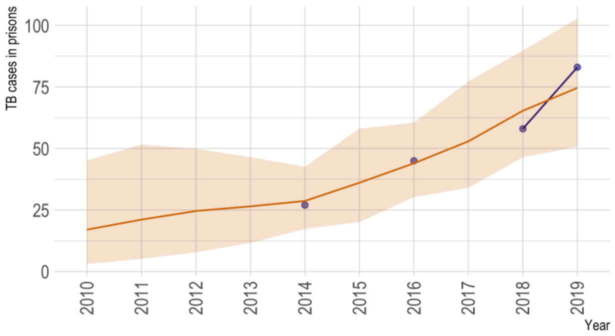

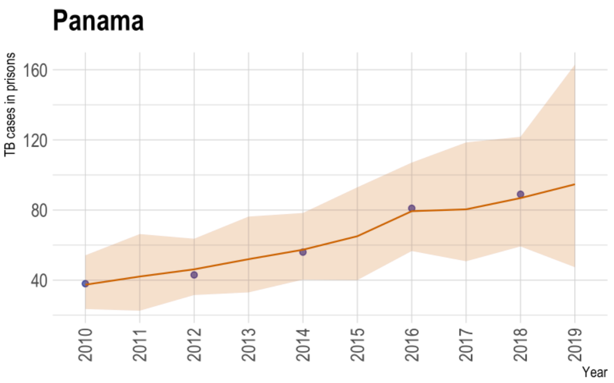

Panama
TB cases in prisons
160
120
80
40
2010
2011
2012
2013
2014
2015
2016
2017
2018
2019
Year

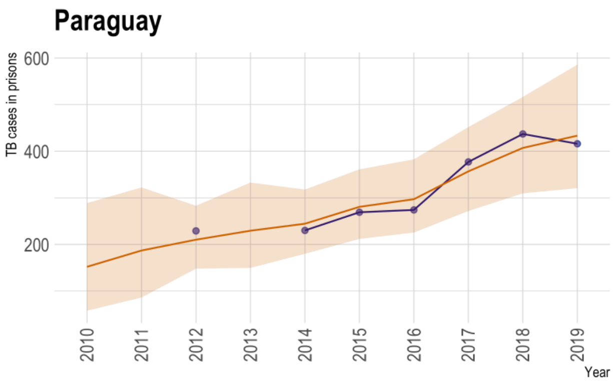

Paraguay
TB cases in prisons
600
400
200
2010
2011
2012
2013
2014
2015
2016
2017
2018
2019
Year

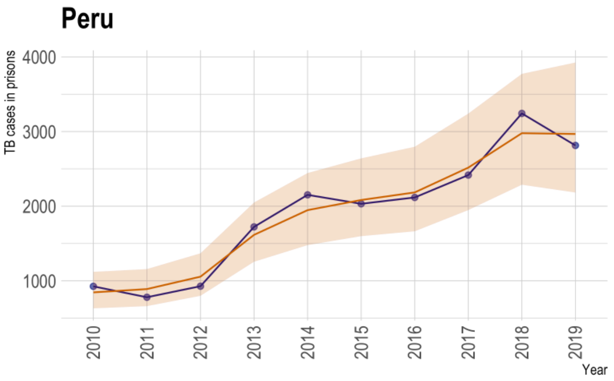

Peru
TB cases in prisons
4000
3000
2000
1000
2010
2011
2012
2013
2014
2015
2016
2017
2018
2019
Year

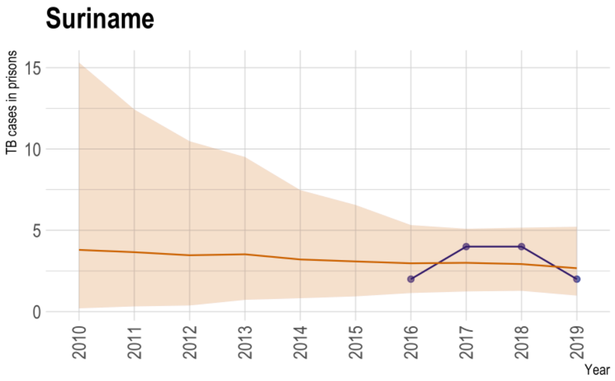

Suriname
TB cases in prisons
15
10
5
0
2010
2011
2012
2013
2014
2015
2016
2017
2018
2019
Year

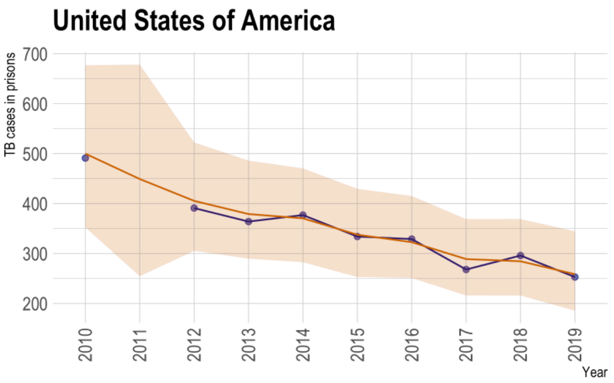
United States of America
TB cases in prisons
700
600
500
400
300
200
2010
2011
2012
2013
2014
2015
2016
2017
2018
2019
Year

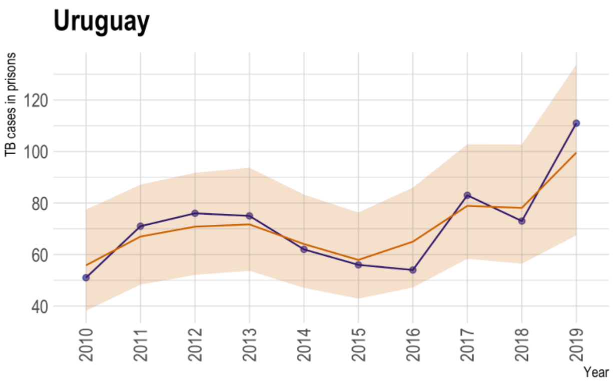
Uruguay
TB cases in prisons
120
100
80
60
40
2010
2011
2012
2013
2014
2015
2016
2017
2018
2019
Year

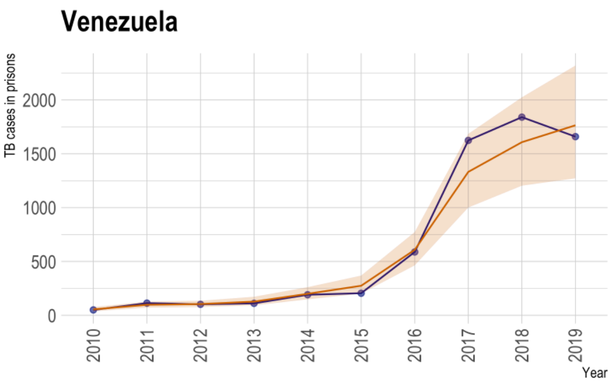
Venezuela
TB cases in prisons
2000
1500
1000
500
0
2010
2011
2012
2013
2014
2015
2016
2017
2018
2019
Year

TB case notifications: European Region

Albania

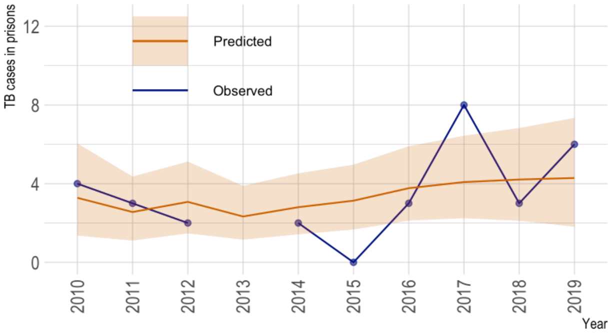

Andorra

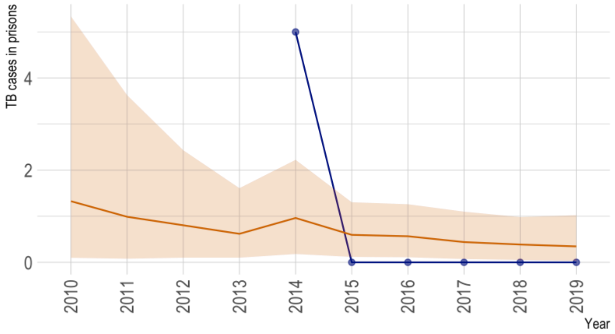

Armenia

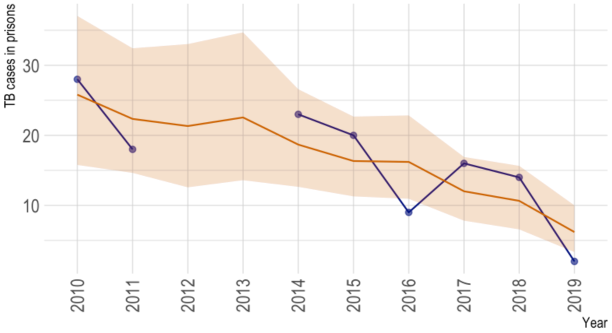

Austria

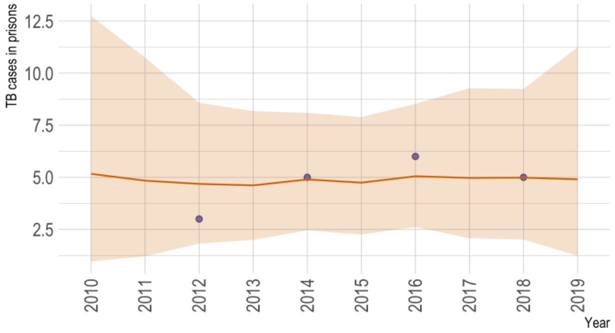

Azerbaijan

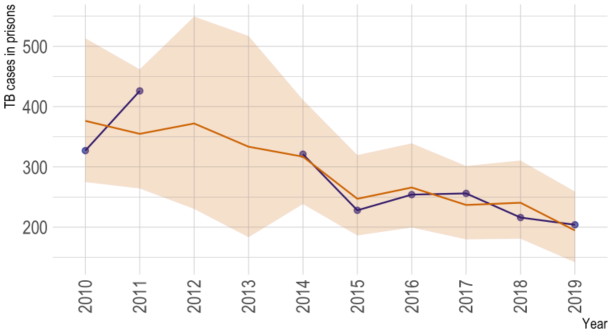

Belarus

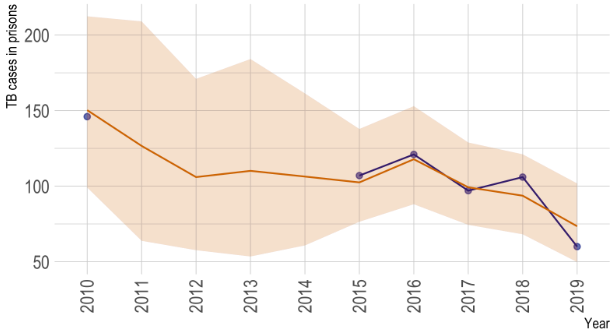

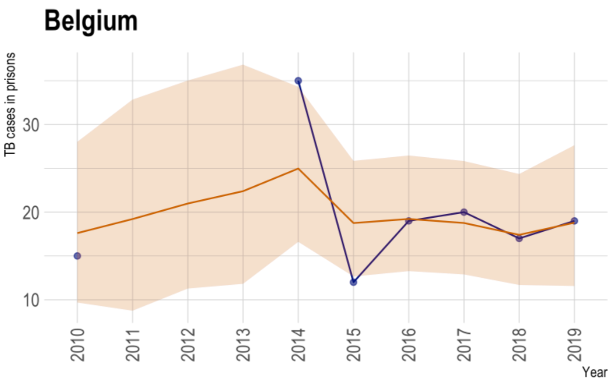
Belgium
TB cases in prisons
30
20
10
2010
2011
2012
2013
2014
2015
2016
2017
2018
2019
Year

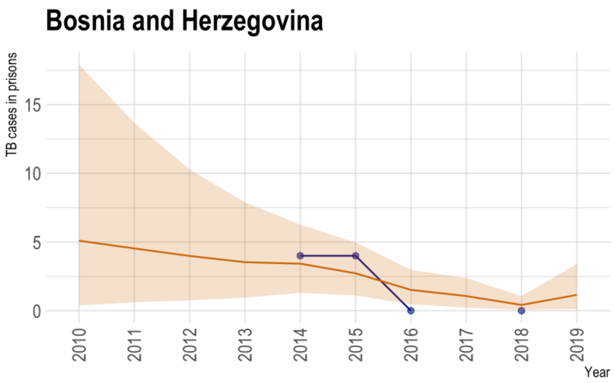
Bosnia and Herzegovina
TB cases in prisons
15
10
5
0
2010
2011
2012
2013
2014
2015
2016
2017
2018
2019
Year

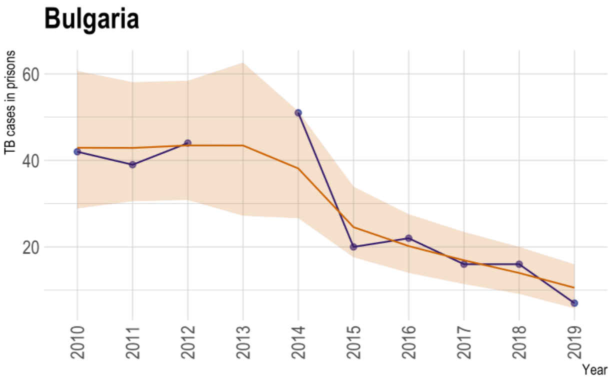
Bulgaria
TB cases in prisons
60
40
20
2010
2011
2012
2013
2014
2015
2016
2017
2018
2019
Year

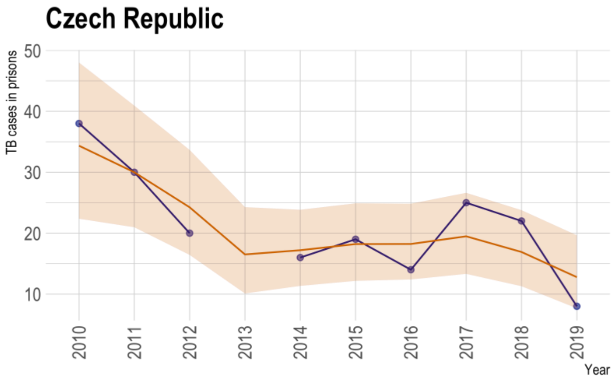
Czech Republic
TB cases in prisons
50
40
30
20
10
2010
2011
2012
2013
2014
2015
2016
2017
2018
2019
Year

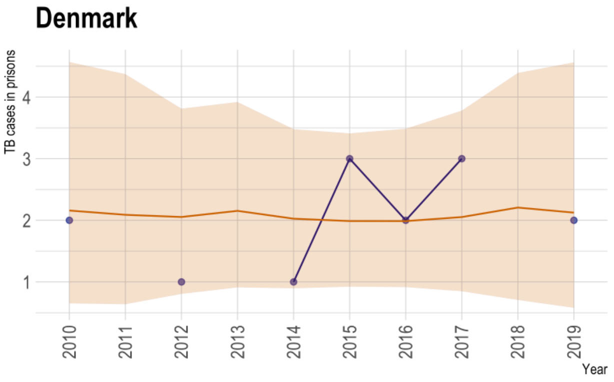
Denmark
TB cases in prisons
4
3
2
1
2010
2011
2012
2013
2014
2015
2016
2017
2018
2019
Year

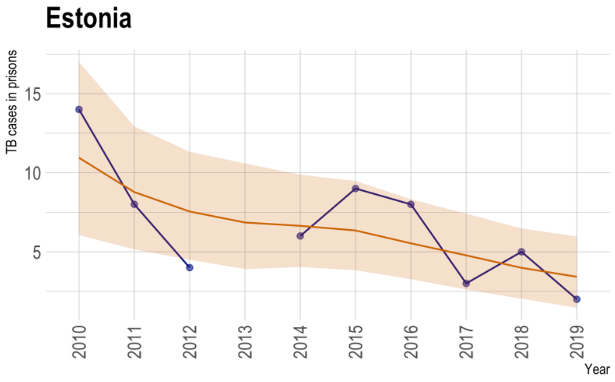
Estonia
TB cases in prisons
15
10
5
2010
2011
2012
2013
2014
2015
2016
2017
2018
2019
Year

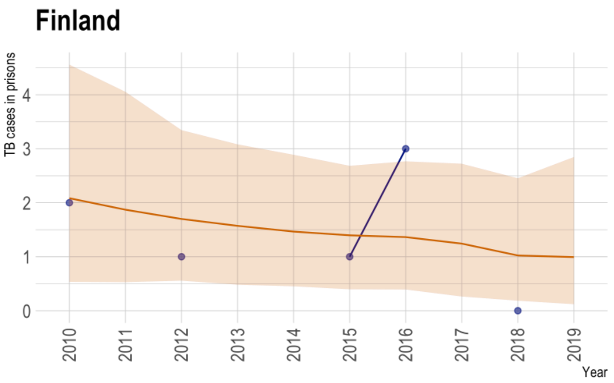
Finland
TB cases in prisons
0
1
2
3
4
2010
2011
2012
2013
2014
2015
2016
2017
2018
2019
Year

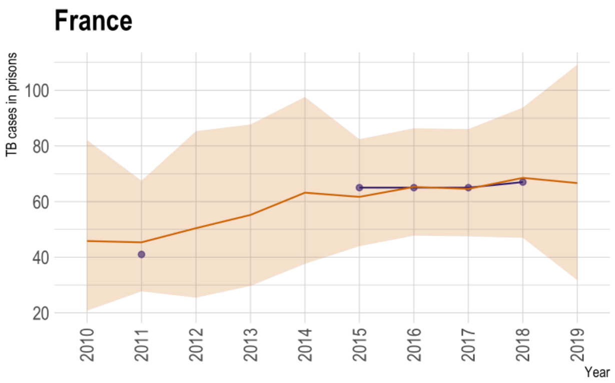
France
TB cases in prisons
20
40
60
80
100
2010
2011
2012
2013
2014
2015
2016
2017
2018
2019
Year

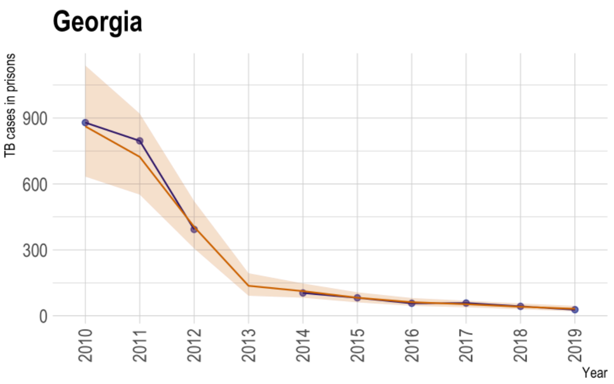
Georgia
TB cases in prisons
0
300
600
900
2010
2011
2012
2013
2014
2015
2016
2017
2018
2019
Year

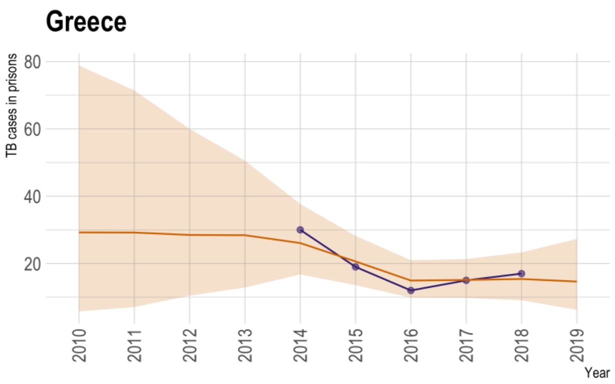
Greece
TB cases in prisons
20
40
60
80
2010
2011
2012
2013
2014
2015
2016
2017
2018
2019
Year

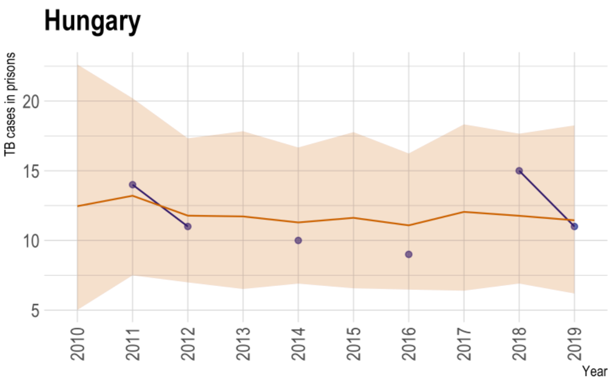
Hungary
TB cases in prisons
5
10
15
20
2010
2011
2012
2013
2014
2015
2016
2017
2018
2019
Year

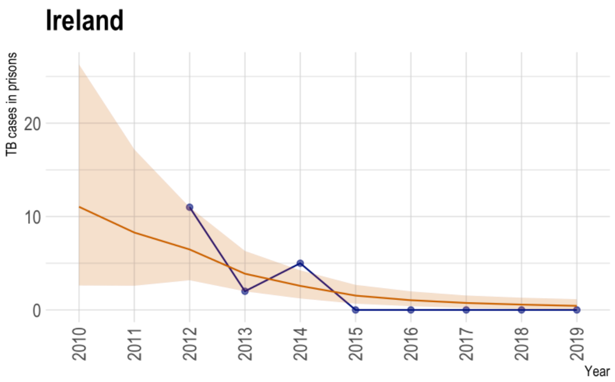
Ireland
TB cases in prisons
0
10
20
2010
2011
2012
2013
2014
2015
2016
2017
2018
2019
Year

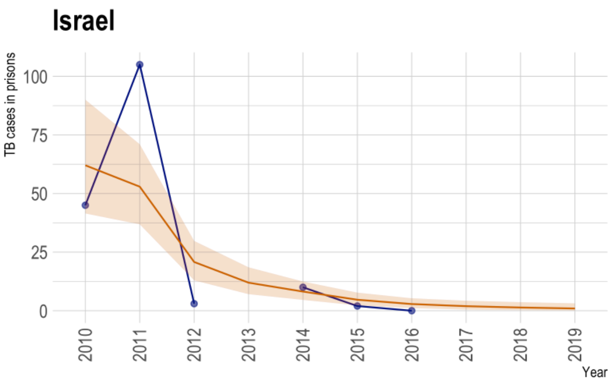
Israel
TB cases in prisons
100
75
50
25
0
2010 2011 2012 2013 2014 2015 2016 2017 2018 2019
Year

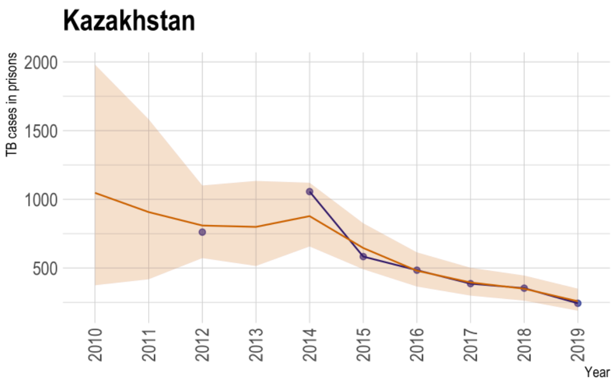
Kazakhstan
TB cases in prisons
2000
1500
1000
500
2010 2011 2012 2013 2014 2015 2016 2017 2018 2019
Year

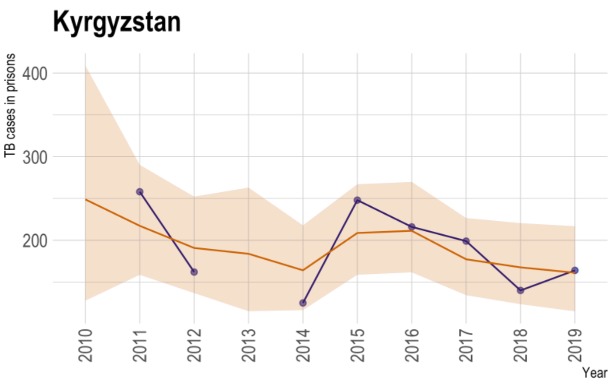
Kyrgyzstan
TB cases in prisons
400
300
200
2010 2011 2012 2013 2014 2015 2016 2017 2018 2019
Year

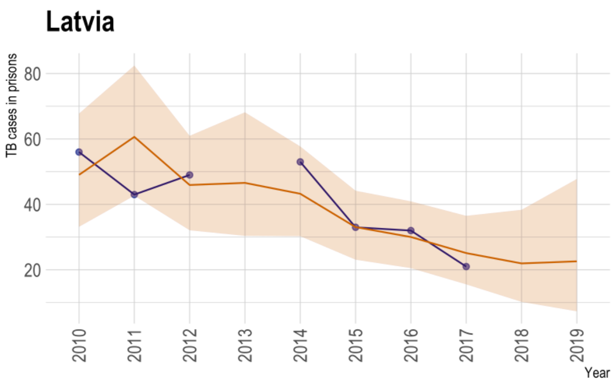
Latvia
TB cases in prisons
80
60
40
20
2010 2011 2012 2013 2014 2015 2016 2017 2018 2019
Year

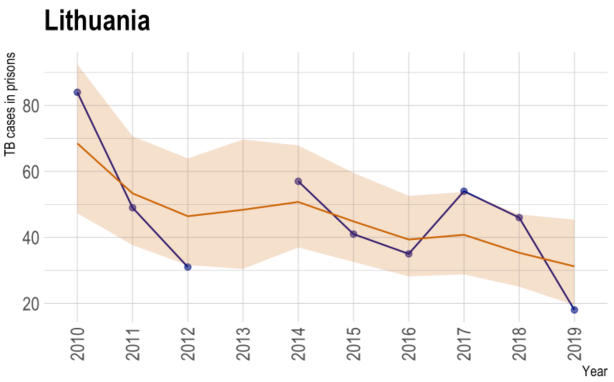
Lithuania
TB cases in prisons
80
60
40
20
2010 2011 2012 2013 2014 2015 2016 2017 2018 2019
Year

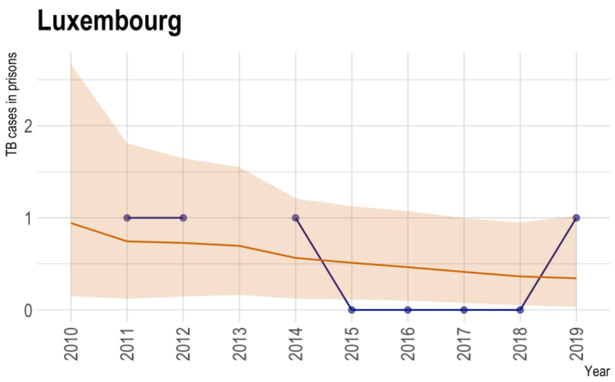
Luxembourg
TB cases in prisons
2
1
0
2010 2011 2012 2013 2014 2015 2016 2017 2018 2019
Year

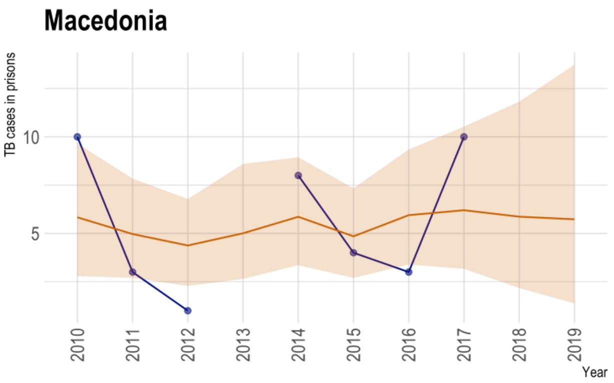
Macedonia
TB cases in prisons
10
5
2010 2011 2012 2013 2014 2015 2016 2017 2018 2019
Year

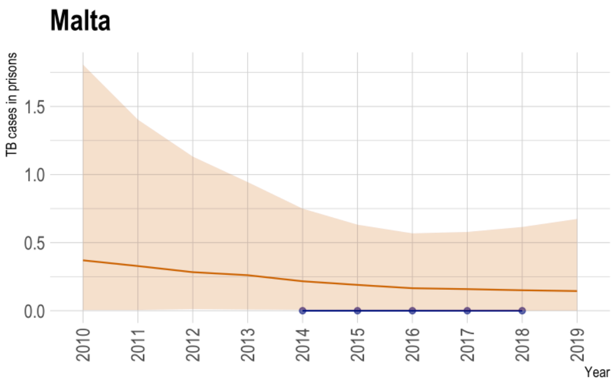
Malta
TB cases in prisons
1.5
1.0
0.5
0.0
2010 2011 2012 2013 2014 2015 2016 2017 2018 2019
Year

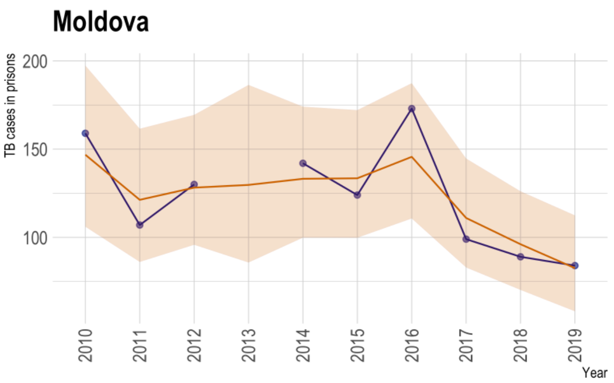
Moldova
TB cases in prisons
200
150
100
2010 2011 2012 2013 2014 2015 2016 2017 2018 2019
Year

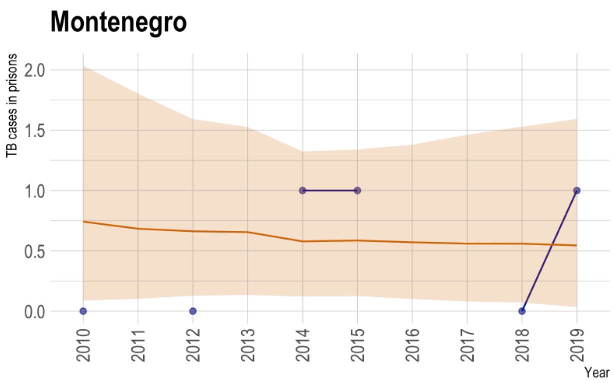
Montenegro
TB cases in prisons
2.0
1.5
1.0
0.5
0.0
2010 2011 2012 2013 2014 2015 2016 2017 2018 2019
Year

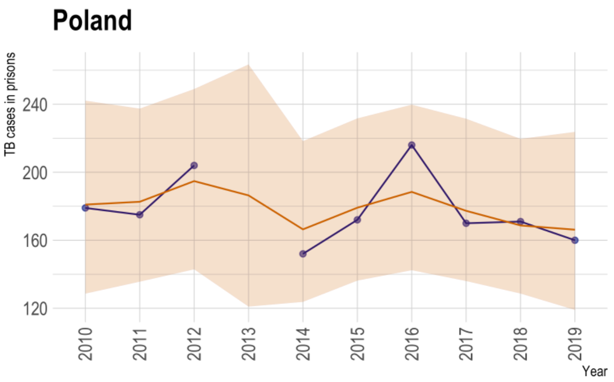
Poland
TB cases in prisons
240
200
160
120
2010 2011 2012 2013 2014 2015 2016 2017 2018 2019
Year

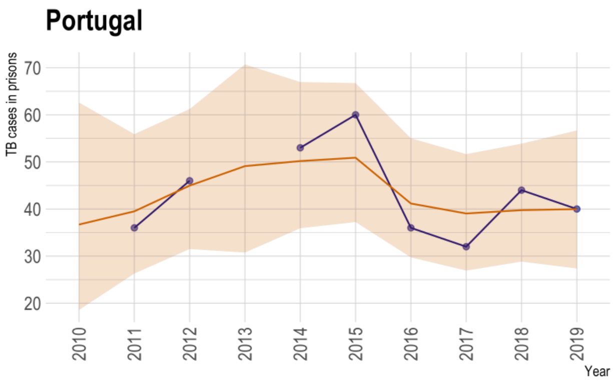
Portugal
TB cases in prisons
70
60
50
40
30
20
2010 2011 2012 2013 2014 2015 2016 2017 2018 2019
Year

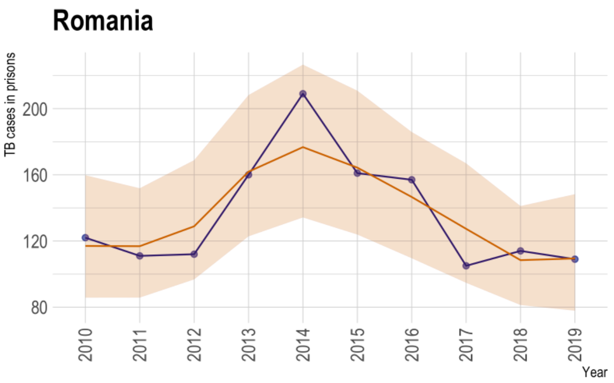
Romania
TB cases in prisons
80
120
160
200
2010
2011
2012
2013
2014
2015
2016
2017
2018
2019
Year

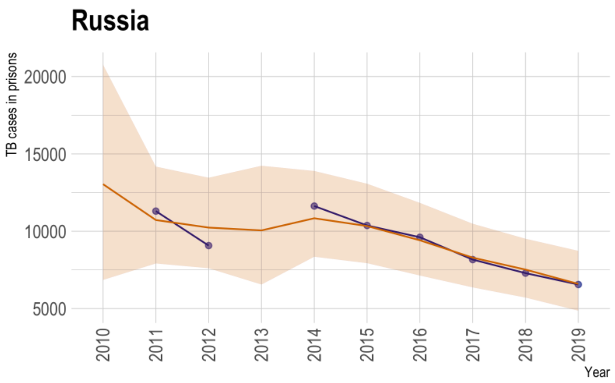
Russia
TB cases in prisons
5000
10000
15000
20000
2010
2011
2012
2013
2014
2015
2016
2017
2018
2019
Year

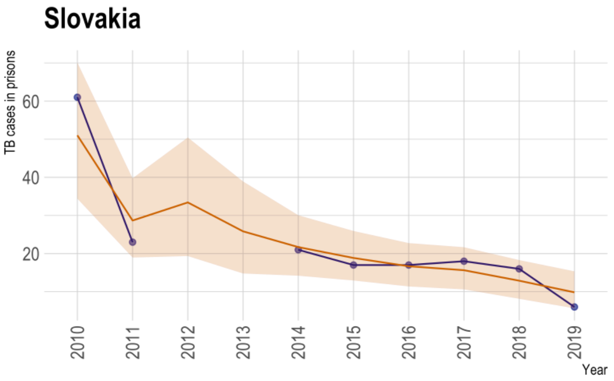
Slovakia
TB cases in prisons
20
40
60
2010
2011
2012
2013
2014
2015
2016
2017
2018
2019
Year

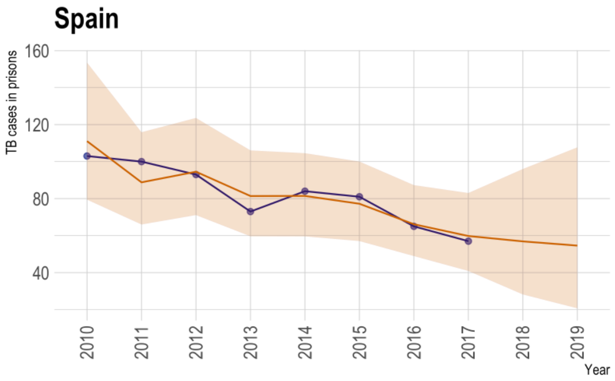
Spain
TB cases in prisons
40
80
120
160
2010
2011
2012
2013
2014
2015
2016
2017
2018
2019
Year

Tajikistan

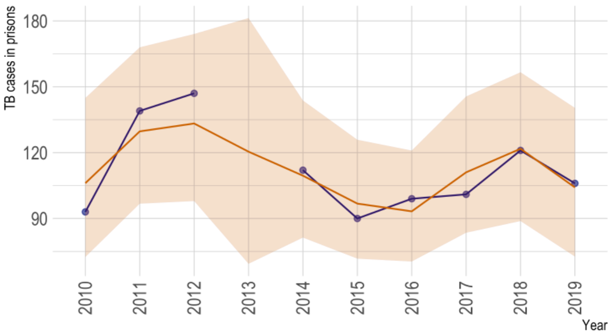

Turkey

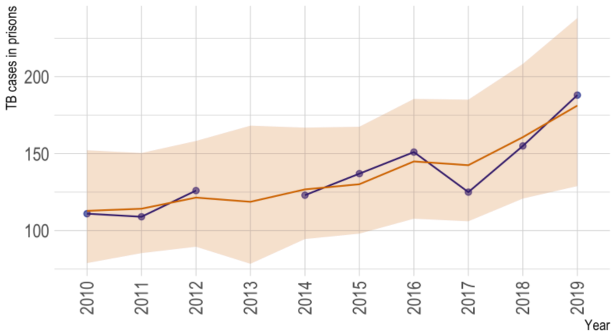

Ukraine

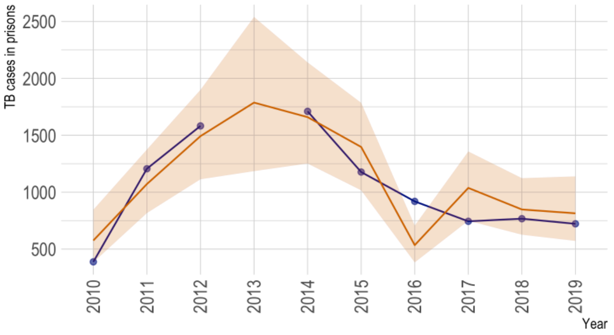

United Kingdom

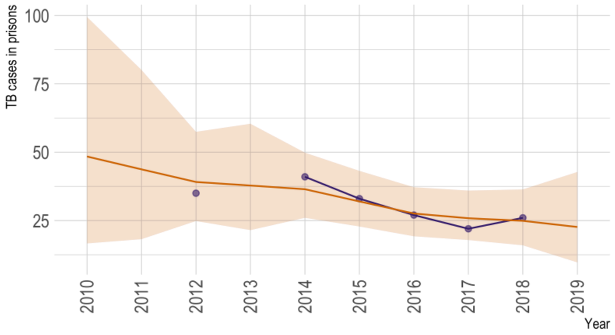

Prison population: American Region

Note: observed values for prison population are used in the model, unless missing (in which case a value is imputed)

Antigua and Barbuda

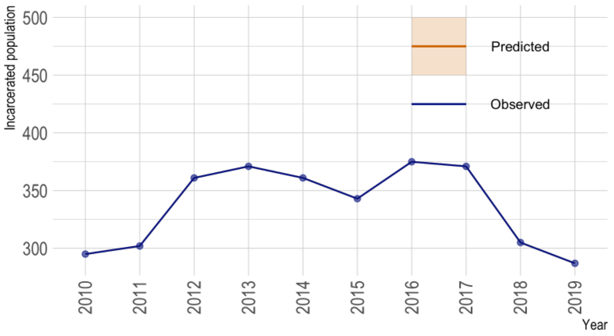

Argentina

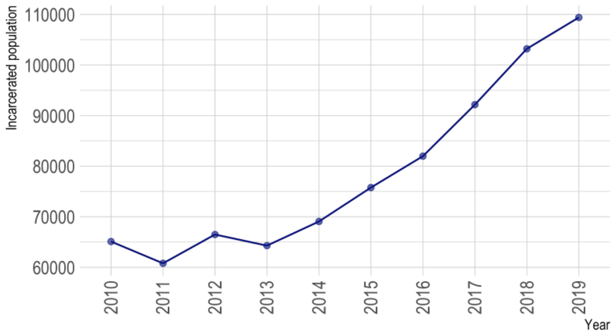

Bahamas

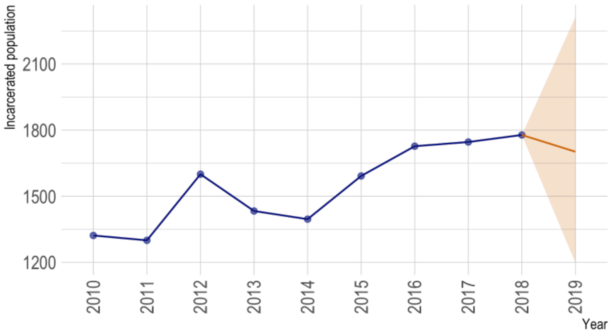

Belize

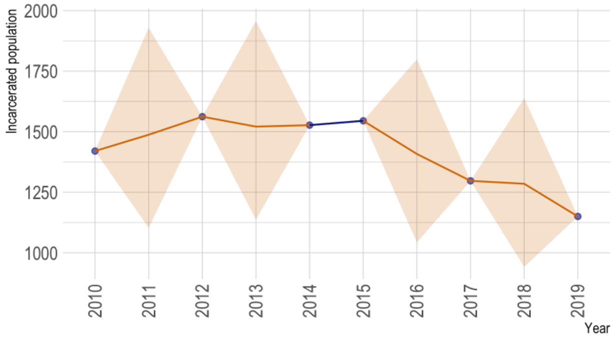

Bolivia

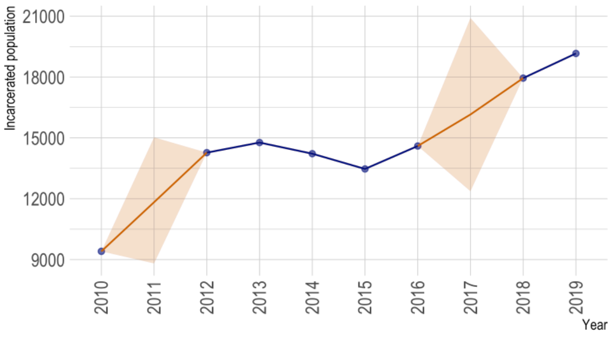

Brazil

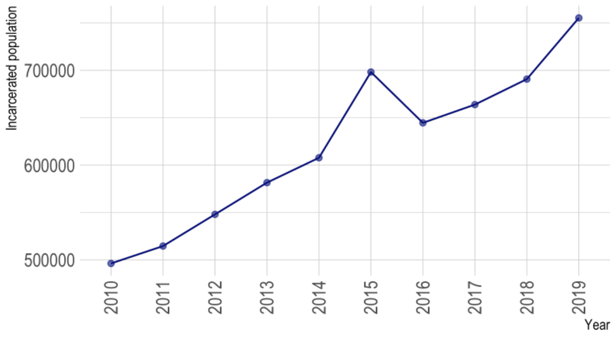

Chile

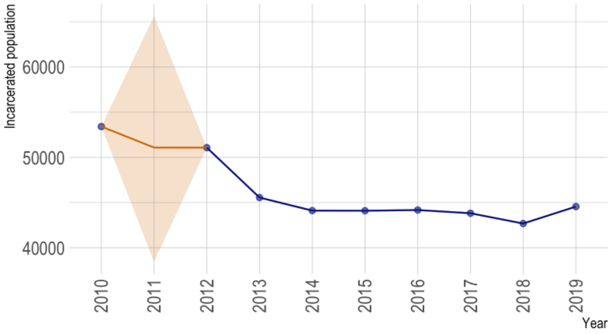

Costa Rica

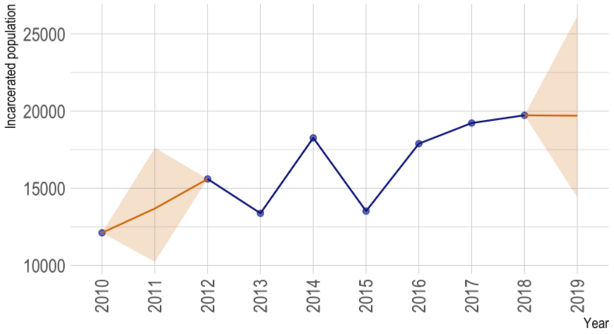

Dominican Republic

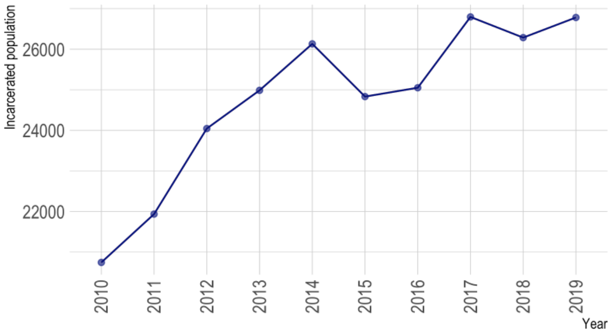

Ecuador

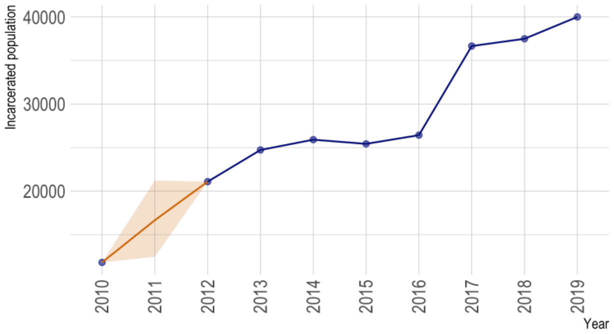

El Salvador

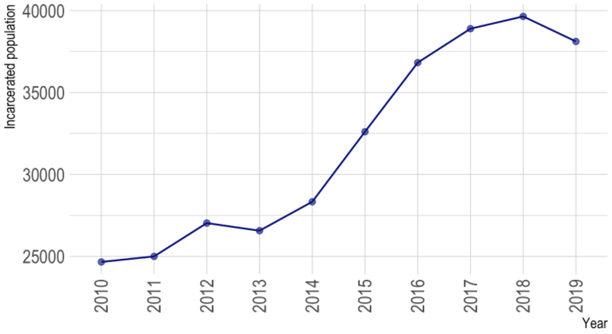

Guatemala

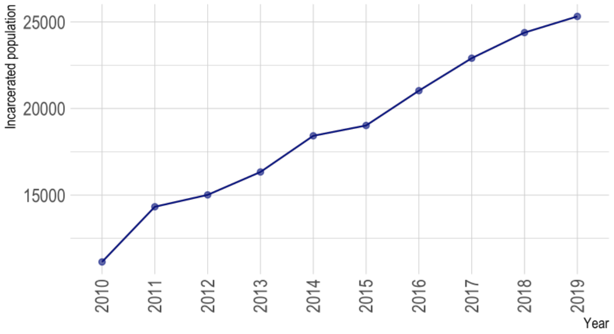

Guyana

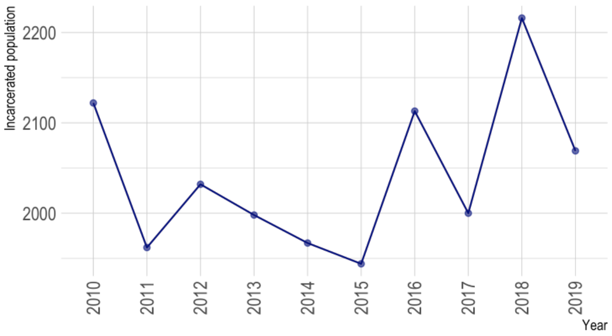

Haiti

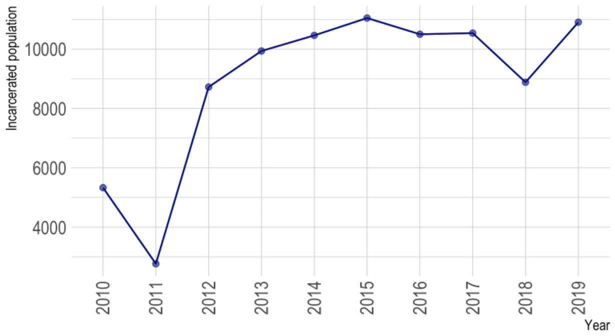

Honduras

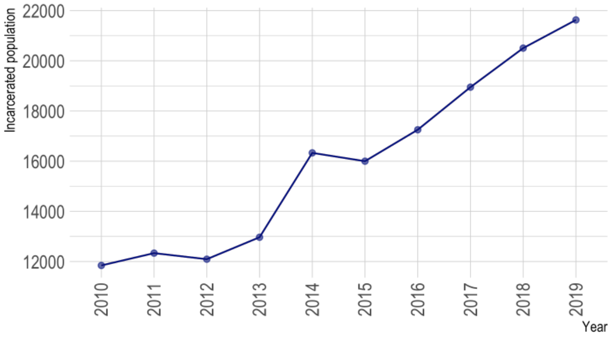

Jamaica

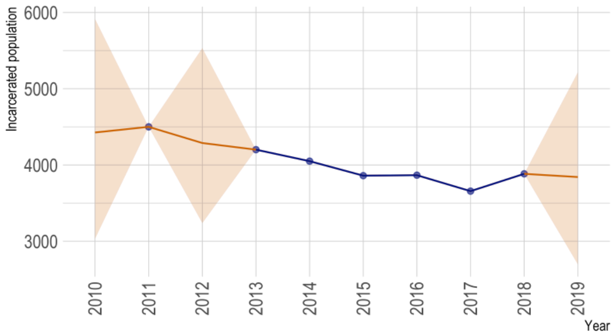

Mexico

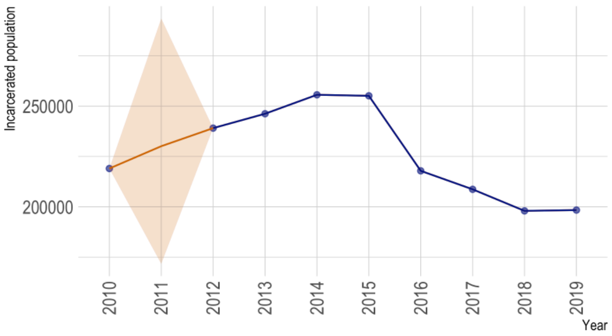

Nicaragua

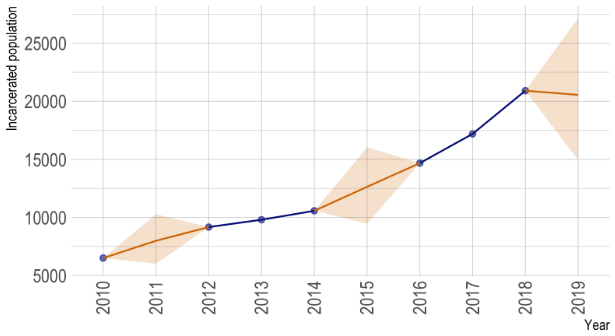

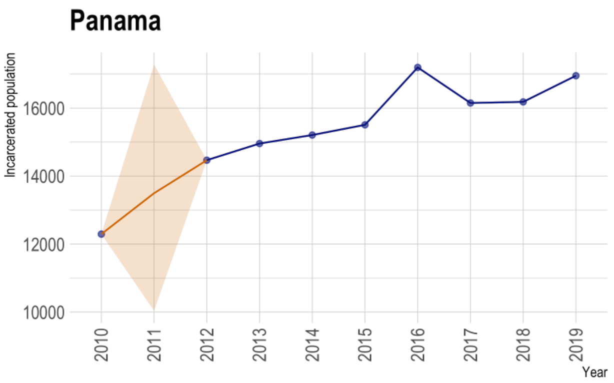
Panama
Incarcerated population
10000
12000
14000
16000
2010
2011
2012
2013
2014
2015
2016
2017
2018
2019
Year

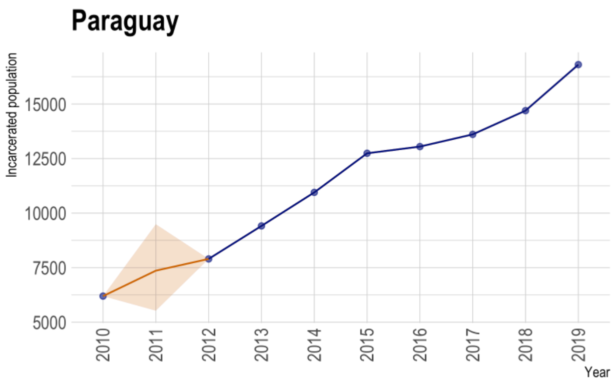
Paraguay
Incarcerated population
5000
7500
10000
12500
15000
2010
2011
2012
2013
2014
2015
2016
2017
2018
2019
Year

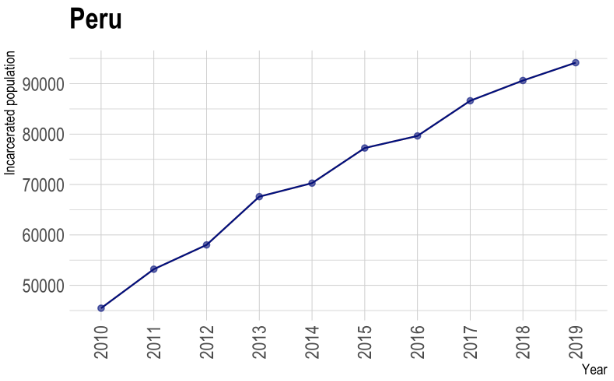
Peru
Incarcerated population
50000
60000
70000
80000
90000
2010
2011
2012
2013
2014
2015
2016
2017
2018
2019
Year

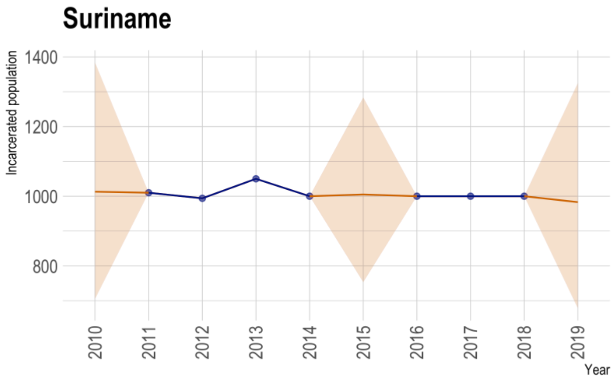
Suriname
Incarcerated population
800
1000
1200
1400
2010
2011
2012
2013
2014
2015
2016
2017
2018
2019
Year

United States of America

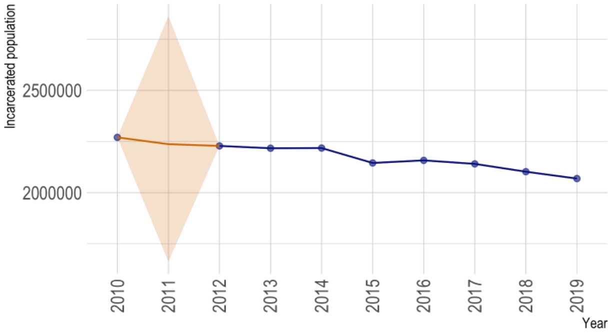

Uruguay

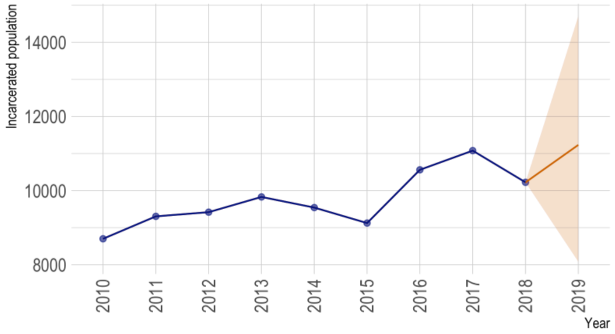

Venezuela

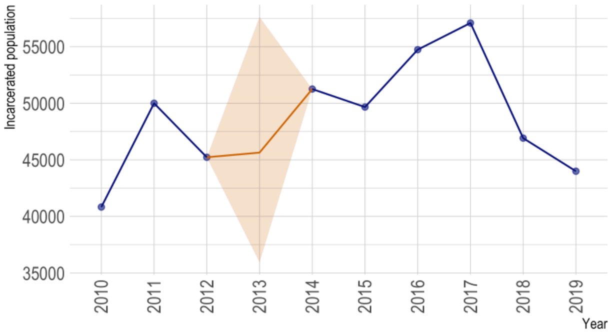

Prison population: European Region

Note: observed values for prison population are used in the model, unless missing (in which case a value is imputed)

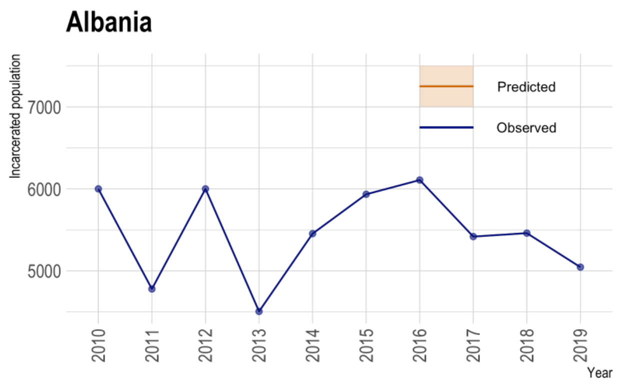

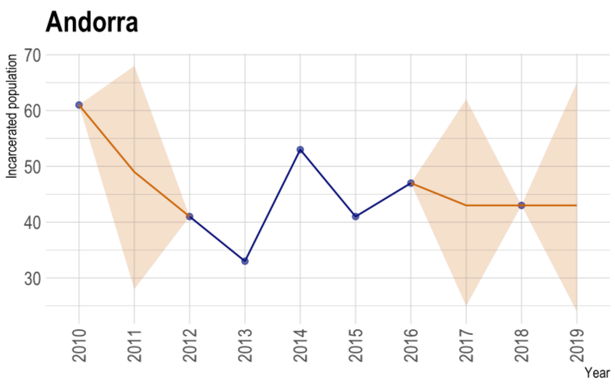

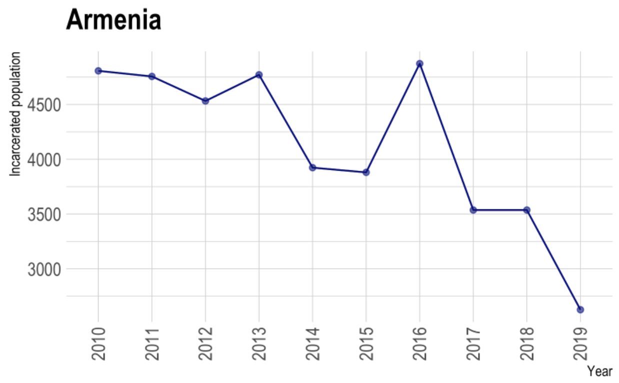

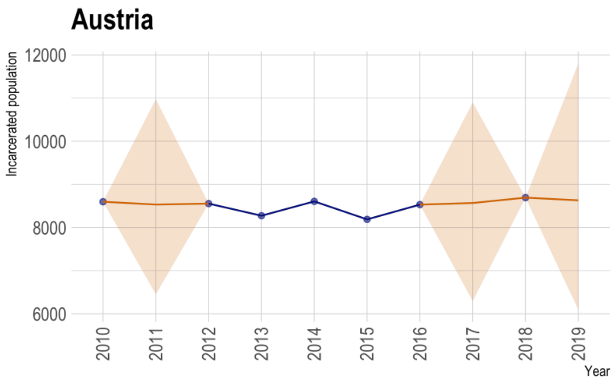

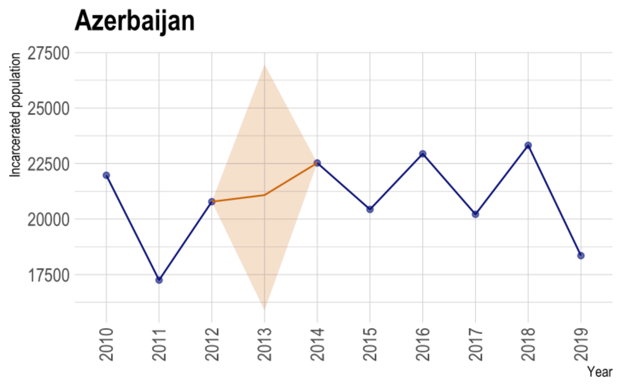

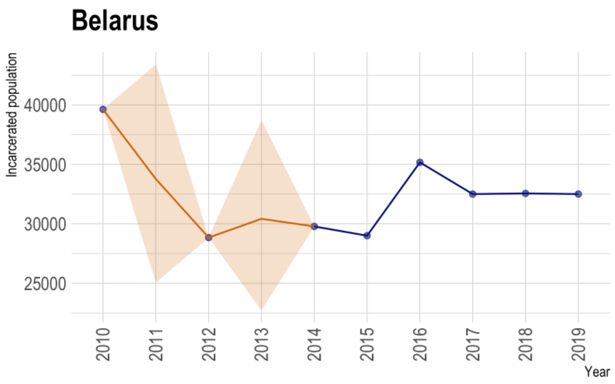

Belgium

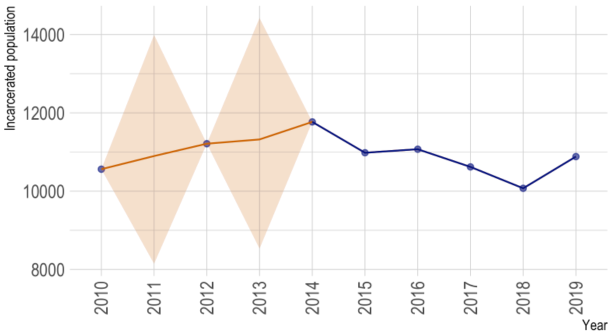

Bosnia and Herzegovina

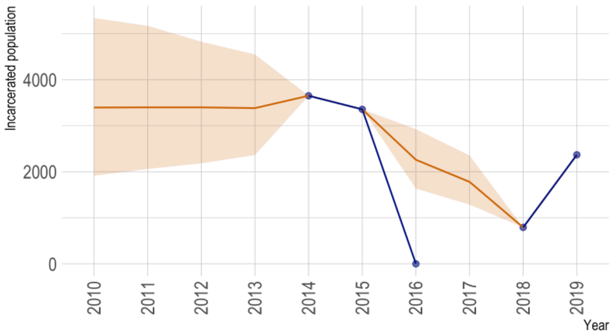

Bulgaria

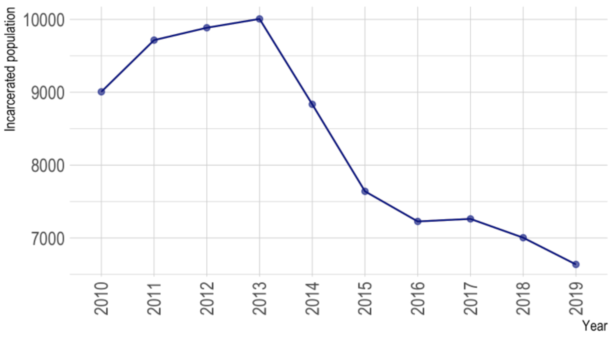

Czech Republic

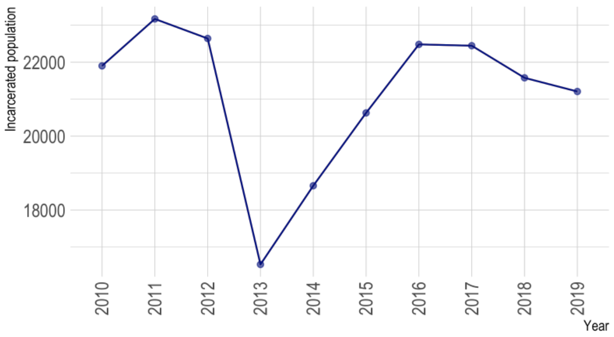

Denmark

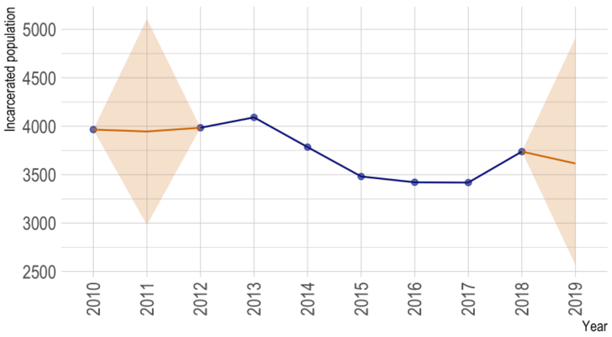

Estonia

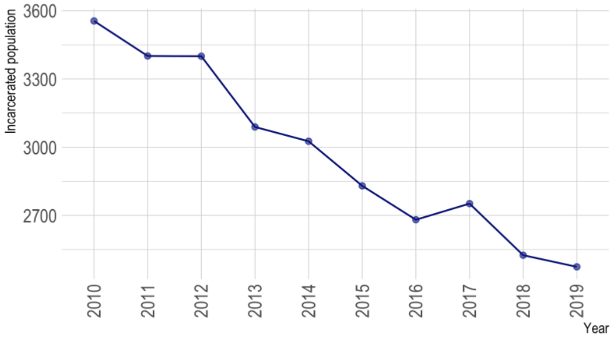

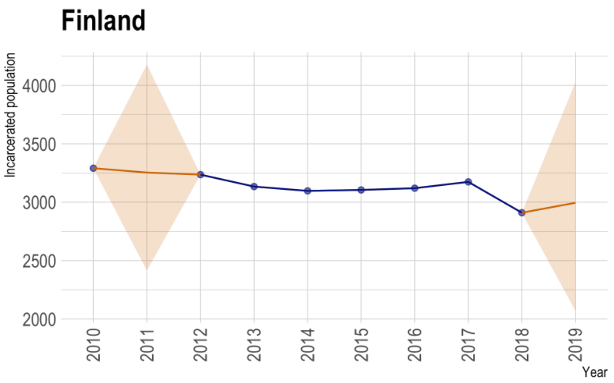
Finland
Incarcerated population
4000
3500
3000
2500
2000
2010
2011
2012
2013
2014
2015
2016
2017
2018
2019
Year

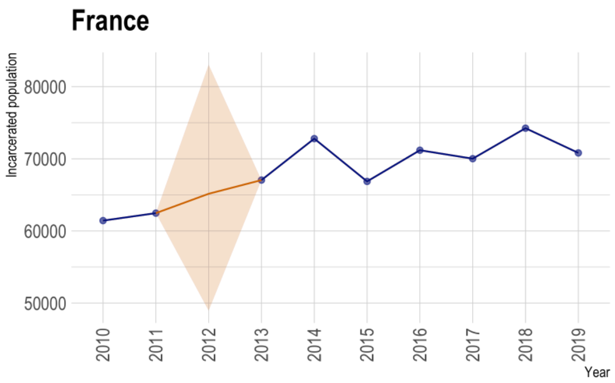
France
Incarcerated population
80000
70000
60000
50000
2010
2011
2012
2013
2014
2015
2016
2017
2018
2019
Year

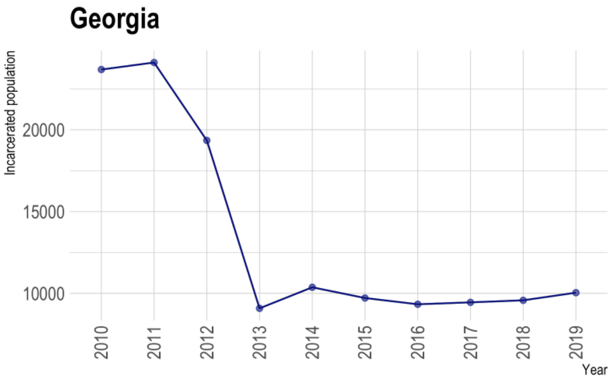
Georgia
Incarcerated population
20000
15000
10000
2010
2011
2012
2013
2014
2015
2016
2017
2018
2019
Year

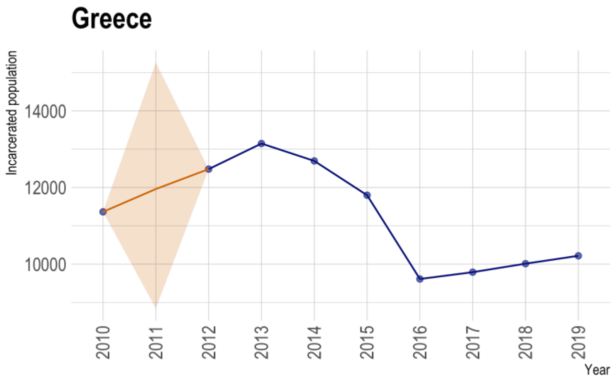
Greece
Incarcerated population
14000
12000
10000
2010
2011
2012
2013
2014
2015
2016
2017
2018
2019
Year

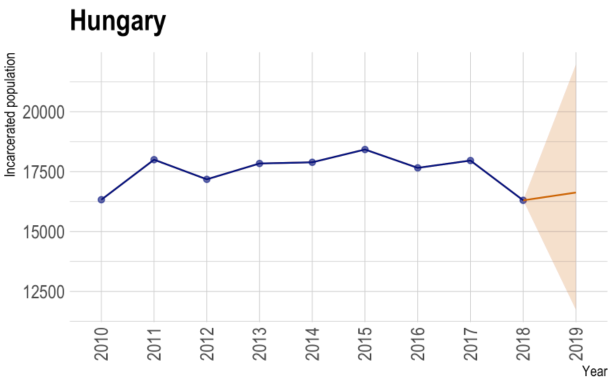
Hungary
Incarcerated population
20000
17500
15000
12500
2010
2011
2012
2013
2014
2015
2016
2017
2018
2019
Year

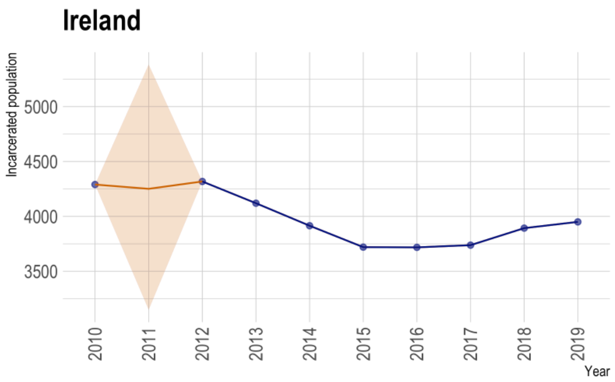
Ireland
Incarcerated population
5000
4500
4000
3500
2010
2011
2012
2013
2014
2015
2016
2017
2018
2019
Year

Israel

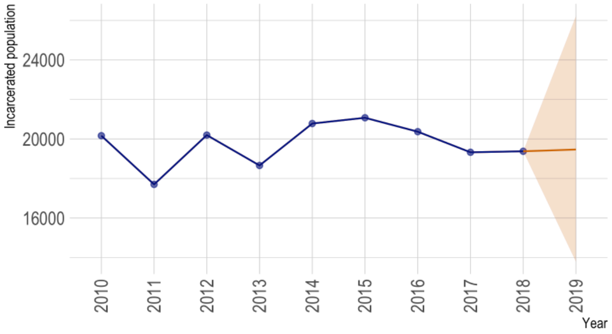

Kazakhstan

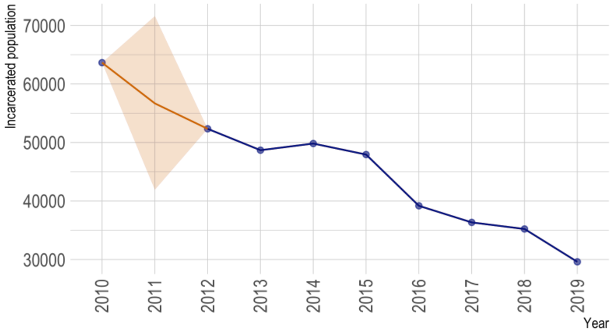

Kyrgyzstan

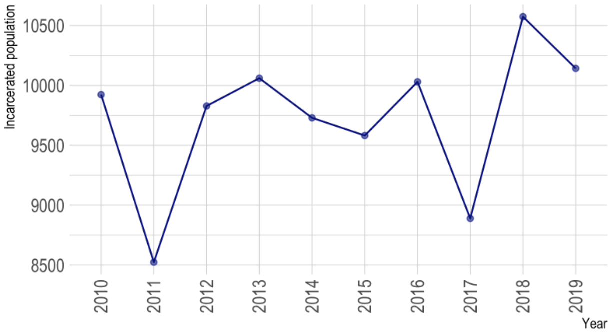

Latvia

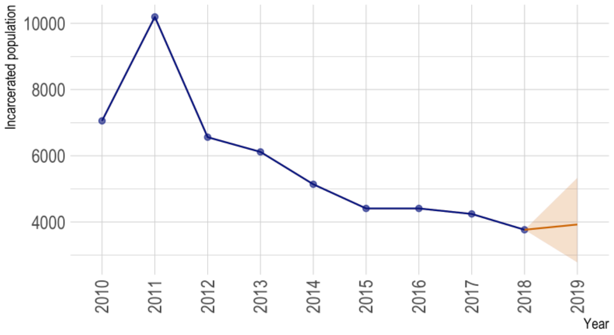

Lithuania

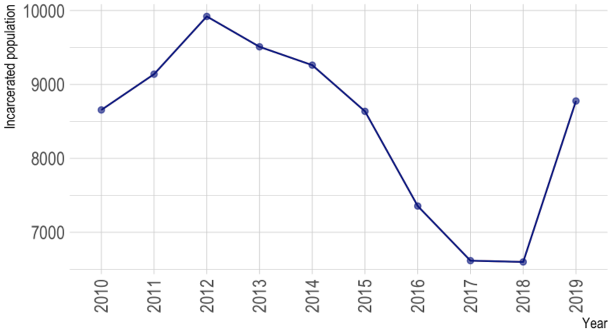

Luxembourg

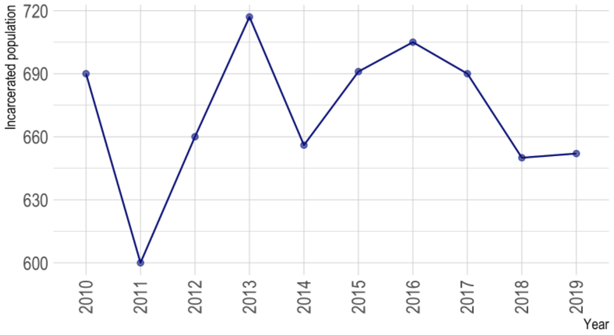

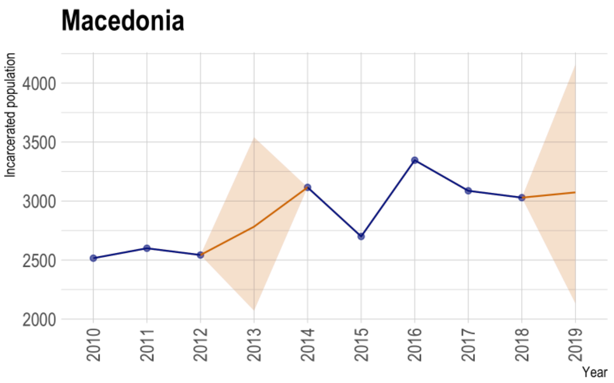
Macedonia
Incarcerated population
4000
3500
3000
2500
2000
2010 2011 2012 2013 2014 2015 2016 2017 2018 2019
Year

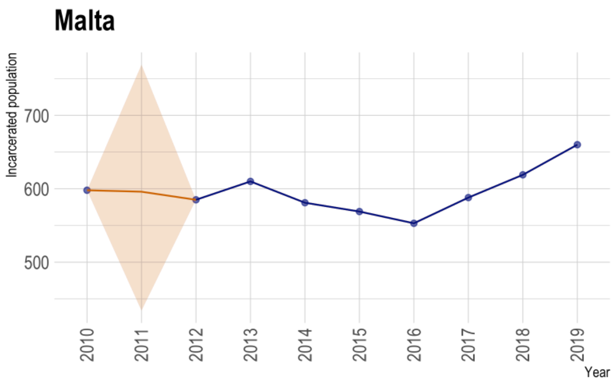
Malta
Incarcerated population
700
600
500
2010 2011 2012 2013 2014 2015 2016 2017 2018 2019
Year

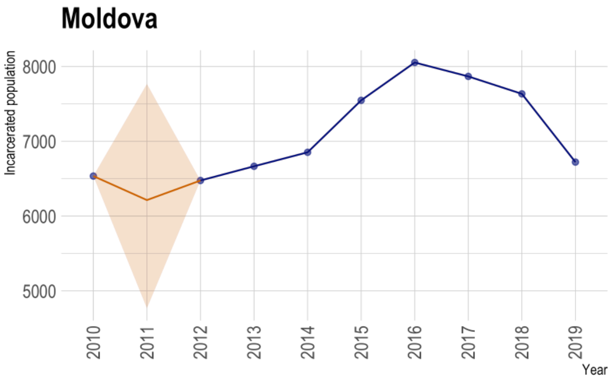
Moldova
Incarcerated population
8000
7000
6000
5000
2010 2011 2012 2013 2014 2015 2016 2017 2018 2019
Year

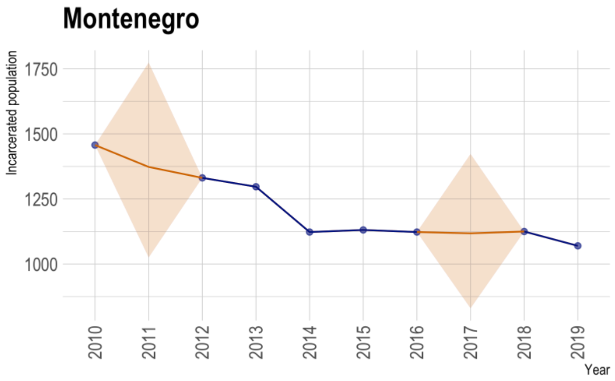
Montenegro
Incarcerated population
1750
1500
1250
1000
2010 2011 2012 2013 2014 2015 2016 2017 2018 2019
Year

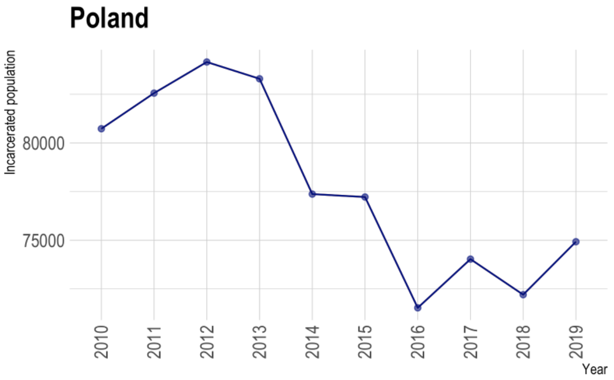
Poland
Incarcerated population
80000
75000
2010 2011 2012 2013 2014 2015 2016 2017 2018 2019
Year

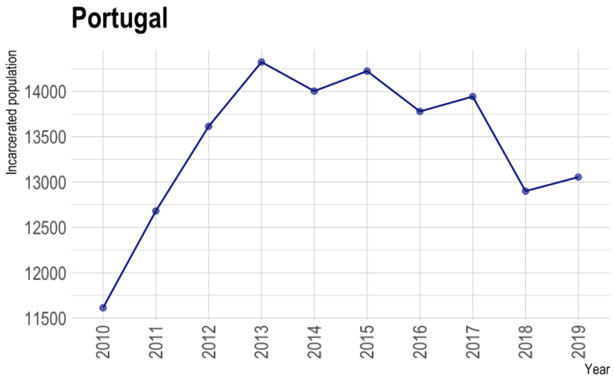
Portugal
Incarcerated population
14000
13500
13000
12500
12000
11500
2010 2011 2012 2013 2014 2015 2016 2017 2018 2019
Year

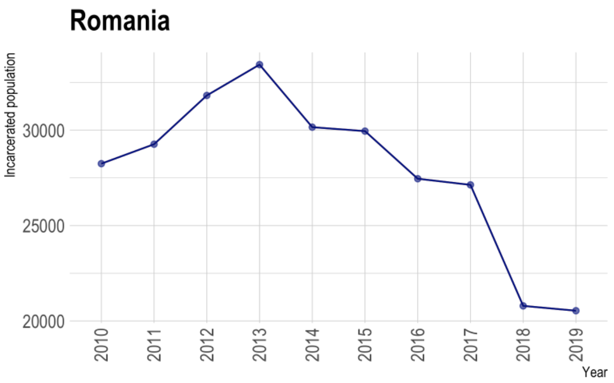
Romania
Incarcerated population
30000
25000
20000
2010
2011
2012
2013
2014
2015
2016
2017
2018
2019
Year

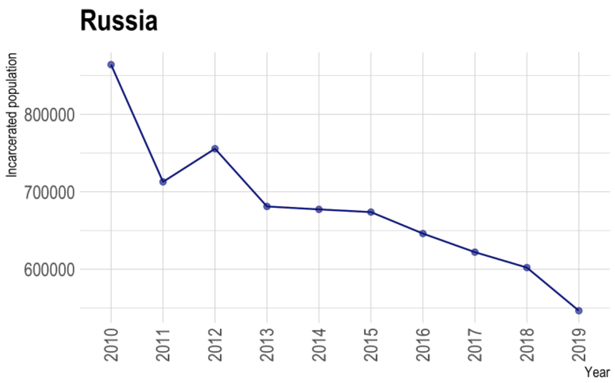
Russia
Incarcerated population
800000
700000
600000
2010
2011
2012
2013
2014
2015
2016
2017
2018
2019
Year

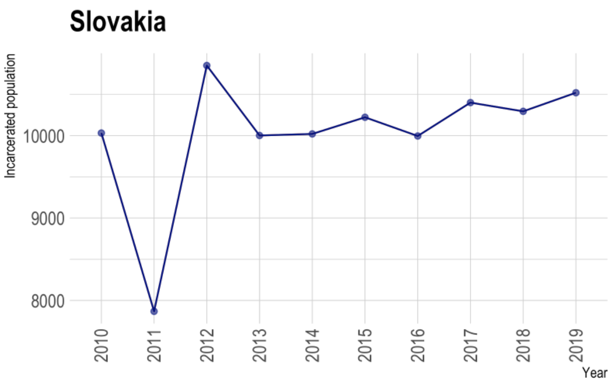
Slovakia
Incarcerated population
10000
9000
8000
2010
2011
2012
2013
2014
2015
2016
2017
2018
2019
Year

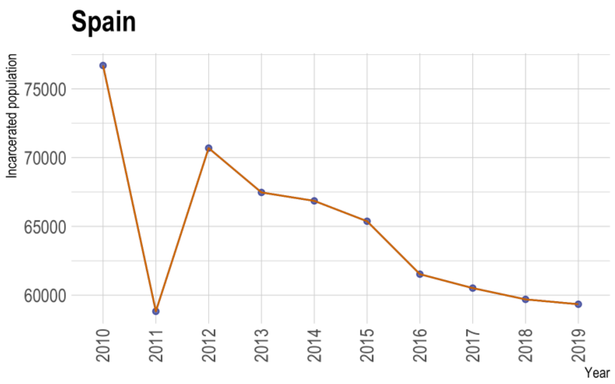
Spain
Incarcerated population
75000
70000
65000
60000
2010
2011
2012
2013
2014
2015
2016
2017
2018
2019
Year

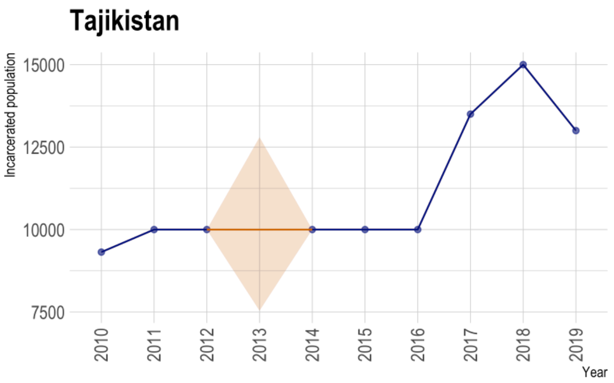
Tajikistan
Incarcerated population
15000
12500
10000
7500
2010
2011
2012
2013
2014
2015
2016
2017
2018
2019
Year

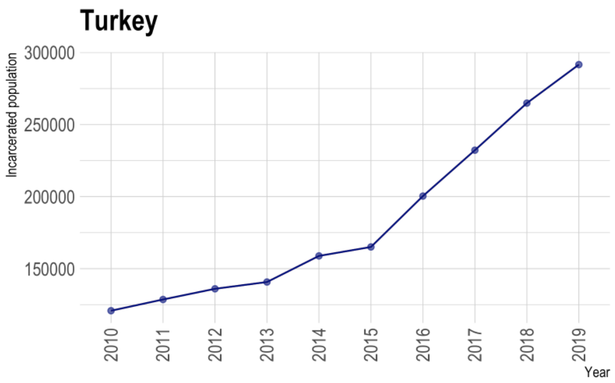
Turkey
Incarcerated population
300000
250000
200000
150000
2010
2011
2012
2013
2014
2015
2016
2017
2018
2019
Year

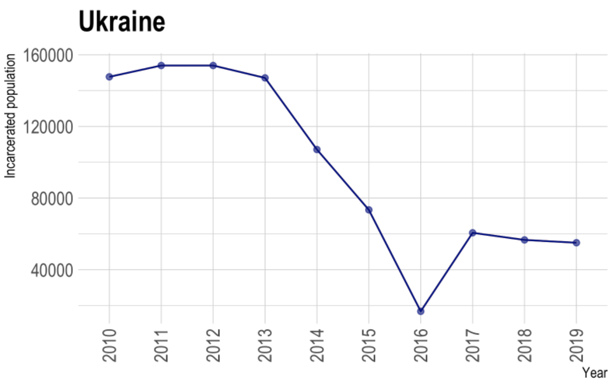
Ukraine
Incarcerated population
160000
120000
80000
40000
2010
2011
2012
2013
2014
2015
2016
2017
2018
2019
Year

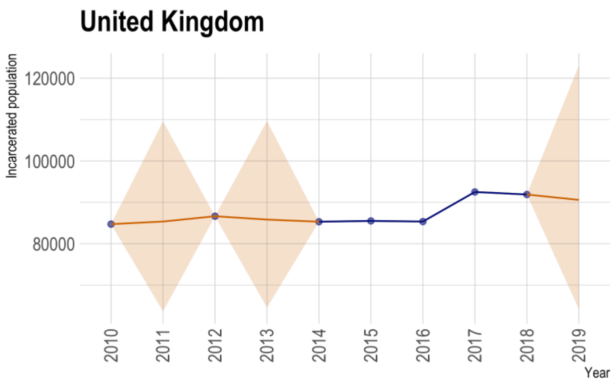
United Kingdom
Incarcerated population
120000
100000
80000
2010
2011
2012
2013
2014
2015
2016
2017
2018
2019
Year

Supplement Section 3: Outcomes

Supplemental Table S1. *Characteristics of countries included vs. not included to fit the proposed joint model*

**Characteristic**[(a)] [n (%); Median (IQR)]	**Included**[(b)] N = 63	**Not included**[(b)] N = 36
WHO Region		
Americas	25 (39.7)	20 (55.6)
Europe	38 (60.3)	16 (44.4)
Incarceration rate (number of people in prison per 100,000 people in the national population	191.0 (152.0)	149.5 (272.0)
Missing	8 (12.7)	14 (38.9)
Percent (%) capacity of prisons	102.5 (51.0)	83.0 (35.5)
Missing	24 (38.1)	21 (58.3)
Percent (%) of incarcerated population who are pre-trial detainees	28.3 (22.2)	27.4 (11.2)
Missing	24 (38.1)	24 (66.7)
Percent (%) of incarcerated population who are female	5.4 (3.4)	3.8 (2.7)
Missing	25 (39.7)	21 (58.3)
National tuberculosis case notifications per 100,000 people	20.5 (32.4)	4.7 (3.7)
Missing	3 (4.8)	3 (8.3)
Estimated national tuberculosis incidence per 100,000 people	26.0 (38.8)	5.6 (6.0)
Missing	2 (3.2)	2 (5.6)

(a) *Characteristics based on 2018 values for all countries*
(b) *Countries with >3 years of data on tuberculosis in prisons were included in the final model*

Supplemental Table S2. *Total number of people who are incarcerated by region in 2018*

**Region**	**Total Number of People Who are Incarcerated**
Africa	1,162,440
Americas	3,787,059
Asia	4,164,323
Europe	1,555,643
Oceania	64,154
Total	10,743,619

Supplemental Table S3. *Reporting of national tuberculosis notification data among people who are incarcerated by country and years of data.*

Country	Years of Data
Albania	2010-2012, 2014-2019, 2022
Andorra	2014-2019, 2022
Antigua and Barbuda	2010, 2012, 2014, 2016-2022
Argentina	2014-2022
Armenia	2010, 2011, 2014-2022
Austria	2012, 2014, 2016, 2018
Azerbaijan	2010, 2011, 2014-2022
Bahamas	2014-2022
Belarus	2010, 2015-2019
Belgium	2010, 2014-2022
Belize	2010, 2012, 2014, 2018-2022
Bolivia	2010, 2012-2016, 2018-2022
Bosnia and Herzegovina	2014-2016, 2018
Brazil	2010-2022
Bulgaria	2010-2012, 2014-2022
Chile	2012-2022
Costa Rica	2012-2019, 2021, 2022
Czech Republic	2010-2012, 2014-2022
Denmark	2010, 2012, 2014-2017, 2019
Dominican Republic	2011-2022
Ecuador	2014-2022
El Salvador	2010-2022
Estonia	2010-2012, 2014-2022
Finland	2010, 2012, 2015, 2016, 2018
France	2011, 2016-2018, 2021, 2022
Georgia	2010-2012, 2014-2022
Greece	2014-2018
Guatemala	2011, 2017-2022
Guyana	2010-2022
Haiti	2010-2018, 2020-2022
Honduras	2010-2022
Hungary	2011, 2012, 2014, 2016, 2018, 2019
Ireland	2012 - 2022
Israel	2010-2012, 2014-2016
Jamaica	2015-2018, 2020
Kazakhstan	2012, 2014, 2015, 2017-2022
Kyrgyzstan	2011, 2012, 2014-2019, 2022
Latvia	2010-2012, 2014-2017
Lithuania	2010-2012, 2014-2021
Luxembourg	2011, 2012, 2014-2016, 2018-2022

Macedonia	2010-2012, 2014-2017
Malta	2014-2016, 2018
Mexico	2012-2022
Moldova	2010-2012, 2014-2022
Montenegro	2010, 2012, 2014, 2015, 2018, 2019
Nicaragua	2014, 2016, 2018-2022
Panama	2010, 2012, 2014, 2016, 2018, 2021, 2022
Paraguay	2012, 2014-2022
Peru	2010-2022
Poland	2010-2012, 2014-2022
Portugal	2011, 2012, 2014-2022
Romania	2010-2022
Russia	2011, 2012, 2014-2022
Slovakia	2010, 2011, 2014-2022
Spain	2010-2017
Suriname	2016-2020
Tajikistan	2010-2012, 2014-2022
Turkey	2010-2012, 2014-2022
Ukraine	2010-2012, 2014-2022
United Kingdom	2011, 2012, 2014-2018, 2022
United States of America	2010, 2012-2022
Uruguay	2010-2022
Venezuela	2010-2022

Supplemental Table 4. *Observed, Predicted and 95% credible intervals for tuberculosis notified case in prisons, and prison population, stratified by key subgroups, from 2020 - 2022*

	**2020**				**2021**				**2022**			
	**TB Cases**		**Prison Population**		**TB Cases**		**Prison Population**		**TB Cases**		**Prison Population**	
	**Observed**	**Predicted**	**Observed**	**Predicted**	**Observed**	**Predicted**	**Observed**	**Predicted**	**Observed**	**Predicted**	**Observed**	**Predicted**
**All Included Countries Estimate**	23074	29126.1 (21264.1,238362.2)	2540676	2528162.0 (22259779.0,2888383.0)	21168	3100.2 (2033.1,44236.9)	2560322	2527642.0 (2161570.0,2944932.0)	22552	33587.1 (20221.0,50616.3)	2666243	2526064.5 (2100754.0,3002290.0)
**Sub-Regional Estimate**												
Central/North America	2013	2973.1 (1917.5,4457.7)	327861	312007.5 (247247.0,381154.0)	1659	3323.4 (1763.4,5624.4)	333774	311810.5 (239755.0,702107.0)	2522	3698.8 (1736.9,7198.3)	373299	311039.0 (230316.0,414449.0)
South America	14205	16578.0 (10456.1,23996.0)	1134874	1059616.0 (837855.0,1309361.0)	13247	17822.1 (9851.6,28688.2)	1162336	1055779.0 (799917.0,1365773.0)	14103	19493.9 (9427.7,33357.8)	1210154	1052207.0 (754574.0,1400560.0)
Western Europe	36	60.6 (33.6,96.0)	26144	28538.0 (23225.0,34676.0)	41	61.3 (27.6,111.3)	26347	28672.5 (22343.0,36291.0)	33	61.7 (22.7,126.0)	27579	28733.0 (21528.0,37828.0)
Eastern Europe	6820	8986.2 (5123.1,14216.9)	1051797	1119044.0 (920793.0,1330115.0)	6821	9066.0 (4278.5,16255.9)	1037865	1117105.0 (891423.0,1391588.0)	5894	9180.0 (3618.7,18027.9)	1055211	1113206.0 (855813.0,1424025.0)
**Tuberculosis Burden Among the General Population**												
Low	307	475.1 (321.8,652.2)	433277	458398.5 (3790957.0,554457.0)	278	468.2 (286.0,713.3)	467158	457658.5 (357555.0,582358.0)	394	462.4 (241.9,762.6)	467158	455971.0 (338734.0,591175.0)
Medium	18003	21334.4 (14263.1,30278.6)	18606568	1754654.5 (1459974.0,2080602.0)	16757	22444.3 (13233.4,35216.1)	1793063	1751886.5 (1408647.0,2146564.0)	17295	23961.3 (12788.3,40490.6)	1793063	1479171.0 (1368185.0,2221173.0)
High	4764	7102.3 (4792.5,9833.4)	300831	310482.5 (271421.0,351755.0)	4133	7829.9 (4564.6,11998.8)	300101	311995.5 (264709.0,363196.0)	4863	8641.2 (4414.1,14372.7)	300101	3312504.5 (259125.0,372125.0)
**Crowding Level in Prisons**												
No Overcrowding	6741	8819.9 (4917.0,13988.2)	943003	978287.5 (793882.0,1184819.0)	6134	8897.0 (4304.6,16336.4)	904041	977107.5 (766988.5,1243301.0)	5988	9032.0 (6746.6,18176.3)	909522	975752.0 (721444.0,1263051.0)
100% – 120% Overcrowding	279	412.0 (295.2,553.9)	112081	118515.5 (101319.0,139094.0)	323	418.4 (262.2,604.6)	112952	119155.0 (98012.0,142623.0)	394	423.1 (245.0,669.9)	118116	119467.5 (95988.0,147723.0)
>120% - 200% Overcrowding	11832	13424.1 (7821.7,20510.1)	1275669	1209141.0 (971898.0,1471860.0)	11278	14399.1 (7076.7,24673.4)	1339649	1204736.0 (926161.0,1534509.0)	11715	15744.7 (6466.0,28825.7)	1394838	1202080.0 (894247.0,1585065.0)
>200% Overcrowding	4154	5748.2 (3651.3,8420.5)	194858	195720.5 (162250.0,231434.0)	3382	6327.1 (3284.0,10227.1)	187850	196158.0 (155748.0,240949.0)	4383	6941.4 (3030.8,12233.2)	225481	196151.0 (152588.0,249061.0)

Supplemental Table S5. *Percent Difference between the observed and predicted tuberculosis case rate per 100, 000 stratified by key subgroups*

	2020	2021	2022
	**TB Case Rate per 100,000 people**	**TB Case Rate per 100,000 people**	**TB Case Rate per 100,000 people**
**All Included Countries Estimate**	-12.5 (-74.9,28.2)	-34.2 (-130.9,25.3)	-49.4 (-162.4,22.6)
**Sub-Regional Estimates**			
Central/North America	-21.2 (-134.5,36.8)	-59.7 (-28.1.2,30.1)	-61.7 (-305.0,41.4)
South America	-21.4 (-123.1,35.8)	-45.5 (-227.3,36.1)	-53.8 (-263.3,41.4)
Western Europe	-52.8 (-199.7,32.4)	-54.2 (-281.0,45.6)	-32.7 (-250.3,54.0)
Eastern Europe	-19.2 (-132.0,40.4)	-27.9 (-185.3,48.2)	-38.8 (-254.4,53.6)
**Tuberculosis Burden Among the General Population**[1]			
Low	-32.4 (-137.2, 27.3)	-48.4 (-196.9,26.4)	-20.9 (-137.6, 41.9)
Medium	-18.0 (-100.9,32.1)	-32.9 (-159.8,36.7)	-40.4 (-199.9,41.5)
High	-39.7 (-118.4,15.5)	-71.8 (-201.4,9.9)	-82.8 (-264.7,22.5)
**Crowding Level in Prisons**			
No Overcrowding	-8.9 (-118.1,49.9)	-20.9 (-193.4,52.2)	-30.8 (-257.4,59.6)
100% – 120% Overcrowding	-38.2 (-119.4,15.4)	-17.9 (-103.8,35.1)	-9.7 (-105.9,47.5)
>120% - 200% Overcrowding	-16.2 (-121.6,41.4)	-37.3 (-210.2,49.0)	-48.5 (-274.1,49.3)
>200% Overcrowding	-31.4 (-127.2,27.8)	-65.7 (-22.5,29.8)	-63.7 (-290.9,41.7)

[1] Low: 0-20 cases per 100,00 people; Medium: ≥20 - ≤50 cases per 100,000 people; High: >50 cases per 100,000 people

Note: Subgroup estimates will not sum up to the global estimate as these are percent differences between the observed and the predicted

Key	*-90.0%- -80.0%*	*-70.0%- -60.0%*	*-50.0%- -40.0%*	*-30.0% - -20.0%*	*-10.0 - <0%*

Supplemental Table S6. *Percent Difference between the observed and predicted tuberculosis notifications, notification rate, and prison population stratified by WHO Region and tuberculosis burden in prisons*

	2020			2021			2022		
	**TB Cases**	**TB Case Rate per 100,000 people**	**Prison Population**	**TB Cases**	**TB Case Rate per 100,000 people**	**Prison Population**	**TB Cases**	**TB Case Rate per 100,000 people**	**Prison Population**
**All Included Countries Estimate**	-26.2 (-66.3,7.8)	-12.5 (-74.9,28.2)	0.5 (-13.7,12.4)	-46.4 (-108.9,3.9)	-34.2 (-130.9,25.3)	1.3 (-15.0,15.6)	-48.9 (-124.4,10.3)	-49.4 (-162.4,22.6)	5.3 (-12.6,21.2)
**Regional Estimates**									
Americas	-18.9 (-64.7,17.4)	-8.5 (-86.0,37.9)	-9.5 (-33.0,11.4)	-39.9 (-115.5,14.0)	-33.5 (-165.0,35.1)	-4.8 (-32.7,18.7)	-37.4 (-123.1,24.1)	-57.7 (-226.9,30.9)	12.9 (-9.9 31.7)
Europe	-27.1 (-102.9,24.8)	-19.4 (-128.8,40.6)	-6. 5 (-26.7,11.3)	-38.7 (-146.2,30.4)	-28.6 (-180.7,46.9)	-7.9 (-31.2,12.3)	-47.0 (-184.0,37.4)	-38.6 (-237.9,52.1)	-6.0 (-30.5,15.9)
**Tuberculosis Burden in Prisons**[1]									
Low	-55.8 (-134.2, 5.1)	-30.0 (-163.3,39.2)	-19.9 (-56.0,11.1)	-58.4 (-159.2, 9.1)	-41.6 (-227.9,40.5)	-11.9 (-52.7,20.9)	-10.6 (-106.9,50.0)	-16.4 (-193.6,59.6)	5.0 (-23.9,29.5)
Medium	-17.6 (-59.5,17.1)	-17.8 (-88.9,29.0)	0.2 (-16.7,15.6)	-9.0 (-60.8,32.0)	-11.4 (-102.8,41.8)	2.2 (-16.7,20.7)	30.2 (-15.3,60.7)	25.6 (-57.6, 65.9)	6.2 (-15.3,26.9)
High	-23.4 (-63.5,10.4)	-24.0 (-97.4,22.6)	1.3 (-15.8,17.2)	-43.2 (-106.2,7.1)	-43.5 (-156.1,23.4)	0.2 (-21.3,19.5)	-47.5 (-124.1,12.1)	-53.5 (-194.6,27.3)	4.0 (-20.8,23.9)

[1]Low: 0-100 cases per 100,00 people; Medium: ≥100 - ≤500 cases per 100,000 people; High: >500 cases per 100,000 people

Note: Subgroup estimates will not sum up to the global estimate as these are percent differences between the observed and the predicted

Key	*-50.0%- -40.0%*	*-30.0% - -20.0%*	*-10.0 - <0%*	*0% - 20%*	*>20%*

Supplemental Figure S1. *Scatterplot comparing the percent difference in observed vs. predicted prison population stratified by region in A) 2020 vs. 2021, B) 2021 vs. 2022, and C) 2020 vs. 2022. To be included countries had to have data from 2020-2022, i.e., all three years the pandemic.*

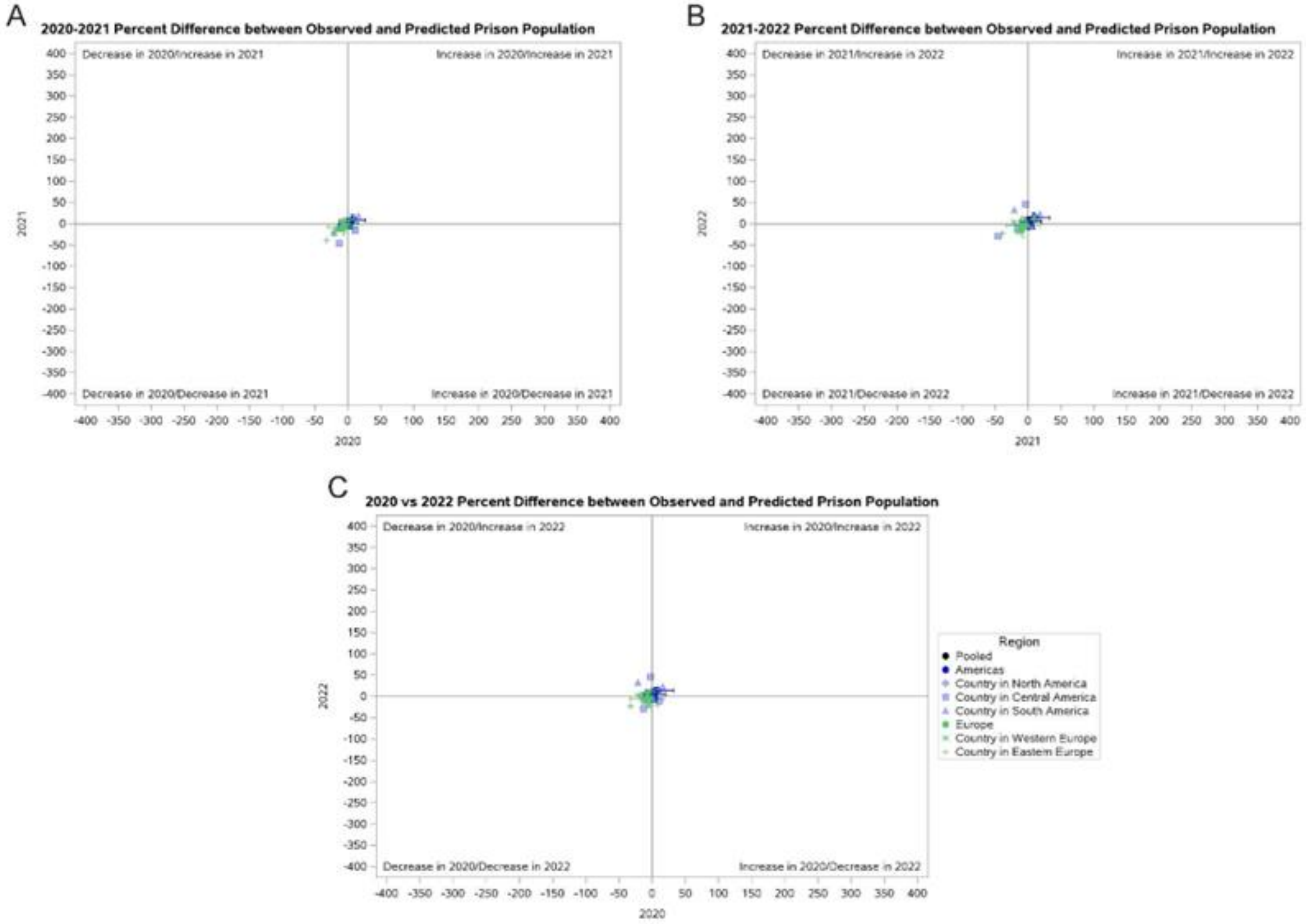

Supplemental Figure S2. *Scatterplot comparing the percent difference in observed vs. predicted tuberculosis case notifications stratified by tuberculosis burden among the general population in A) 2020 vs. 2021, B) 2021 vs. 2022, and C) 2020 vs. 2022. To be included countries had to have data from 2020-2022, i.e., all three years of the pandemic.*

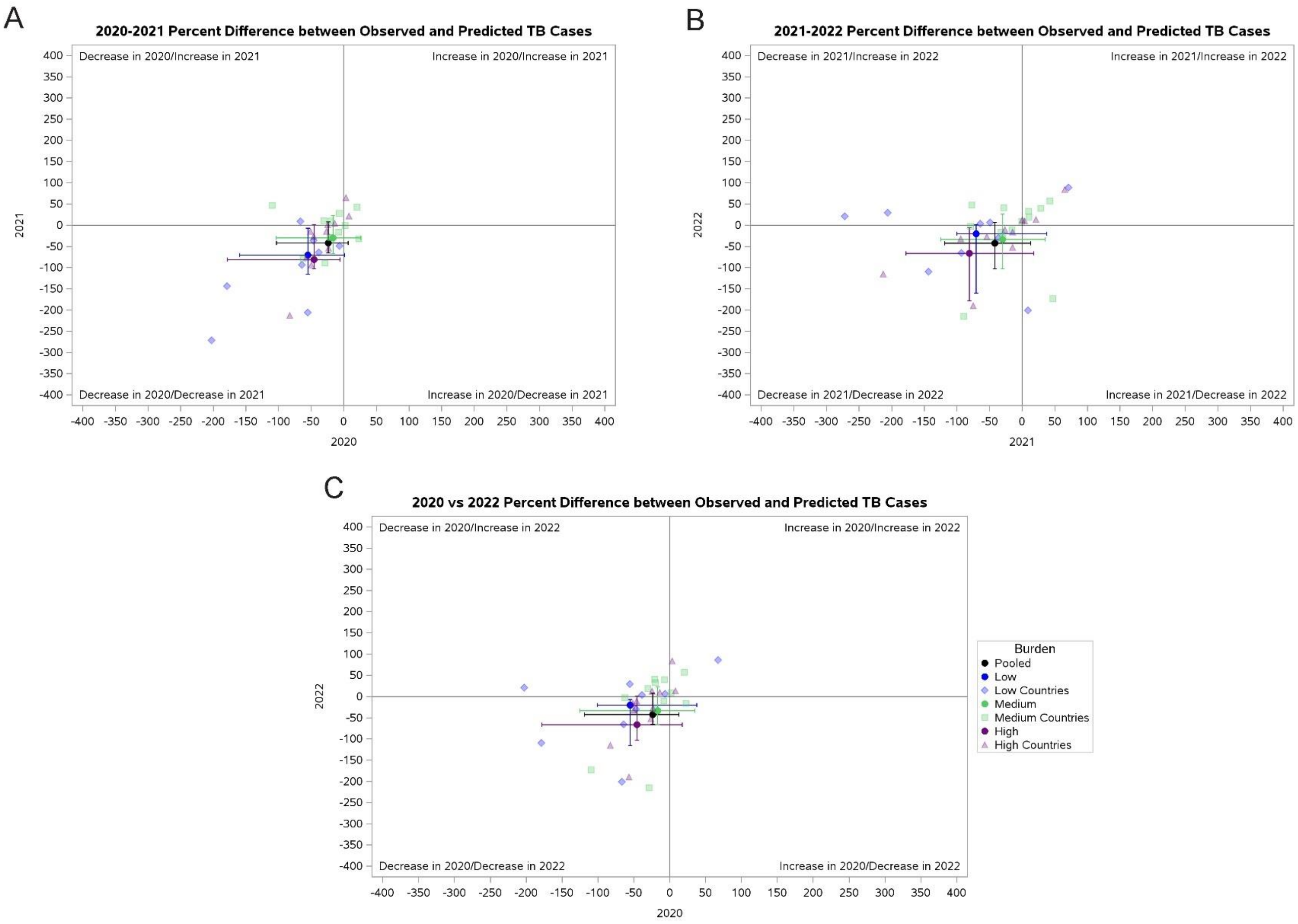

Supplemental Figure S3. *Scatterplot comparing the percent difference in observed vs. predicted tuberculosis case notifications stratified by prison crowding in A) 2020 vs. 2021, B) 2021 vs. 2022, and C) 2020 vs. 2022. To be included countries had to have data from 2020-2022, i.e., all three years of the pandemic.*

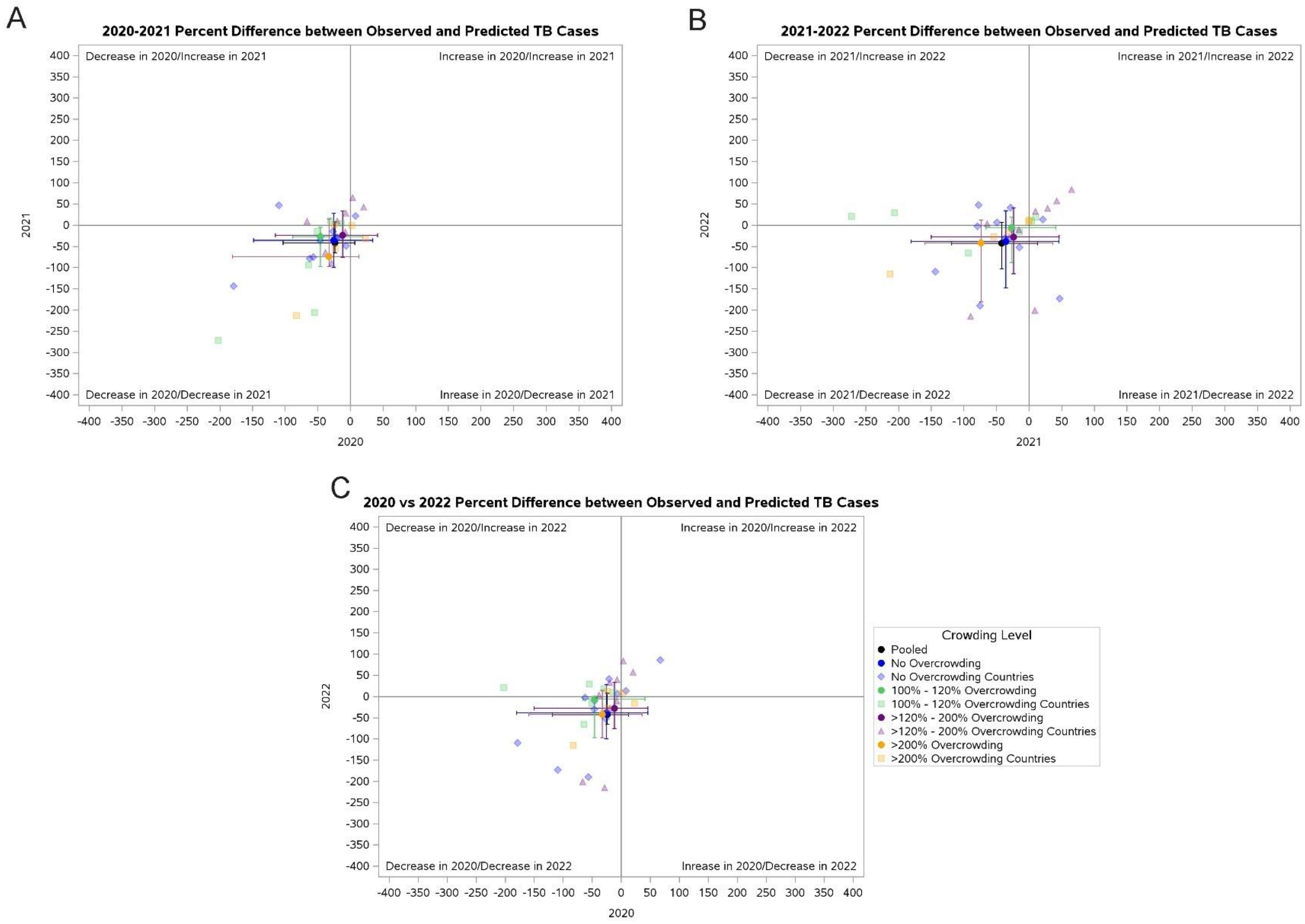

Supplemental Figure S4. *Scatterplot comparing the percent difference in observed vs. predicted tuberculosis case notifications stratified by tuberculosis notification rate among the prison population in A) 2020 vs. 2021, B) 2021 vs. 2022, and C) 2020 vs. 2022. To be included countries had to have data from 2020-2022, i.e., all three years of the pandemic.*

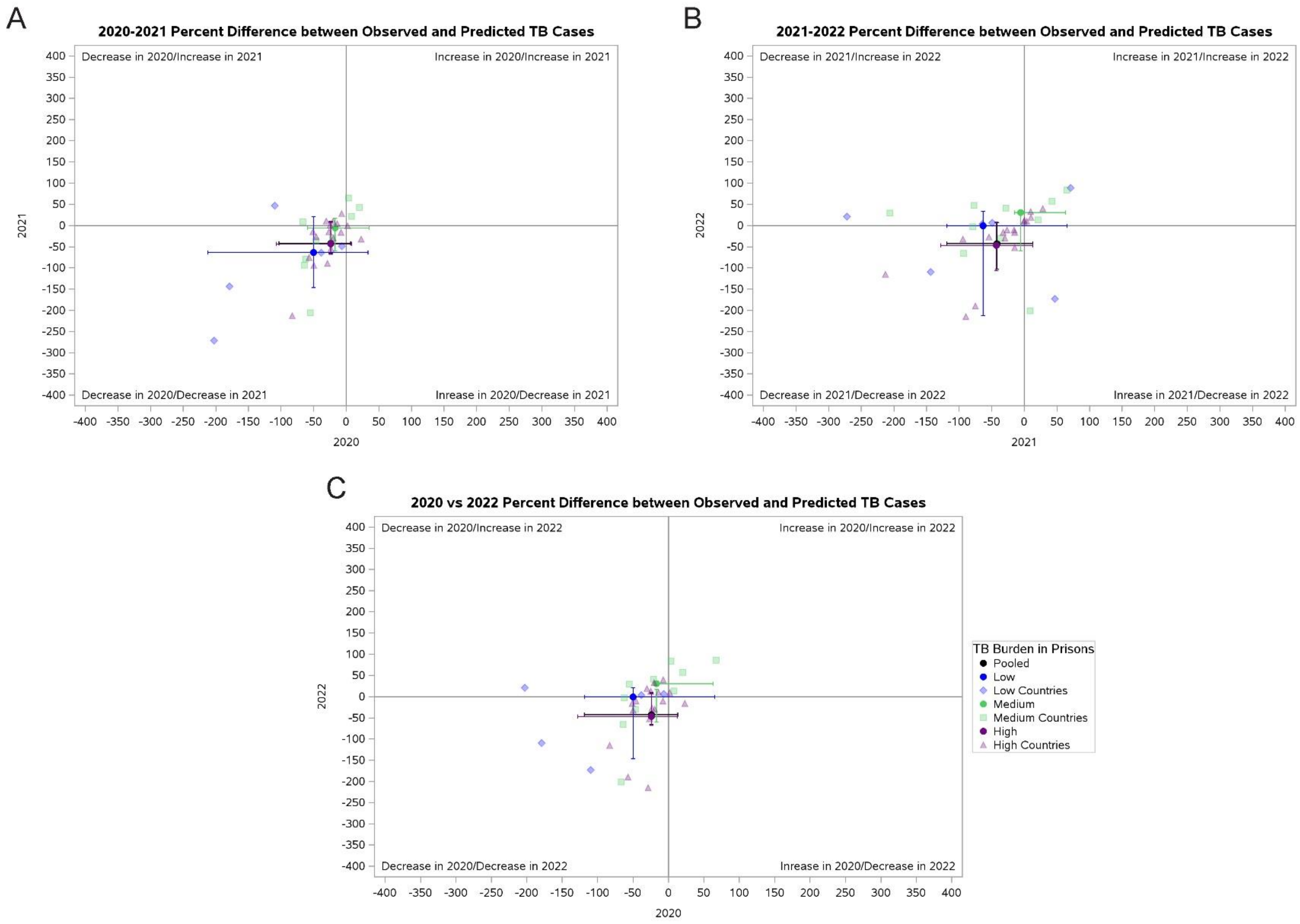

Supplemental Figure S5. *Scatterplot comparing the percent difference in observed vs. predicted tuberculosis case notification rate stratified by tuberculosis burden among the general population in A) 2020 vs. 2021, B) 2021 vs. 2022, and C) 2020 vs. 2022. To be included countries had to have data from 2020-2022, i.e., all three years of the pandemic.*

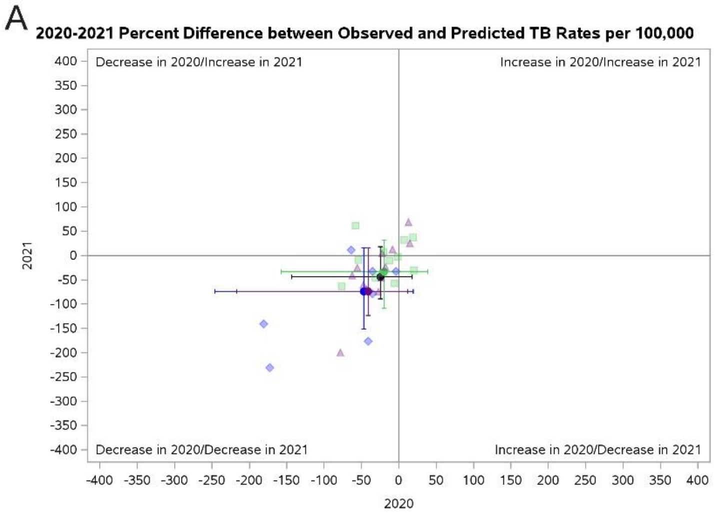

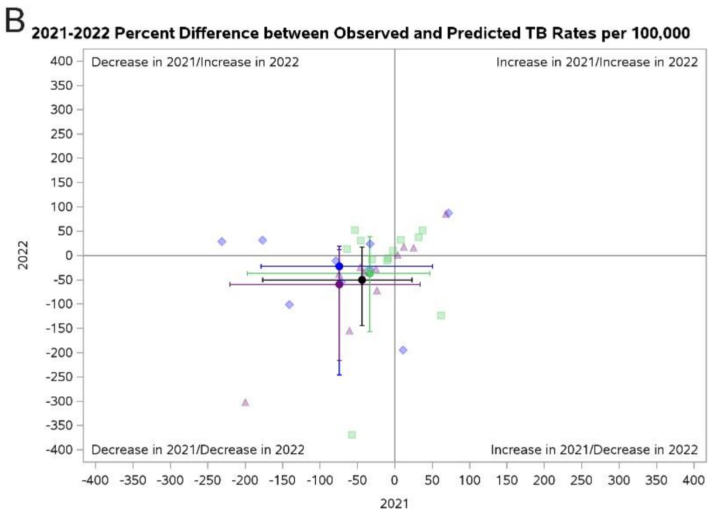

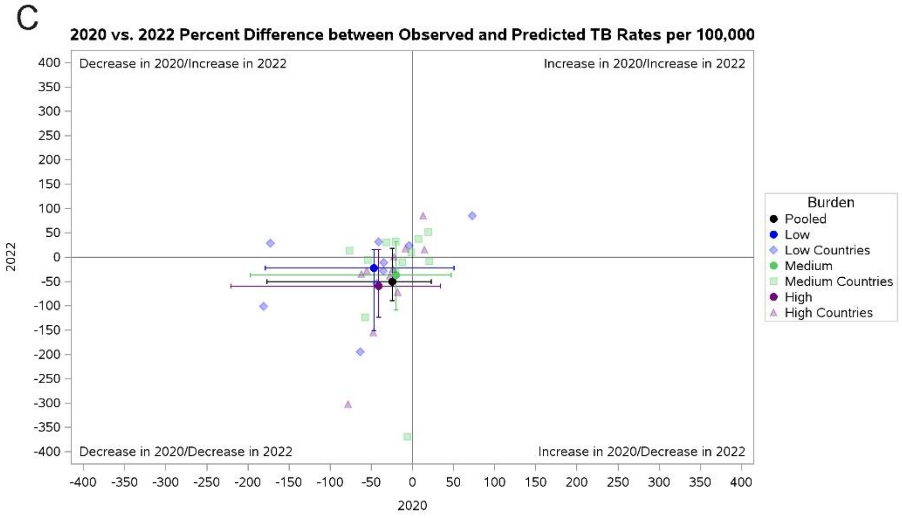

Supplemental Figure S6. *Scatterplot comparing the percent difference in observed vs. predicted tuberculosis case notification rate stratified by prison crowding in A) 2020 vs. 2021, B) 2021 vs. 2022, and C) 2020 vs. 2022. To be included countries had to have data from 2020-2022, i.e., all three years of the pandemic.*

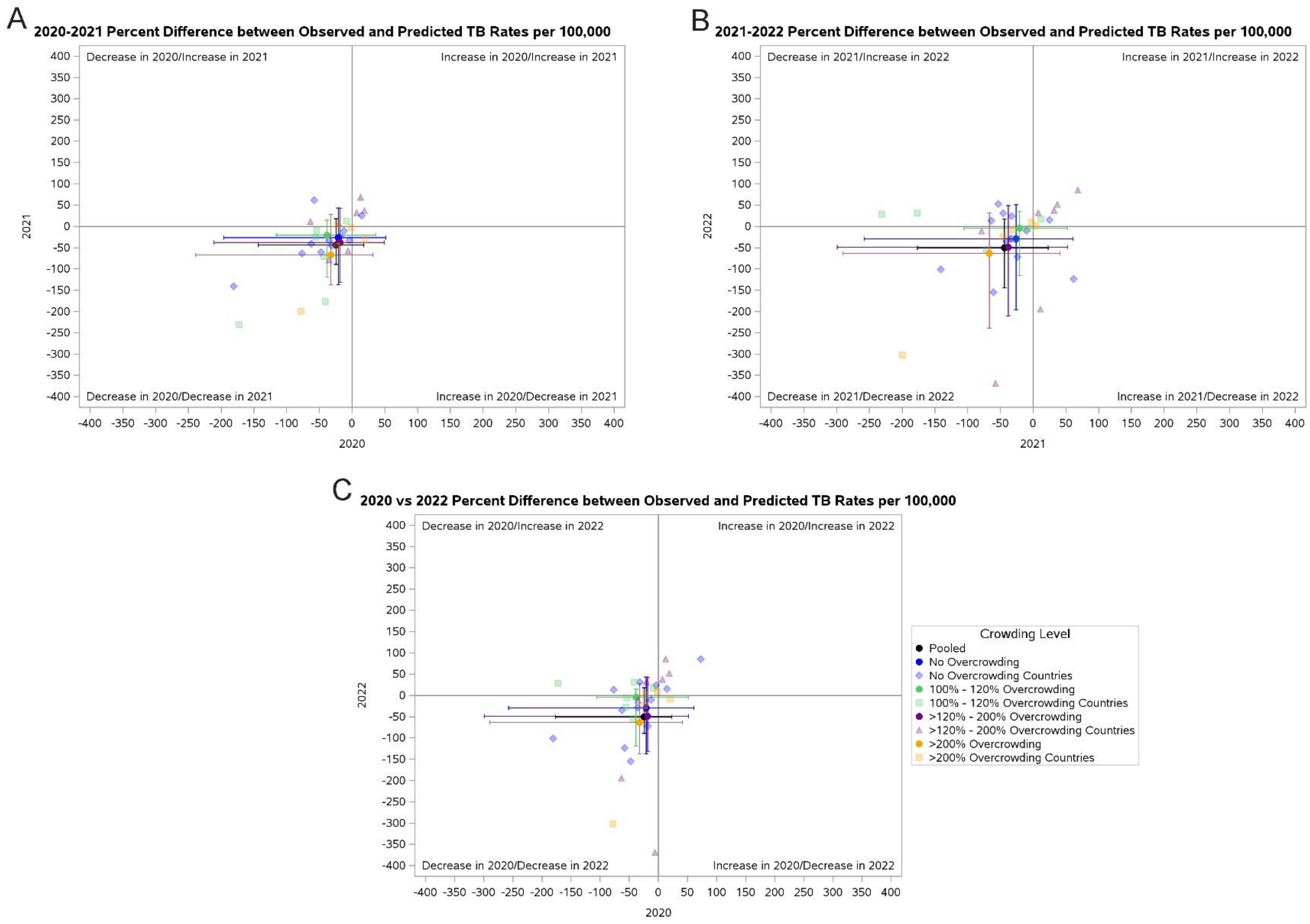

Supplemental Figure S7. *Scatterplot comparing the percent difference in observed vs. predicted tuberculosis case notification rate stratified by tuberculosis notification rate among the prison population in A) 2020 vs. 2021, B) 2021 vs. 2022, and C) 2020 vs. 2022. To be included countries had to have data from 2020-2022, i.e., all three years of the pandemic.*

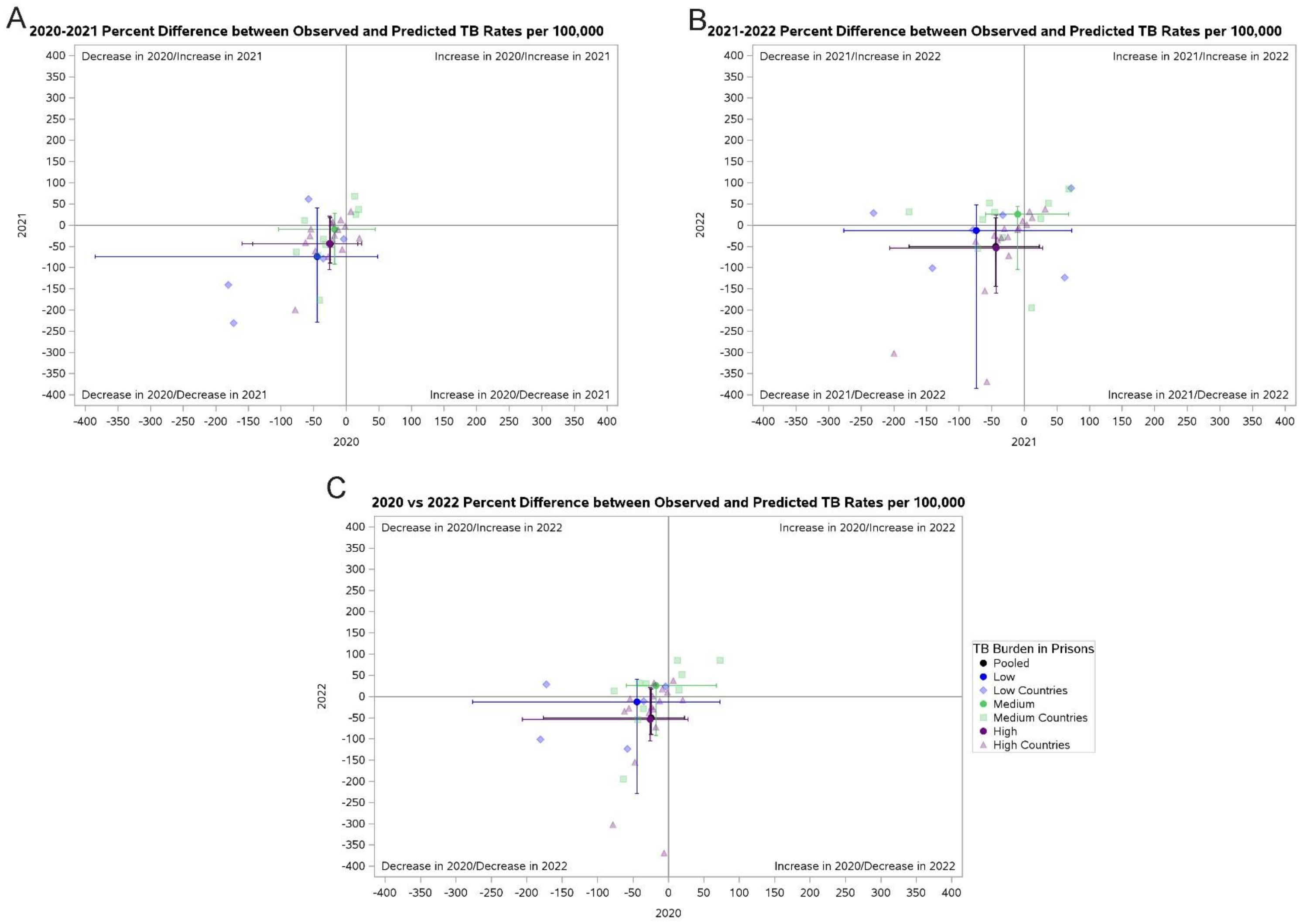

Supplemental Figure S8. *Scatterplot comparing the percent difference in observed vs. predicted prison population stratified by tuberculosis burden among the general population in A) 2020 vs. 2021, B) 2021 vs. 2022, and C) 2020 vs. 2022. To be included countries had to have data from 2020-2022, i.e., all three years of the pandemic.*

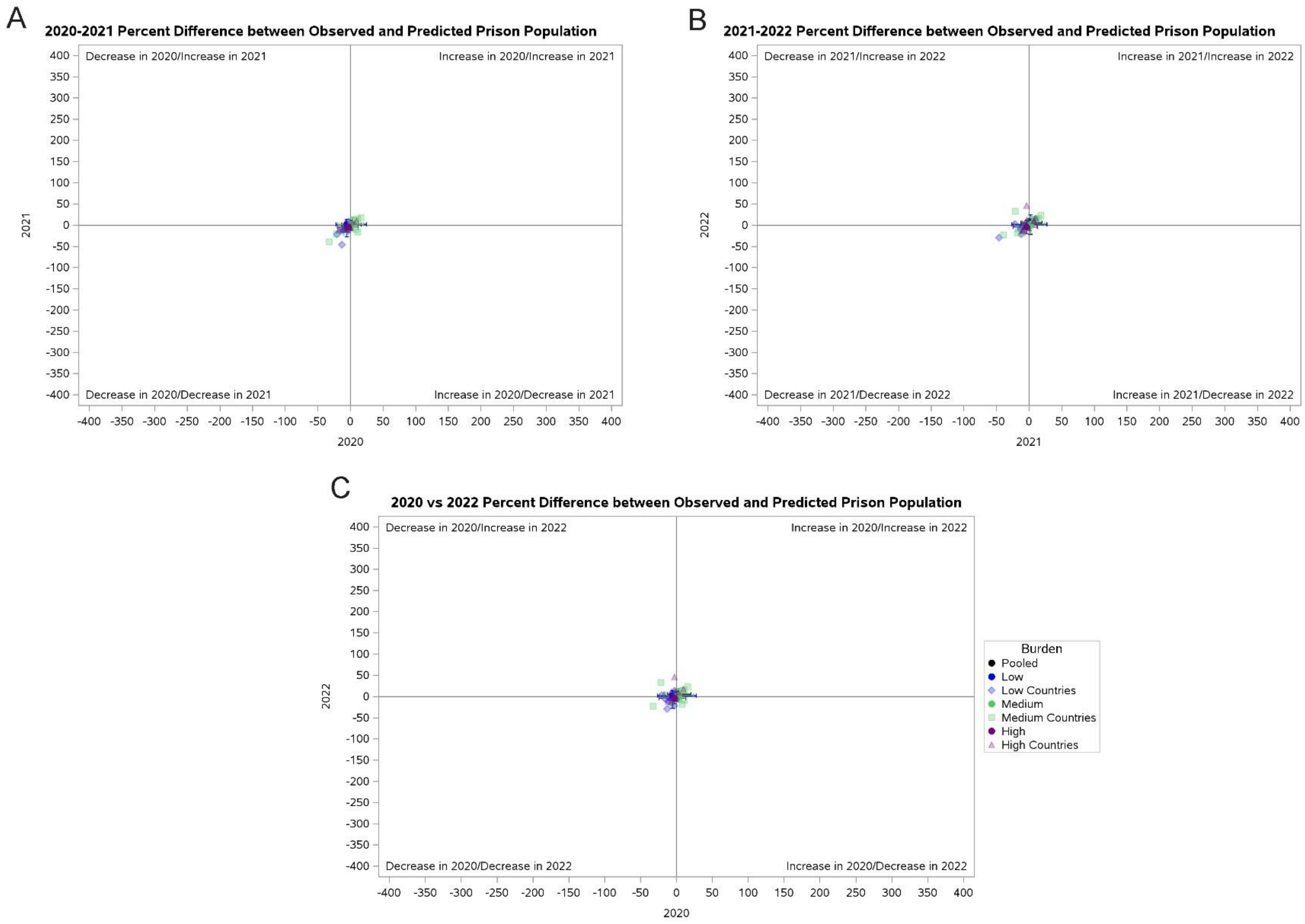

Supplemental Figure S9. *Scatterplot comparing the percent difference in observed vs. predicted prison population stratified by prison crowding in A) 2020 vs. 2021, B) 2021 vs. 2022, and C) 2020 vs. 2022. To be included countries had to have data from 2020-2022, i.e., all three years of the pandemic.*

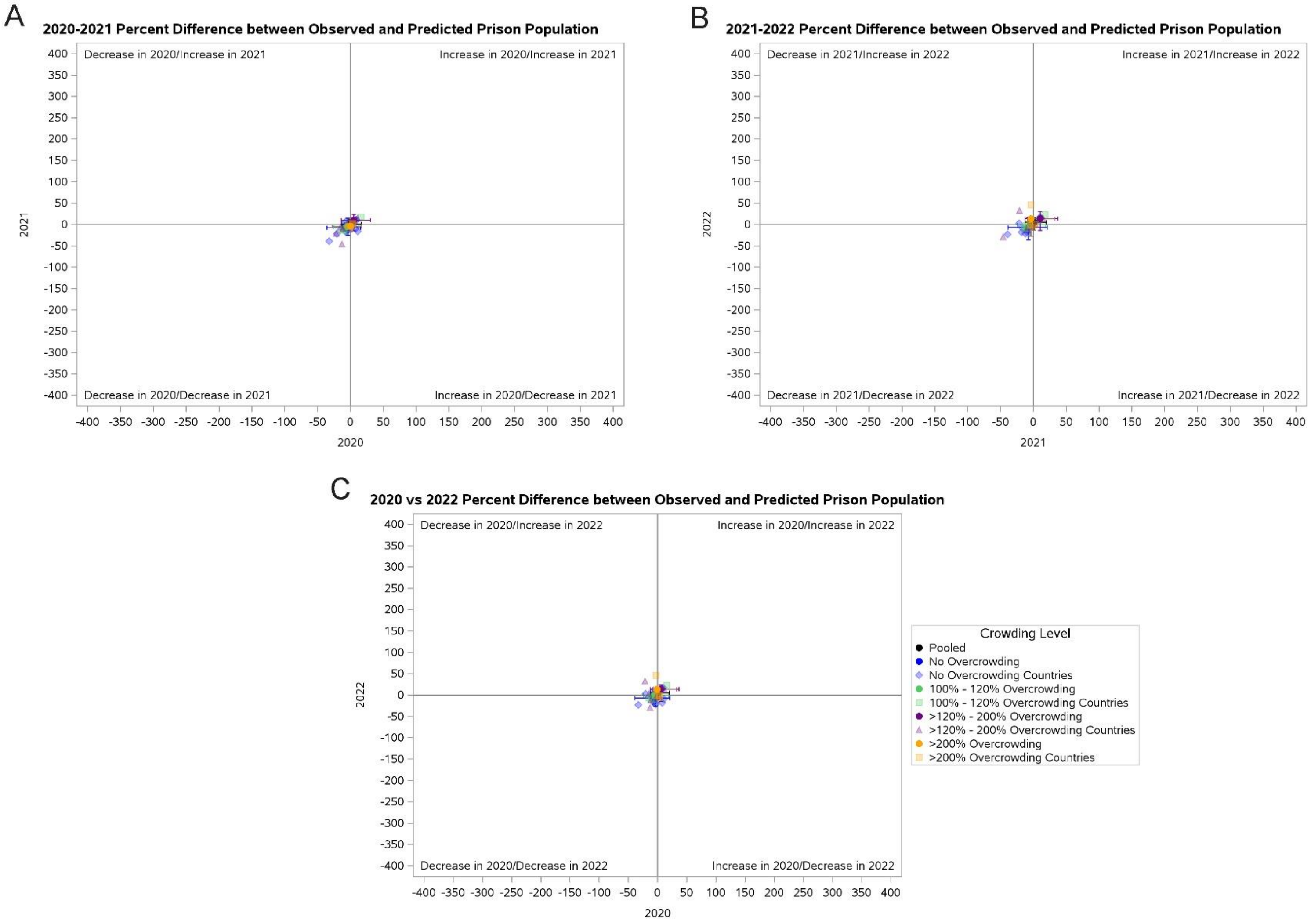

Supplemental Figure S10. *Scatterplot comparing the percent difference in observed vs. predicted tuberculosis notification per 100,000 people who are incarcerated stratified by region in A) 2020 vs. 2021, B) 2021 vs. 2022, and C) 2020 vs. 2022. To be included countries had to have data from 2020-2022, i.e., all three years the pandemic.*

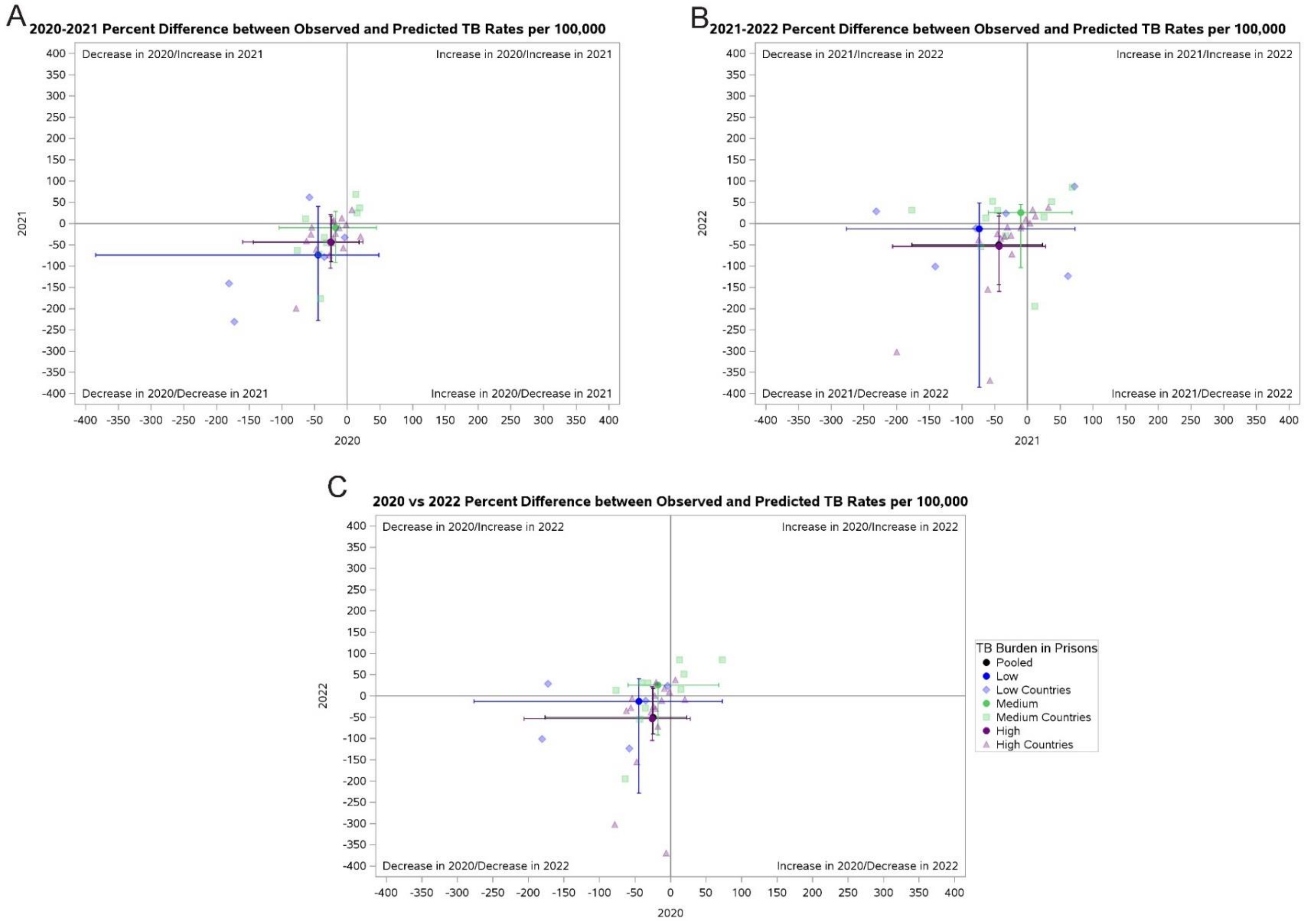

Supplemental Figure S11. *Scatterplot comparing the percent difference in observed vs. predicted prison population stratified by tuberculosis notification rate among the prison population in A) 2020 vs. 2021, B) 2021 vs. 2022, and C) 2020 vs. 2022. To be included countries had to have data from 2020-2022, i.e., all three years of the pandemic.*

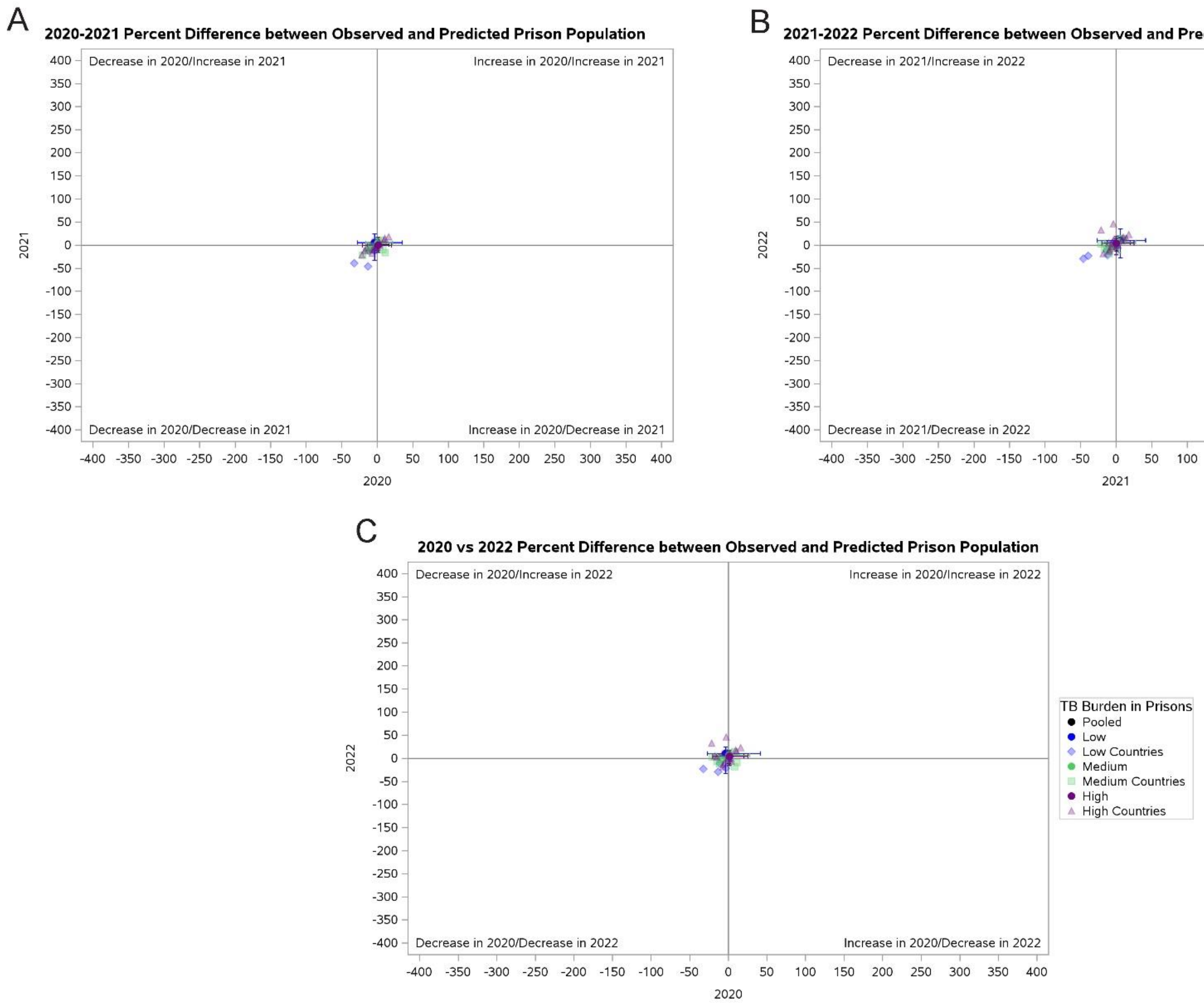

Supplemental Section 4: STROBE Statement Checklist of items that should be included in reports of observational studies

	Item No.	Recommendation	Page/Paragraph No.
**Title and abstract**	1	(*a*) Indicate the study's design with a commonly used term in the title or the abstract	3/Methods
		(*b*) Provide in the abstract an informative and balanced summary of what was done and what was found	3/Methods and Findings
**Introduction**			
Background/rationale	2	Explain the scientific background and rationale for the investigation being reported	5/1-2
Objectives	3	State specific objectives, including any prespecified hypotheses	6/3
**Methods**			
Study design	4	Present key elements of study design early in the paper	7/4
Setting	5	Describe the setting, locations, and relevant dates, including periods of recruitment, exposure, follow-up, and data collection	7/1-3
Participants	6	(*a*) *Cohort study*—Give the eligibility criteria, and the sources and methods of selection of participants. Describe methods of follow-up<br>*Case-control study*—Give the eligibility criteria, and the sources and methods of case ascertainment and control selection. Give the rationale for the choice of cases and controls<br>*Cross-sectional study*—Give the eligibility criteria, and the sources and methods of selection of participants	7/1-4
		(*b*) *Cohort study*—For matched studies, give matching criteria and number of exposed and unexposed<br>*Case-control study*—For matched studies, give matching criteria and the number of controls per case	N/A
Variables	7	Clearly define all outcomes, exposures, predictors, potential confounders, and effect modifiers. Give diagnostic criteria, if applicable	8/5-6<br>9/1
Data sources/ measurement	8*	For each variable of interest, give sources of data and details of methods of assessment (measurement). Describe comparability of assessment methods if there is more than one group	7/1-3
Bias	9	Describe any efforts to address potential sources of bias	7-8/4-7
Study size	10	Explain how the study size was arrived at	10/1

Continued on next page

Quantitative variables	11	Explain how quantitative variables were handled in the analyses. If applicable, describe which groupings were chosen and why	9/1
Statistical methods	12	(*a*) Describe all statistical methods, including those used to control for confounding	7-9/4-9
		(*b*) Describe any methods used to examine subgroups and interactions	9/1
		(*c*) Explain how missing data were addressed	8/2-3
		(*d*) *Cohort study*—If applicable, explain how loss to follow-up was addressed<br>*Case-control study*—If applicable, explain how matching of cases and controls was addressed<br>*Cross-sectional study*—If applicable, describe analytical methods taking account of sampling strategy	N/A
		(*e*) Describe any sensitivity analyses	N/A
Participants	13*	(a) Report numbers of individuals at each stage of study—eg numbers potentially eligible, examined for eligibility, confirmed eligible, included in the study, completing follow-up, and analysed	10/1<br>Table 1<br>Figure 1<br>Supplemental Section 3 Table 1
		(b) Give reasons for non-participation at each stage	10/1<br>Figure 1
		(c) Consider use of a flow diagram	Supplemental Section 1 Figure 1
Descriptive data	14*	(a) Give characteristics of study participants (eg demographic, clinical, social) and information on exposures and potential confounders	10/1-2<br>Table 1<br>Figure 1
		(b) Indicate number of participants with missing data for each variable of interest	10/1<br>Table 1
		(c) *Cohort study*—Summarise follow-up time (eg, average and total amount)	Table 1
Outcome data	15*	*Cohort study*—Report numbers of outcome events or summary measures over time	11-13
		Case-control study—Report numbers in each exposure category, or summary measures of exposure	N/A
		Cross-sectional study—Report numbers of outcome events or summary measures	N/A

Main results	16	(*a*) Give unadjusted estimates and, if applicable, confounder-adjusted estimates and their precision (eg, 95% confidence interval). Make clear which confounders were adjusted for and why they were included	11-13<br>Table 2
		(*b*) Report category boundaries when continuous variables were categorized	11-13<br>Table 2
		(*c*) If relevant, consider translating estimates of relative risk into absolute risk for a meaningful time period	N/A
Other analyses	17	Report other analyses done—eg analyses of subgroups and interactions, and sensitivity analyses	12-13<br>Figure 2<br>Supplemental Section 3 Tables 5-6<br>Supplemental Section 3 Figures 2-11
Key results	18	Summarise key results with reference to study objectives	14/1
Limitations	19	Discuss limitations of the study, taking into account sources of potential bias or imprecision. Discuss both direction and magnitude of any potential bias	15-16
Interpretation	20	Give a cautious overall interpretation of results considering objectives, limitations, multiplicity of analyses, results from similar studies, and other relevant evidence	15-16
Generalisability	21	Discuss the generalisability (external validity) of the study results	15-16
Other information			
Funding	22	Give the source of funding and the role of the funders for the present study and, if applicable, for the original study on which the present article is based	18

*Give information separately for cases and controls in case-control studies and, if applicable, for exposed and unexposed groups in cohort and cross-sectional studies.

Note: An Explanation and Elaboration article discusses each checklist item and gives methodological background and published exampl es of transparent reporting. The STROBE checklist is best used in conjunction with this article (freely available on the Web sites of PLoS Medicine at http://www.plosmedicine.org/, Annals of Internal Medicine at http://www.annals.org/, and Epidemiology at http://www.epidem.com/). Information on the STROBE Initiative is available at www.strobe-statement.org.
